# Design and Synthesis of Arf1-Targeting γ-Dipeptides as Potential Agents against Head and Neck Squamous Cell Carcinoma

**DOI:** 10.3390/cells9020286

**Published:** 2020-01-24

**Authors:** Yen Vo-Hoang, Sergio Paiva, Leilei He, Sébastien Estaran, Yong Teng

**Affiliations:** 1 Institut des Biomolécules Max Mousseron, UMR 5247, CNRS-UM-ENSCM, UFR des Sciences Pharmaceutiques et Biologiques, 15 avenue Charles Flahault, 34093 Montpellier CEDEX 5, France; sergiopaiva00@gmail.com (S.P.); sebastien.estaran@umontpellier.fr (S.E.); 2 Department of Oral Biology and Diagnostic Sciences, Georgia Cancer Center, Augusta University, 1120 15th Street, Augusta, GA 309212, USA; lehe@augusta.edu

**Keywords:** HNSCC, Arf1, constrained γ-dipeptides, anticancer

## Abstract

Background: Head and neck squamous cell carcinoma (HNSCC) is one of the leading causes of cancer-related deaths and calls for new druggable targets. We have previously highlighted the critical role of ADP-ribosylation factor-1 (Arf1) activation in HNSCC. In the present study, we address the question whether targeting Arf1 could be proposed as a valuable strategy against HNSCC. Methods: We rationally designed and synthesized constrained ATC-based (4-amino-(methyl)-1,3-thiazole-5-carboxylic acid) γ-dipeptides to block Arf1 activation. We evaluated the effects of these γ-dipeptides in HNSCC cells: The cell viability was determined in 2D and 3D cell cultures after 72 h treatment and Arf1 protein levels and activity were assessed by GGA3 pull-down and Western blotting assays. Results: Targeting Arf1 offers a valuable strategy to counter HNSCC. Our new Arf1-targeting compounds revealed a strong in vitro cytotoxicity against HNSCC cells, through inhibiting Arf1 activation and its downstream pathways. Conclusions: Arf1-targeting γ-dipeptides developed in this study may represent a promising targeted therapeutic to improve managing the HNSCC disease.

## 1. Introduction

Cancer is one of the leading causes of death worldwide, with an estimated number of cancer deaths of 9.6 million in 2018 [1,2]. Head and neck squamous cell carcinoma (HNSCC) represents the sixth most common malignancy worldwide, with approximately 500,000 new cases diagnosed each year and remains one of the leading causes of cancer-related death [3,4]. HNSCC patients with advanced stage disease require a multimodal approach combining surgical resection, radiotherapy, and systemic chemotherapy [5,6]. Among HNSCC therapies, cytotoxic agents (e.g., cisplatin, 5-fluorouracil) have revealed some adverse reactions, preventing the use of higher doses. Cisplatin-based chemotherapies have been thus associated with agents targeting the epidermal growth factor receptor (EGFR), such as monoclonal antibodies (e.g., cetuxinab, panitunumab) and tyrosine kinase inhibitors (e.g., erlotinib, geftinib). Despite aggressive treatments, 40% to 60% of the patients relapse with HNSCC and their five-year survival rates remains at 55%–65%. These figures are due to high incidence of locoregional recurrence and metastasis and therapeutic resistance. They highlight the urgent need to identify new druggable targets to improve the overall survival of patients.

The ADP-ribosylation factor-1 (Arf1), a Ras-related small GTPase, has been associated with the Golgi Apparatus and has been recognized to play a crucial role in controlling membrane trafficking pathways [7,8]. Similar to other Arf proteins, Arf1 is regulated by switching between active GTP-bound and inactive GDP-bound conformations. Activation of Arf1 is facilitated by guanine nucleotide exchange factors (GEF), a protein family displaying a conserved central Sec7 domain responsible for the exchange activity [9]. The expression/activation of Arf1 and its GEFs are often associated with cell proliferation, migration, and tumor-cell invasion in various cancers [10], and Arf1 and Arf/GEF complexes have thus been proposed as valuable candidate targets in cancer therapy [11,12,13]. In our previous study, the similar levels of Arf1 protein were detected among all HNSCC cell lines examined [14]. However, higher levels of active GTP-bound Arf1 were seen in metastatic HN12 and HN31 cells compared with their paired nonmetastatic HN4 and HN30 cells [14], suggesting the correlation of Arf1 activity with HNSCC cell aggressiveness. Moreover, high levels of Arf1 activity are maintained by binding to phosphorylated EGFR on the HNSCC cell plasma membrane. Decreased EGFR phosphorylation inactivates Arf1 and eventually inhibits its mediated invasion in HNSCC cells [14]. Given the critical role of Arf1 activation in HNSCC growth and metastasis, directly inhibiting it or disassociating Arf1 protein from EGFR could be a promising strategy counteracting HNSCC.

**Figure 1:**
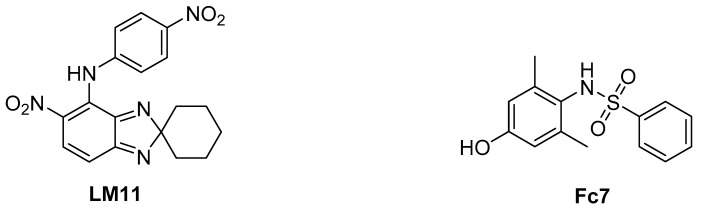
Structure of **LM11** and **Fc7**, two inhibitors of ADP-ribosylation factor-1 (Arf1) activation by Arf nucleotide-binding site opener (Arno).

Over the last decades, several inhibitors with unrelated chemical structures have been discovered to inhibit the GEF-dependent activation of Arf1. The first known inhibitor of GEFs is the paradigm interfacial uncompetitive inhibitor Brefeldin A (BFA), a fungal metabolite isolated in the late 1950 [15]. BFA binds to the Arf1-GDP/Sec7 complex and stabilizes an abortive complex at an early stage of the reaction, prior to guanine nucleotide release [16]. While not all Arf GEFs are sensitive, BFA demonstrated that small GTPases and their GEFs are druggable targets. The binding mode of BFA inspired the in silico selection of an interfacial Arf1-GDP/GEF inhibitor so called LM11 (Figure 1) [17]. Virtual screening has been performed starting from the complex formed by Arf1 and Arf nucleotide-binding site opener (Arno), a cytohesin member of the GEF family, characterized by a Pleckstrin homology (PH) domain. By inhibiting the activation of Arf1, BFA and LM11 exert a cytotoxic activity and LM11 blocks the development of metastasis of an invasive breast cancer model [18,19]. Since then, many other modulators, i.e., AMF-26 [20], SecinH3 [21,22], Exo2 [11,23], and analogues resulting from virtual, biochemical, and phenotypic screening approaches have been reported. In a previous study using a fragment-based drug design approach, Chavanieu et al. have determined the binding mode of small molecules at the surface of the Sec7 domain of Arno [24]. Among the selected fragments, a benzenesulphonamide ligand (i.e., Fc7, Figure 1) acts as a protein–protein interaction inhibitor with an IC_50_ in the millimolar range. XRD structural analysis confirmed that Fc7 interacts with a locus formed by L148, L153, I160, and I193 in the α6, α7, and α8 helices of the Sec7 domain thus interfering with the Arf1 binding site on the Arno Sec7 surface. By analogy with general receptor of phosphoinositides-1 (GRP1), another member of the same GEF subfamily, this hydrophobic hot spot zone has also been recognized as an interacting region for an autoinhibitory sequence positioned between the Sec7 and PH domains of Arno. In GRP1, the 257-DLTYTF-262 sequence of the Sec7/PH linker blocks the binding sites for the switch 1 and switch 2 regions of Arf proteins through a pseudosubstrate mechanism [25]. On human Arno, the equivalent autoinhibitory sequence presents a tyrosine to histidine mutation (252-DLTHTF-257) [26]. Following our efforts to interfere with Arf1-GDP/Sec7-Arno, we explored the ability of constrained heteroaromatic γ-dipeptide scaffolds, so called 4-amino-(methyl)-1,3-thiazole-5-carboxylic acids (ATCs) to template the key interacting elements of the Arno autoinhibitory domain. Based on a rational design, we synthesized eleven ATC-based derivatives displaying three diversity points as mimetic of the 252-DLTHTF-257 sequence. We herein report their chemical synthesis, as well as the evaluation of their potential cytotoxic effect in HNSCC cells. 

## 2. Materials and Methods

All reagents (e.g., protected amino acids of carbonyldiimidazole) and solvents (e.g., anhydrous tetrahydrofuran) were purchased from Sigma-Aldrich Chemie (Saint-Quentin Fallavier, France) and used as received without further purification. Reactions were monitored by thin-layer chromatography (TLC) and analytical high performance liquid chromatography (HPLC) Analytical TLCs was performed on 60 F254 aluminium-backed plates (Sigma-Aldrich Chemie, Saint-Quentin Fallavier, France)). Products were purified by column chromatography by using a Merck Kieselgel 60 plate (230–400 mesh) (Sigma-Aldrich Chemie, Saint-Quentin Fallavier, France)) or by flash column chromatography on silica gel on a Biotage Isolera One system. Plates were visualized by UV light (λ = 254 nm) and by heating at 300 °C after spraying with a commercial solution of phosphomolybdic acid [20% in ethanol (EtOH)]. Analytical HPLC was performed with an Alliance HT Waters 2795 separations module equipped with a Chromolith Speed Rod RP-C18 column (50V4.6 mm, 5 mm) with gradient elution from 100% H_2_O/trifluoroacetic acid (TFA) 0.1% to 100% acetonitrile (CH_3_CN)**/** TFA 0.1% over 3 min and a flow rate of 5 mL/min. UV detection at λ = 214 and 254 nm was enabled with a Waters 996 photodiode array detector and retention times (tr) in minutes are reported. Liquid chromatography–mass spectrometry (LC-MS) analyses were recorded with a Quattro micro ESI triple quadrupole mass spectrometer (Micromass, Manchester, UK) equipped with a Chromolith Speed Rod RP-C18 column (50V4.6 mm, 5 mm) and an Alliance HPLC System (Waters, Milford, CT, USA) with gradient elution from 100% H_2_O/formic acid 0.1% to 100% CH_3_CNformic 0.1%acid over 3 min, a flow rate of 3 mL/min, and UV detection at λ = 214 nm. HRMS analysis was performed with a TOF mass spectrometer fitted with an ESI source in positive-ion mode. ^1^H- and ^13^C NMR spectra were recorded at room temperature with a Bruker AC-300 spectrometer (Wissembourg, France), or a Bruker Avance 600 AVANCE III spectrometer equipped with a 5 mm quadruple-resonance probe (^1^H, ^13^C, ^15^N, ^31^P) or a Bruker 400 spectrometer at 400.13 and 100.62 MHz, respectively; chemical shifts (δ) are reported in parts per million (ppm) relative to the solvent (^1^H: δ (CDCl_3_) = 7.26 ppm; ^13^C: δ (CDCl_3_) = 77.16 ppm; ^1^H: δ ([D_6_]DMSO) = 2.50 ppm; ^13^C: δ ([D_6_]DMSO) = 39.52 ppm). NMR spectra are reported as follows: δ (Signal multiplicity, coupling constant(s), number of protons, proton assignment). ^1^H NMR signal multiplicities are designated as broad (br), singlet (s), doublet (d), doublet of doublets (dd), triplet (t), triplet of doublets (td), quadruplet (q), quintet (quin), multiplet (m), sextet (sext), octuplet (oct), and nonet (non).

### 2.1. Synthesis of Compounds **8a**–**d**, **9a**–**c**, **10a**–**d**

Fmoc-ATC-OH were prepared according to the reported procedures (Fmoc = 9-fluorenylmethoxycarbonyl) [27,28]. The synthesis of compounds **8a**–**d**, **9a**–**c**, **10a**–**d** is described in the Supporting Information.

### 2.2. NMR Experiments

The NMR samples contained **9a**,**b** and **10a**–**c** dissolved in CDCl_3_ or CD_3_OH. Homonuclear 2D spectra DQF-COSY, TOCSY (DIPSI2), and ROESY were typically recorded in the phase-sensitive mode by using the States-TPPI method as data matrices of 256–512 real (*t*1) V 2048 (*t*2) complex data points; 8–48 scans per *t*1 increment with a 1.0 s recovery delay and spectral width of 7210 Hz in both dimensions were used. The mixing times were 80 ms for TOCSY and 350 ms for ROESY experiments. Spectra were processed with Topspin software (Bruker Biospin) and visualized with Topspin or NMRView on a Linux station. The matrices were zero-filled to 1024 (*t*1) V 2048 (*t*2) points after apodization by shifted sine-square multiplication and linear prediction in the F1 domain. Chemical shifts were referenced to tetramethylsilane (TMS). ^1^H NMR chemical shifts were assigned according to classical procedures. Nuclear overhauser effect (NOE) cross-peaks were integrated and assigned within the NMRView software. The volume of a rotating-frame overhauser enhancement (ROE) between methylene pair protons was used as a reference of 1.8 Å. The lower bound for all restraints was fixed at 1.8 Å and the upper bounds at 2.7, 3.3, and 5.0 Å, for strong, medium, and weak correlations, respectively. Pseudo-atom corrections of the upper bounds were applied for unresolved aromatic, methylene, and methyl protons signals as described previously [29].

### 2.3. Cell Culture and Cell Viability

HNSCC cell lines HN4, HN12, and HN8 were a gift from Dr. W. Andrew Yeudall and used for experiments before passage 10 [14]. All cells were maintained in Dulbecco’s modified Eagle’s medium (DMEM) containing 10% fetal bovine serum at 37 °C in a humidified incubator supplied with 5% CO_2_. Cell viability was determined by CellTiter-Glo^®^ Luminescent cell viability assay (Promega, Madison, MI) according to the manufacturer’s instructions.

### 2.4. Three-Dimensional (3D) Cell Cultures

HN12 cells were seeded into 48-well SeedEZ (Lena Bioscience, Atlanta, GA) supplied with a complete medium. Four days after culture, cells growing in SeedEZ were exposed to compound **10b** for 72 h, and cell viability was then measured by alamarBlue at 545/590 nm ex/em, followed by 4′,6-diamidino-2-phenylindole (DAPI, Sigma) staining and imaging. 

### 2.5. Western Blotting and Determination of Arf1 Activation

Western blotting was performed as we previously described [18]. Briefly, electrophoresis was performed on 12% SDS-PAGE gel and the proteins were transferred to a nitrocellulose membrane. The membranes were incubated with the primary antibodies against Arf1 (Abcam) or β-Actin (Sigma) overnight at 4 °C and with secondary antibodies for 1 h at room temperature. To visualize peroxidase activity, ECL reagents (Bio-Rad) were used according to the manufacturer’s instructions. For determination of Arf1 activation, the GST-GGA3-GAT pull-down assay was performed, followed by Western blotting as described previously [18]. Briefly, HN12 cells were treated with or without compound **10b** for 24 h. Lysates were prepared in a GGA3 pull-down buffer [50 mM Tris (pH 7.5), 150 mM NaCl, 10 mM MgCl_2_, 10% glycerol, 1× protease inhibitor cocktail, and 1% Triton X-100] then incubated in 20 μL of GST-GGA3-GAT-bound glutathione-Sepharose beads for 1 h at 4 °C. Subsequently, beads were washed three times in a pull-down buffer and proteins associated with the beads were subjected to Western blotting with antibodies against GST (Sigma) and Arf1 (Abcam).

### 2.6. Statistics

Qualitative data including immunoblots and images are representatives of at least three independent experiments. Quantitative data were expressed as means ± standard error of mean (SEM). Statistical differences between two groups were determined by the two-tailed unpaired or paired Student t-test. *p* < 0.05 was considered significantly different.

## 3. Results

### 3.1. Rational Design of γ-Dipeptides

**Figure 2:**
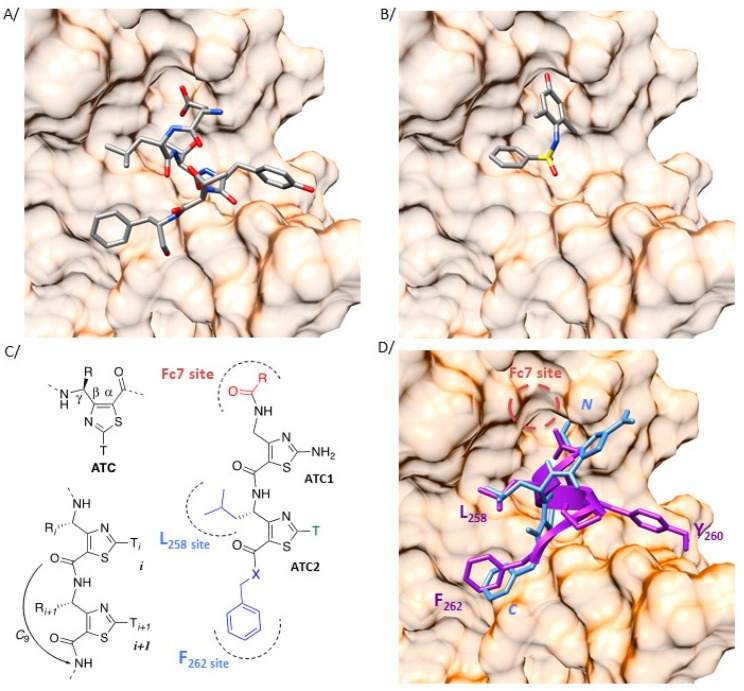
Heteroaromatic γ-dipeptide to mimic the auto inhibitory domain of cytohesin proteins. (**A**) Crystal structure (2R09) of the region of the guanine nucleotide exchange factor general receptor of phosphoinositides-1 (GEF GRP1) interacting with the switch I and switch II regions of Arf proteins (surface representation). The intrinsic autoinhibitory peptide of GRP1 (257-DLTYTF-262) is represented in stick (stick representation, colored by elements with carbon in grey, oxygen in red, nitrogen in blue, and sulfur in yellow). (**B**) Crystallographic pose (4JWL) of Fc7 (stick representation, colored by elements, as previously described) at the same region of the Sec7 domain of Arno (surface representation). (**C**) Nomenclature of 4-amino-(methyl)-1,3-thiazole-5-carboxylic acid (ATC) γ-amino acids and characteristic H-bonding network of the oligomers. In the designed ATC dipeptides, the substituents in blue point towards the L258 and F262 binding sites while the Fc7 binding site is targeted by the *N*-ter extremity (red). (**D**) The intrinsic autoinhibitory peptide of GRP1 (257-DLTYTF-262) and the γ-dipeptide based mimic are represented in purple ribbon and blue stick, respectively. The three-dimensional (3D) side chain projection around the ATC scaffold was determined from the ATC XRD structure (CSD entry AGEZAP/922304). The Fc7 binding site is indicated as a red dash circle.

Similar to other GEF members of the cytohesin family, Arno is composed of a coiled-coil region responsible for dimerization and interaction with other proteins and two domains namely the Sec7 domain and the C-terminal PH domain [30]. The Sec7 domain is involved in the guanine nucleotide exchange and is considered as the catalytic domain of cytohesins. The PH domain binds specific phosphatidylinositol phosphates and thus contributes in recruiting proteins to membranes [31]. Structural determination in the autoinhibited conformation of Mus musculus GRP1, revealed that a linker region localized between these Sec7 and PH domains plays a role in a pseudosubstrate mechanism of autoinhibition [25]. The linker region of GRP1, mainly the sequence 257-DLTYTF-262 blocks the binding sites for the switch I and switch II regions of Arf proteins [25]. On Arno, the equivalent autoinhibitory is 252-DLTHTF-257 (Figure 2 A) [26]. 

One major interest of our group concerns the design of new highly predictable and stable molecular pseudo-peptide architectures for therapeutic applications [32]. In such a context, we described a class of constrained heterocyclic γ-amino acids built around a thiazole ring, named ATCs (Figure 2C) [27]. ATC oligomers are helical molecules resulting from the planar conformation of the γ-β-α-*C*(*O*) torsion angle given by the thiazole heterocycle (Figure 2C) and the formation of a highly stable *C_9_* hydrogen-bonding pattern. Noteworthy, it was recognized that only two residues were necessary to initiate the *C*_9_ pseudo-cycles folding. Based on 3D superimposition of a canonical γ-dipeptide model and of the 257-DLTYTF-262 peptide from GRP1 (pdb 2R09), we established that the ATC scaffold might deliver side chain projections in suitable positions to mimic the key interacting elements of the autoinhibitory domain (Figure 2D). For instance, the *iso*propyl lateral chain at the γ-position of ATC2 aims to mimic the L258 residue of the autoinhibitory peptide and the *C*-term benzyl group is superimposed to the F262 amino acid. The *N*-terminus extremity is matching with the Fc7 binding site (Figure 2B) [24].

### 3.2. Synthesis of γ-Dipeptides

**Scheme 1:**
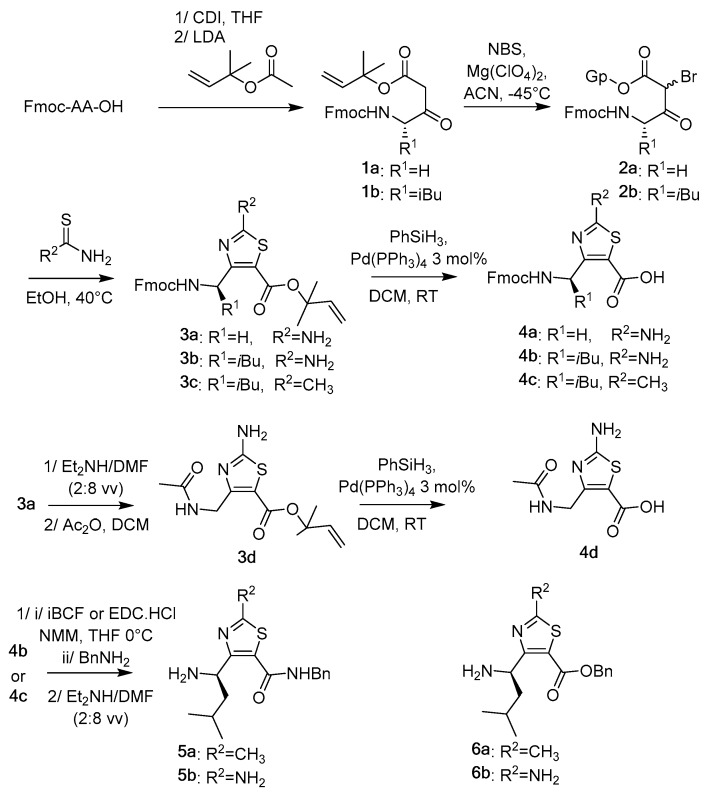
Synthesis of the ATC monomer building blocks**.**

*N-*Fmoc-ATC-OH monomer units **4a**–**d** were synthesized from the commercially available *N*-Fmoc-Gly-OH and *N*-Fmoc-Leu-OH following our previously reported methodology (Scheme 1). The *N*-Fmoc-amino acids were first engaged in a cross-Claisen condensation to lead the β-ketoesters **1a**–**b**. The malonic position of β-ketoesters was monobrominated in acetonitrile (ACN) using *N*-bromosuccinimide (NBS) and magnesium perchlorate [Mg(ClO_4_)_2_] as catalyst. The resulting α-bromo-β-ketoesters **2a**–**b** were engaged in Hantzsch heterocyclisation using thiourea or thioacetamide in EtOH to give a fully protected ATCs **3a**–**c**. After removal of the Fmoc group of **3a** with a solution of diethylamine in *N,N*-dimethylformamide (DMF) (2:8 v/v), the *N*-acetylated ATC-OH **3d** was obtained using acetic anhydride. Quantitative removals of dimethylallyl esters were carried out with 3 mol% Pd(PPh_3_)_4_ and phenylsilane to yield the *N-*Fmoc-ATC-OH **4a**–**c** and the *N*-acetyl-ATC-OH **4d**. ATC-NHBn **5a** and **5b** were obtained in 95% and 20% from **4b** and **4c,** respectively using isobutyl chloroformate (IBCF, 1.2 equivalents) or *N*-Ethyl-*N*′-(3-dimethylaminopropyl)carbodiimide hydrochloride (EDC.HCl, 2 equivalents) as an activating reagent and benzylamine. Fmoc groups were removed with a solution of diethylamine in DMF (2:8 *v*/*v*). Access to *N-*Fmoc-ATC-OBn **6a,b** has been done according to procedures reported by L. Mathieu [27].

**Scheme 2:**
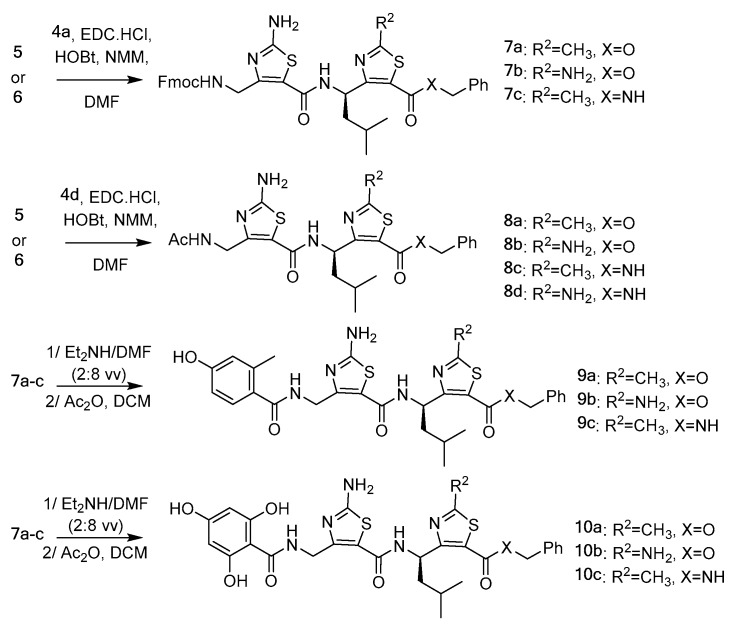
Synthesis of the ATC-based γ-dipeptides.

The Fmoc protected γ-peptidic dimers **7a**–**c** and the *N*-acetylated dimers **8a**–**d** were then prepared by coupling, respectively *N-*Fmoc-ATC-OH **4a** or *N*-acetyl-ATC-OH **4d** and H_2_N-ATC-NHBn **5a,b** or H_2_N-ATC-OBn **6a,b** monomers using EDC.HCl (1.2 equivalents), 1-hydroxybenzotriazole hydrate (HOBt, 1.2 equivalents) and *N*-methylmorpholine (NMM, 1.2 equivalents) as base in DMF, as reported in Scheme 2. Fmoc removal followed by acylation with appropriate benzoic acids led to the targeted γ-dipeptides **9a**–**c** and **10a**–**c**.

**Scheme 3:**
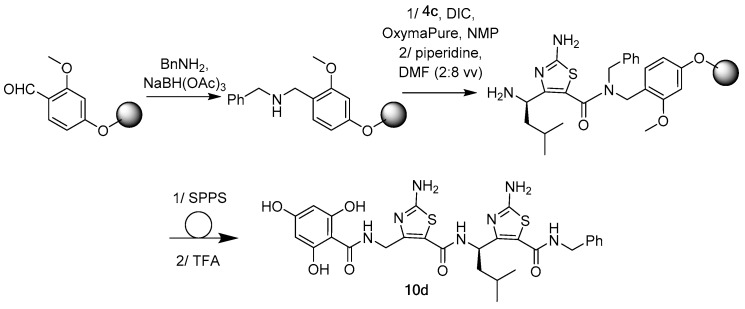
Solid phase peptide synthesis (SPPS) approach to access ATC-based γ-dipeptide **10d**.

Solid phase peptide synthesis (SPPS) was also considered as an alternative strategy (Scheme 3). The acid sensitive methoxybenzaldehyde (AMEBA) polystyrene resin (0.86 mmol·g^−1^) was used as solid support [33]. Reductive amination using 2.0 equivalents of benzylamine and NaBH(OAc)_3_ gave the corresponding amine resin. Oligomerization was then performed using a mixture of ethyl (hydroxyimino) cyanoacetate (oxyma Pure^®^, 2.0 equivalents), and *N,N*′-diisopropylcarbodiimide (DIC, 2.0 equivalents) as coupling reagents in *N*-methyl-2-pyrrolidone (NMP) overnight followed by Fmoc removal using a solution of piperidine in DMF (2:8 v/v) [29]. After *N*-acylation using 2,4,6-trihydroxybenzoic acid (3 equivalents), EDC.HCl (3 equivalents), NMM (3 equivalents), HOBt (3 equivalents), the targeted dimer **10d** was recovered in 10% yield after cleavage with TFA and purification on preparative RP-HPLC. 

### 3.3. Structure Analysis

**Figure 3:**
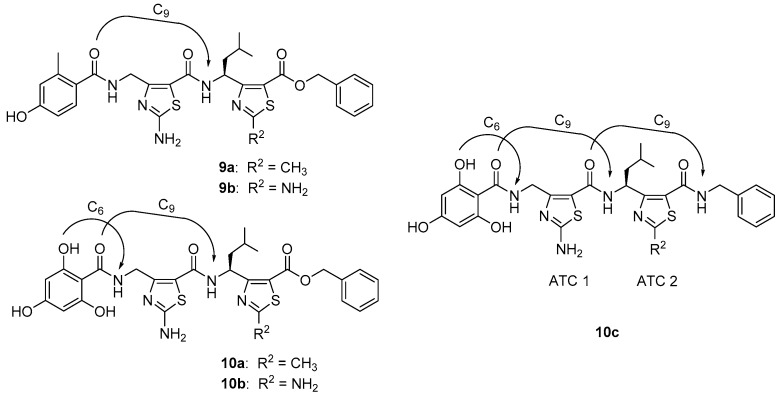
Characteristic H-bonding network of constrained γ-peptidic dimers **9a,b** and **10a–c**.

The folding propensity of **9a**, **9b**, **10a**, **10b,** and **10c** was studied by NMR in CD_3_OH (Supplementary Information) as illustrated in Figure 3. In the case of **9a,b**, de-shielding effects were observed for the ATC 2 amide protons (δ = 9.28 to 9.52 ppm depending on the molecule). As previously observed on other ATC oligomers [29,34], such a behavior is a strong indicator of the participation of the amide protons in a C_9_ hydrogen-bond. By contrast, the signal of ATC1 NH was around 8.5 ppm in line to what is expected for a free NH. In the case of **10a**–**c**, both ATC 1 and ATC2 NH were strongly de-shielded (δ = 9.72 to 9.95 ppm depending on the molecule) suggesting that they were all engaged in the H-bond. While such effect was expected for the ATC2 NH, it was not usual for the *N*-terminus amide proton. It is likely that the NH is bonded to one OH of the trihydroxyphenyl moiety making a six-member pseudocycle. Finally, it is noteworthy that the benzylamide NH in **10c** is also downfield (δ = 9.86 ppm) that is consistent with a third H-bond occurring with the ATC1 C=O.

In addition to specific ATC NH resonances, the analysis of coupling constants revealed characteristic values ^3^*J*(NH,^γ^CH) < 6.5 Hz for ATC1 NH of most of the studied dimers (**9a**, **10a,c**). These coupling constant values around 5.6 (Tables S2, S7, and S13, Supporting Information) were typical of *φ* values around −80° in accordance with a C_9_-helical shape for the γ-peptide skeletons. In the case of **9b** and **10b**, coupling constant values ^3^*J*(NH,^γ^CH) could not be determined probably due to signal broadening. In the case of **10c**, a supplementary H-bond involved the benzylamide NH. However, no characteristic values ^3^*J*(NH,^γ^CH) < 6.5 Hz were observed for ATC2 NH. The unexpected coupling constant value (^3^*J*(NH,^γ^CH) = 7.2 Hz) showed a fraying of dihedral angle.

**Figure 4:**
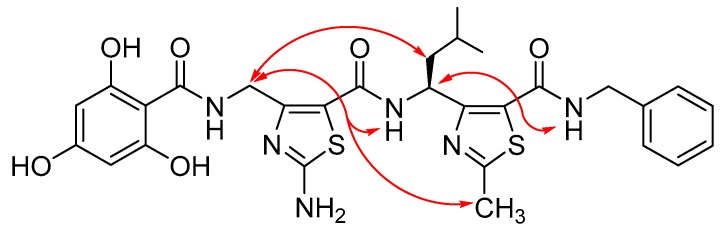
Characteristic nuclear overhauser effect (NOE) correlations observed for typical constrained γ-peptidic dimer **10c.**LP24.

Lastly, 2D NMR experiments were performed to analyze NOE correlations (Tables S5, S8, S11, and S14, Supplementary Information). Characteristic medium ^γ^CH(1)/NH(2) connectivities were observed for all studied γ-dimers (**9b**, **10a**, **10b,** and **10c**). A supplementary strong ^γ^CH(2)/NHBn connectivity was also noted. These NOE cross-peaks between NH(i)/^γ^CH(i-1) have been recognized as structural markers of the 3D organization of ATC oligomers and corroborate the constrained structure of the dimers (Figure 4). Furthermore, two γ-dimers **10a** and **10c** revealed weak ^γ^CH (1)/^δ^CH(2) connectivities, as well as weak intensity NOEs between the thiazole side chain of ATC2 and the ATC 1 ^γ^CH (^γ^CH (1)/^ϴ^CH(2)). These data are compatible with the previously reported C_9_-helical shape. 

### 3.4. Compound **10b** Exhibits Superior Anticancer Effect in HNSCC Cells by Inactivating Arf1

**Figure 5:**
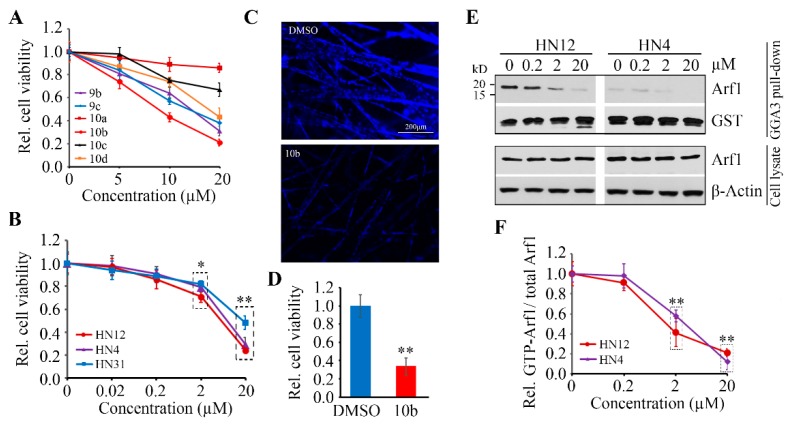
The anticancer effects of Arf1-targeting compounds. (**A**) The effects of Arf1-targeting compounds (**9b**, **9c**, **10a**, **10b**, **10c**, and **10d** in a range dose from 0 to 20 µM) on HN12 cell viability were determined by CellTiter-Glo^®^ Luminescent cell viability assay after 72 h treatment. (**B**) The dose-dependent cytotoxicity of compound **10b** in three HNSCC cell lines (HN4, HN12, and HN31) was determined by CellTiter-Glo^®^ Luminescent cell viability assay after 72 h treatment. (**C,D**) The effects of 20 µM of compound **10b** on HN12 cell viability in SeedEZ was determined after 72 h treatment. Representative images of DAPI staining and quantitative data of cell viability measured by alamarBlue are shown in (**C**,**D**), respectively. (**E**,**F**) Arf1 protein levels and its activity in HN12 and HN4 cells treated with or without compound **10b** were determined by GGA3 pull-down assay and Western blotting. A representative result and quantitative data from three independent experiments are shown in (**E**,**F**), respectively. * *p* < 0.05; ** *p* < 0.01.

To determine whether these new Arf1-targeting compounds have the anti-HNSCC ability, we evaluated the viability of HN12 cells after exposure to γ-dipeptides **8**–**10** at a range dose from 0 to 20 µM. Among the *N*-acetylated γ-dipeptides **8a**–**d**, none revealed any effect on cell viability at the tested dose. The too weak aqueous solubility of compound **9a** did not allow its biological evaluation (data not shown). When HN12 cells were exposed to **10a** and **10c**, even at 20 µM, the relative cell viability was not remarkably diminished (Figure 5A). On the contrary, the anticancer effect of exposure to **9b**–**c**, **10b,** and **10d** was much stronger, showing that the 4-hydroxyphenyl moiety is important for biological activity (Figure 5A). Moreover, it should be noted that **9b**, **10b,** and **10d** shared the NH_2_ group on the thiazole ring, which seemed to be favorable for drug cytotoxicity. Since this NH_2_ group is not supposed to interact with the Arno surface, this effect may be linked to the modulation of aqueous solubility of γ-dipeptides, facilitating their cellular permeability.

Among the tested compounds, the γ-dipeptide **10b** displayed strongest cytotoxicity (Figure 5A), and thus we continued our investigations mainly on this molecule **10b**. The further analysis showed that the IC_50_ of compound **10b** in HN12 cells was around 10 µM, which was the same to that in HN4 cells (Figure 5B). We also observed the inhibitory effect of compound **10b** in HN31 cells, though the IC_50_ in this cell line was higher (~ 20 µM) than HN12 and HN4 cells (Figure 5B). 3D cell culture has the potential to mimic the natural in vivo setting better than the traditional monolayer 2D cell culture, which better mirrors the in vivo responses to anticancer drugs. We then turned to 3D cultures using the SeedEZ scaffold, in which cell viability were suppressed significantly by compound **10b** compared with DMSO (Figure 5C,D). These data further support the in vitro efficacy of compound **10b** in counteracting HNSCC cells.

We next determined levels of Arf1 protein and activation status in HNSCC cells treated with or without compound **10b**. This treatment did not affect the protein levels of Arf1 (Figure 5E,F). However, compound **10b** significantly inhibited Arf1 activation in both HN12 and HN4 cells, and this effect was dose dependent as evidenced by the less active Arf1 form that was detected in high dose treated cells compared with low dose treated cells (Figure 5E,F). These findings suggest that the cytotoxicity of compound **10b** is Arf1-specific in HNSCC cells.

## 4. Discussion

HNSCC arises from nasal cavity, paranasal sinuses, oral cavity, salivary glands, pharynx, and larynx. Tobacco and alcohol consumption and human papillomavirus (HPV) infection have been identified as the critical risk factors of HNSCC, which are responsible for the majority of HNSCC burden [4]. HNSCCs constitute a heterogenous group of aggressive cancers, and its diversity can be noted in the various locations of HNSCC in the upper aerodigestive tract, as well as at the molecular level [35]. Indeed, HNSCC has a relatively high tumor mutation burden, leading to genetically complex cancers. The heterogeneous nature of HNSCC calls for various treatment approaches [35]. Recent developments towards understanding the HNSCC disease have led to identify various targets and to develop the corresponding targeted therapies [36]. EGFR-targeted therapies, such as antisense gene therapy, monoclonal antibodies, and tyrosine kinase inhibitors, were first approved for HNSCC treatment [11,14]. To date, different inhibitory immune checkpoints have been studied, including programmed cell death protein 1 (PD-1) and cytotoxic T-lymphocyte protein 4 (CTLA4). Immune checkpoint inhibitors aiming to hinder the inhibitory interaction between PD-1 and its ligand PD-L1 have been developed, though high PD-L1 expression in the tumor cells did not correlate with poor prognosis of patients suffering from oral squamous cells carcinoma [37,38,39]. In 2016, nivolumab and pembrolizumab, the humanized antibodies targeting PD-1, were FDA-approved to treat recurrent/metastatic HNSCC [37,38]. Moreover, clinical trials have also been conducted to test the efficacy of several compounds, such as COTI-2, a small molecule restoring the p53 function altered in 80% of HPV-negative HNSCCs [40]. Even if these molecules have not been approved yet, the exploration of various strategies are needed to develop potential treatments that would be used according to genetic alterations, or clinical profiles of HNSCC patients.

Among the Arf family, Arf1 and Arf6 are the mostly described isoforms [8]. While Arf6 localizes to the plasma membrane to regulate endocytosis, cytokinesis and actin remodeling, Arf1 is mainly recruited at the Golgi where it regulates translocation of proteins from the trans-Golgi network to plasma membrane, and directly activates signaling molecules. The expression of Arf1 has been reported to be upregulated in multiple cancers, such as breast, prostate, colorectal, gastric, ovarian, or hepatocellular cancers or osteosarcoma [10]. During the last decade, Arf1 has emerged as a key regulator of tumor proliferation in various cancers. For example, in prostate cancer, we have linked Arf1 to the hyperactivation of mitogen activated protein kinase (MAPK) [41]. In colorectal cancer, phosphatase of regenerating liver 3 (PRL-3) interacts with Arf1 to promote cell migration. In breast cancer, Arf1 controls cell proliferation by regulating the retinoblastoma protein [42] and acts as a molecular switch to activate EGF-mediated responses [43]. Moreover, knockdown of Arf1 expression markedly inhibited cell proliferation in invasive breast cancer cells [18]. Consequently, Arf1 seems to be a very compelling target to limit cancer development and inactivating Arf1 may constitute a valuable chemotherapy strategy in this regard.

Arf1 inhibitors have been developed and studied as potential anticancer agents [12,13]. Most existing Arf1 inhibitors block its activation by targeting the Sec7 domain on Arf1-GEFs. Inactivating a subset of Arf1-GEFs has been useful for assessing the Arf1 function. However, Arf1 inhibitors remain a daunting challenge to develop into anticancer drugs. For example, BFA, the first identified Arf1 inhibitor, was a potent inducer of apoptosis in human leukemia cells or colon carcinoma cells, when used at the concentration of 1 µM [19]. However, BFA revealed weak bioavailability and high toxicity [44]. Novel molecules have been developed to overcome these disadvantages. Acting as BFA, AMF-26 induced apoptosis in BSY-1 (human breast cancer) cells when used at 0.1 µM [20]. LM11 induced cytotoxicity against breast cancer cells (IC_50_ = 40 µM) and 10 µM of LM11 suppressed Arno-dependent migration, because of its inhibition of Arf1 functions at the Golgi [18]. Exo2 reduced Arf1 activation but the effect is much weaker. Exo2 induced apoptosis of prostate cancer cells when used at 50 µM [11]. Nevertheless, before this study, no any Arf1 inhibitor has been reported to have anti-HNSCC effects.

In the present study, we provide a novel series of compounds inducing HNSCC cells cytotoxicity via targeting Arf1 activation. These are small molecules, chemically unrelated with other anti-HNSCC agents or with known Arf1 inhibitors. Indeed, they were rationally designed to target a locus on Sec7 domain of Arno protein, the locus being a hot spot zone targeted by crucial interacting residues of the autoinhibitory domain of Arno, as well as by residues of Arf1, before its activation [24,45]. Our γ-dipeptidic compounds combined two aspects: 1) They are constrained ATC-based γ-dimers whose 3D organization allows mimicking the helical organization of the autoinhibitory domain of Arno. 2) The γ-dipeptides presented the 4-hydroxyphenyl group previously identified to be essential for Arno surface recognition. The evaluation of drug cytotoxicity revealed that the 4-hydroxyphenyl moiety derived from fragments previously discovered was necessary for cytotoxicity of γ-dipeptides, suggesting that our compounds mimicking the autoinhibitory peptide would bind within the targeted site. Moreover, thanks to the screening of the six compounds, the introduction of NH_2_ group appears beneficial for activity against HNSCC cells, which is possibly through modulating cellular permeability. It is noteworthy that starting from Fc7, the most potent of the fragments previously described, inhibiting Arf1 activation at the concentration of 2 mM, we aimed to gain higher potency and succeeded to get γ-dipeptides with cytotoxic activity observed at 20 µM. More importantly, our work expands the use of Arf1 inhibitors to HNSCC and constitutes a starting point opening a new opportunity for HNSCC treatment. More efforts need to be made to identify and compare the ability of different compounds to Arf1 inactivation. These results may be useful to establish structure activity relationships and to define a pharmacophore for these Arf1-targeting γ-dipeptides, in order to develop more potent compounds as potential anti-HNSCC agents.

EGFR is a transmembrane receptor with tyrosine kinase activity and is overexpressed in 80%–90% of HNSCC cases. EGFR expression correlates with decreased survival and treatment outcomes. The mechanism of EGFR activation and the role of EGFR signaling in cancer progression have been well studied. Notably, Arf1 has been reported to be activated downstream of the EGFR in triple negative breast cancer cells (TNBC) cells [43]. Since then, several reports have underlined the importance of the Arf1/EGFR axis in cancer cells progression [46,47,48]. In HNSCC, we have previously evidenced direct interaction between Arf1 and phospho-EGFR and highlighted the critical role of the EGFR-Arf1 complex in driving HNSCC progression [14]. In this study, our Arf1-targeting γ-dipeptides revealed anticancer activity in HNSCC cells, which may be linked to the blockade of the EGFR-Arf1 axis, suggesting that targeting the EGFR-Arf1 would constitute a feasible innovative strategy to fight against HNSCC and may provide an alternative strategy over currently used chemotherapy tolerance. Nevertheless, our follow-up investigations aim to further elucidate the mechanisms underlying the cytotoxicity of these new developed compounds.

Metastasis and resistance to conventional therapies have been recognized to cause relapses of HNSCC patients with the advanced disease. Notably, Arf1 has been reported to be critical for cell invasion and metastasis in various cancers [11,18,48,49], including HNSCC [14], as well as the Arf1 inhibitor AMF-26 helped overcome the acquired resistance to EGFR tyrosine kinase inhibitors in non-small cell lung cancer. Arf1 inhibitors are thus particularly interesting to counter metastasis and overcome treatment resistance. We have previously studied Arf1 inhibitors and have shown that LM11 could inhibit cell invasion and suppressed metastasis in breast cancer [18], as well as Exo2 could inhibit cell migration and invasion in prostate cancer [11]. Given all these elements, it is obviously a priority to evaluate the potential effect of our compounds on the invasion and metastasis in HNSCC.

Compared with 2D cell cultures, 3D cell cultures can better mirror in vivo response to drug by taking advantage of recapitulating in vivo cell biology and microenvironmental factors [50]. We have validated the cytotoxic effect of the lead compound **10b** in HNSCC cells growing in a 3D culture system. We obtained the results from 3D assays in an informative, cost-effective, and time-efficient manner however, effects of dosage and dosing frequency on the efficacy and safety are warranted to be determined in preclinical animal models. Further studies using orthotopic and/or patient-derived xenograft (PDX) mice for HNSCC are required to clarify this point.

## 5. Conclusions

Our results constitute a proof of concept that targeting Arf1 may offer a real therapeutic opportunity against HNSCC. New Arf1 targeting γ-dipeptides are here described and they have shown to alter HNSCC cell viability. They could be used in monotherapy or in combination with EGFR targeting drugs. Pairing of EGFR and Arf1 inhibitors may achieve a better clinical outcome, allowing single drug toxicity to reduce and perhaps minimize or delay the induction of drug tolerance.

## Supplementary Materials

The following are available online at https://www.mdpi.com/2073-4409/9/2/286/s1. Scheme S1: Synthesis of β-Keto Ester 1a,b; Scheme S2: Synthesis of ATC 3a,b; Scheme S3: Synthesis of ATC 3c; Scheme S4: Synthesis of ATC 6a’,b’; Scheme S5: Synthesis of ATC 5a’; Scheme S6: Synthesis of ATC 5b’; Scheme S7: Synthesis of ATC 3d; Scheme S8: Synthesis of dimers 8a–d and 7a–c; Scheme S9: Synthesis of dimers 9a–c and 10a–c; Scheme S10: Synthesis of dimer 10d. Table S1: ^1^H NMR chemical shifts for 9a in CD_3_OH at 293 K; Table S2: Coupling constants ^3^J(NH, ^γ^CH) (in Hz) for 9a; Table S3: ^1^H NMR chemical shifts for 9b in CD_3_OH at 293 K; Table S4: Coupling constants ^3^J(NH, ^γ^CH) (in Hz) for 9b; Table S5: Inter-residue NOE correlations in 9b observed in the ROESY spectrum CD_3_OH at 293 K; Table S6: ^1^H NMR chemical shifts for 10a in CD_3_OH at 293 K; Table S7: Coupling constants ^3^J(NH, ^γ^CH) (in Hz) for 10a; Table S8: Inter-residue NOE correlations in 10a observed in the ROESY spectrum CD_3_OH at 293 K; Table S9: ^1^H NMR chemical shifts for 10b in CD_3_OH at 293 K; Table S10: Coupling constants ^3^J(NH, ^γ^CH) (in Hz) for 10b; Table S11: Inter-residue NOE correlations in 10b observed in the ROESY spectrum CD_3_OH at 293 K; Table S12: ^1^H NMR chemical shifts for 10c in CD_3_OH at 293 K; Table S13: Coupling constants ^3^J(NH, ^γ^CH) (in Hz) for 10c; Table S14: Inter-residue NOE correlations in **10c** observed in the ROESY spectrum CD_3_OH at 293 K. Figure S1: ROESY spectra of 9b in CD_3_OH at 293 K; Figure S2: ROESY spectra of 10a in CD_3_OH at 293 K; Figure S3: ROESY spectra of 10b in CD_3_OH at 293 K; Figure S4: ROESY spectra of 3c in CD_3_OH at 293 K.

## References

[1] Bray F., Ferlay J., Soerjomataram I., Siegel R.L., Torre L.A., Jemal A. (2018). Global cancer statistics 2018: GLOBOCAN estimates of incidence and mortality worldwide for 36 cancers in 185 countries. CA Cancer J. Clin..

[2] Siegel R.L., Miller K.D., Jemal A. (2019). Cancer statistics, 2019. CA Cancer J. Clin..

[3] Vigneswaran N., Williams M.D. (2014). Epidemiologic trends in head and neck cancer and aids in diagnosis. Oral Maxillofac. Surg. Clin..

[4] Marur S., Forastiere A.A. (2016). Head and Neck Squamous Cell Carcinoma: Update on Epidemiology, Diagnosis, and Treatment. Mayo Clin. Proc..

[5] Machiels J.-P., Lambrecht M., Hanin F.-X., Duprez T., Gregoire V., Schmitz S., Hamoir M. (2014). Advances in the management of squamous cell carcinoma of the head and neck. F1000Prime Rep..

[6] Magnes T., Egle A., Greil R., Melchardt T. (2017). Update on squamous cell carcinoma of the head and neck: ASCO annual meeting 2017. Mag. Eur. Med. Oncol..

[7] Donaldson J.G., Jackson C.L. (2011). ARF family G proteins and their regulators: Roles in membrane transport, development and disease. Nat. Rev. Mol. Cell Biol..

[8] D’Souza-Schorey C., Chavrier P. (2006). ARF proteins: Roles in membrane traffic and beyond. Nat. Rev. Mol. Cell Biol..

[9] Jackson C.L., Casanova J.E. (2000). Turning on ARF: The Sec7 family of guanine-nucleotide-exchange factors. Trends Cell Biol..

[10] Casalou C., Faustino A., Barral D.C. (2016). Arf proteins in cancer cell migration. Small GTPases.

[11] Lang L., Shay C., Zhao X., Teng Y. (2017). Combined targeting of Arf1 and Ras potentiates anticancer activity for prostate cancer therapeutics. J. Exp. Clin. Cancer Res..

[12] Prieto-Dominguez N., Parnell C., Teng Y. (2019). Drugging the Small GTPase Pathways in Cancer Treatment: Promises and Challenges. Cells.

[13] Vigil D., Cherfils J., Rossman K.L., Der C.J. (2010). Ras superfamily GEFs and GAPs: Validated and tractable targets for cancer therapy?. Nat. Rev. Cancer.

[14] He L., Gao L., Shay C., Lang L., Lv F., Teng Y. (2019). Histone deacetylase inhibitors suppress aggressiveness of head and neck squamous cell carcinoma via histone acetylation-independent blockade of the EGFR-Arf1 axis. J. Exp. Clin. Cancer Res..

[15] Singleton V.L., Bohonos N., Ullstrup A.J. (1958). Decumbin, a new compound from a species of Penicillium. Nature.

[16] Peyroche A., Antonny B., Robineau S., Acker J., Cherfils J., Jackson C.L. (1999). Brefeldin A acts to stabilize an abortive ARF-GDP-Sec7 domain protein complex: Involvement of specific residues of the Sec7 domain. Mol. Cell.

[17] Viaud J., Zeghouf M., Barelli H., Zeeh J.-C., Padilla A., Guibert B., Chardin P., Royer C.A., Cherfils J., Chavanieu A. (2007). Structure-based discovery of an inhibitor of Arf activation by Sec7 domains through targeting of protein-protein complexes. Proc. Natl. Acad. Sci. USA.

[18] Xie X., Tang S.-C., Cai Y., Pi W., Deng L., Wu G., Chavanieu A., Teng Y. (2016). Suppression of breast cancer metastasis through the inactivation of. Oncotarget.

[19] Shao R.G., Shimizu T., Pommier Y. (1996). Brefeldin A is a potent inducer of apoptosis in human cancer cells independently of p53. Exp. Cell Res..

[20] Ohashi Y., Iijima H., Yamaotsu N., Yamazaki K., Sato S., Okamura M., Sugimoto K., Dan S., Hirono S., Yamori T. (2012). AMF-26, a novel inhibitor of the Golgi system, targeting ADP-ribosylation factor 1 (Arf1) with potential for cancer therapy. J. Biol. Chem..

[21] Hafner M., Schmitz A., Grune I., Srivatsan S.G., Paul B., Kolanus W., Quast T., Kremmer E., Bauer I., Famulok M. (2006). Inhibition of cytohesins by SecinH3 leads to hepatic insulin resistance. Nature.

[22] Pan T., Sun J., Hu J., Hu Y., Zhou J., Chen Z., Xu D., Xu W., Zheng S., Zhang S. (2014). Cytohesins/ARNO: The function in colorectal cancer cells. PLoS ONE.

[23] Feng Y., Jadhav A.P., Rodighiero C., Fujinaga Y., Kirchhausen T., Lencer W.I. (2004). Retrograde transport of cholera toxin from the plasma membrane to the endoplasmic reticulum requires the trans-Golgi network but not the Golgi apparatus in Exo2-treated cells. EMBO Rep..

[24] Rouhana J., Hoh F., Estaran S., Henriquet C., Boublik Y., Kerkour A., Trouillard R., Martinez J., Pugniere M., Padilla A. (2013). Fragment-based identification of a locus in the Sec7 domain of Arno for the design of protein-protein interaction inhibitors. J. Med. Chem..

[25] DiNitto J.P., Delprato A., Gabe Lee M.-T., Cronin T.C., Huang S., Guilherme A., Czech M.P., Lambright D.G. (2007). Structural basis and mechanism of autoregulation in 3-phosphoinositide-dependent Grp1 family Arf GTPase exchange factors. Mol. Cell.

[26] UniProtKB—Q99418 (CYH2_HUMAN). https://www.uniprot.org/uniprot/Q99418.

[27] Mathieu L., Legrand B., Deng C., Vezenkov L., Wenger E., Didierjean C., Amblard M., Averlant-Petit M.-C., Masurier N., Lisowski V. (2013). Helical oligomers of thiazole-based amino γ-acids: Synthesis and structural studies. Angew. Chem. Int. Ed..

[28] Mathieu L., Bonnel C., Masurier N., Maillard L.T., Martinez J., Lisowski V. (2015). Cross-Claisen condensation of N-Fmoc-amino acids*—*A short route to heterocyclic γ-amino acids. Eur. J. Org. Chem..

[29] Simon M., Ali L.M.A., El Cheikh K., Aguesseau J., Gary-Bobo M., Garcia M., Morere A., Maillard L.T. (2018). Can Heterocyclic γ-Peptides Provide Polyfunctional Platforms for Synthetic Glycocluster Construction?. Chem. Eur. J..

[30] Chardin P., Paris S., Antonny B., Robineau S., Beraud-Dufour S., Jackson C.L., Chabre M. (1996). A human exchange factor for ARF contains Sec7- and pleckstrin-homology domains. Nature.

[31] Klarlund J.K., Guilherme A., Holik J.J., Virbasius J.V., Chawla A., Czech M.P. (1997). Signaling by phosphoinositide-3,4,5-trisphosphate through proteins containing pleckstrin and Sec7 homology domains. Science.

[32] Vezenkov L.L., Martin V., Bettache N., Simon M., Messerschmitt A., Legrand B., Bantignies J.-L., Subra G., Maynadier M., Bellet V. (2017). Ribbon-like Foldamers for Cellular Uptake and Drug Delivery. ChemBioChem.

[33] Fivush A.M., Willson T.M. (1997). AMEBA: An acid sensitive aldehyde resin for solid phase synthesis. Tetrahedron Lett..

[34] Bonnel C., Legrand B., Bantignies J.-L., Petitjean H., Martinez J., Masurier N., Maillard L.T. (2016). FT-IR and NMR structural markers for thiazole-based γ-peptide foldamers. Org. Biomol. Chem..

[35] Stransky N., Egloff A.M., Tward A.D., Kostic A.D., Cibulskis K., Sivachenko A., Kryukov G.V., Lawrence M.S., Sougnez C., McKenna A. (2011). The mutational landscape of head and neck squamous cell carcinoma. Science.

[36] Alsahafi E., Begg K., Amelio I., Raulf N., Lucarelli P., Sauter T., Tavassoli M. (2019). Clinical update on head and neck cancer: Molecular biology and ongoing challenges. Cell Death Dis..

[37] Ferris R.L., Licitra L., Fayette J., Even C., Blumenschein G., Harrington K.J., Guigay J., Vokes E.E., Saba N.F., Haddad R. (2019). Nivolumab in Patients with Recurrent or Metastatic Squamous Cell Carcinoma of the Head and Neck: Efficacy and Safety in CheckMate 141 by Prior Cetuximab Use. Clin. Cancer Res..

[38] Seiwert T.Y., Burtness B., Mehra R., Weiss J., Berger R., Eder J.P., Heath K., McClanahan T., Lunceford J., Gause C. (2016). Safety and clinical activity of pembrolizumab for treatment of recurrent or metastatic squamous cell carcinoma of the head and neck (KEYNOTE-012): An open-label, multicentre, phase 1b trial. Lancet Oncol..

[39] Troiano G., Caponio V.C.A., Zhurakivska K., Arena C., Pannone G., Mascitti M., Santarelli A., Lo Muzio L. (2019). High PD-L1 expression in the tumour cells did not correlate with poor prognosis of patients suffering for oral squamous cells carcinoma: A meta-analysis of the literature. Cell Prolif..

[40] Lindemann A., Patel A.A., Silver N.L., Tang L., Liu Z., Wang L., Tanaka N., Rao X., Takahashi H., Maduka N.K. (2019). COTI-2, A Novel Thiosemicarbazone Derivative, Exhibits Antitumor Activity in HNSCC through p53-dependent and -independent Mechanisms. Clin. Cancer Res..

[41] Davis J.E., Xie X., Guo J., Huang W., Chu W.-M., Huang S., Teng Y., Wu G. (2016). ARF1 promotes prostate tumorigenesis via targeting oncogenic MAPK signaling. Oncotarget.

[42] Boulay P.-L., Schlienger S., Lewis-Saravalli S., Vitale N., Ferbeyre G., Claing A. (2011). ARF1 controls proliferation of breast cancer cells by regulating the retinoblastoma protein. Oncogene.

[43] Boulay P.-L., Cotton M., Melançon P., Claing A. (2008). ADP-ribosylation factor 1 controls the activation of the phosphatidylinositol 3-kinase pathway to regulate epidermal growth factor-dependent growth and migration of breast cancer cells. J. Biol. Chem..

[44] Anadu N.O., Davisson V.J., Cushman M. (2006). Synthesis and anticancer activity of brefeldin A ester derivatives. J. Med. Chem..

[45] Rouhana J., Padilla A., Estaran S., Bakari S., Delbecq S., Boublik Y., Chopineau J., Pugniere M., Chavanieu A. (2013). Kinetics of interaction between ADP-ribosylation factor-1 (Arf1) and the Sec7 domain of Arno guanine nucleotide exchange factor, modulation by allosteric factors, and the uncompetitive inhibitor brefeldin A. J. Biol. Chem..

[46] Ohashi Y., Okamura M., Katayama R., Fang S., Tsutsui S., Akatsuka A., Shan M., Choi H.-W., Fujita N., Yoshimatsu K. (2018). Targeting the Golgi apparatus to overcome acquired resistance of non-small cell lung cancer cells to EGFR tyrosine kinase inhibitors. Oncotarget.

[47] Haines E., Schlienger S., Claing A. (2015). The small GTPase ADP-Ribosylation Factor 1 mediates the sensitivity of triple negative breast cancer cells to EGFR tyrosine kinase inhibitors. Cancer Biol. Ther..

[48] Schlienger S., Campbell S., Claing A. (2014). ARF1 regulates the Rho/MLC pathway to control EGF-dependent breast cancer cell invasion. Mol. Biol. Cell.

[49] Haines E., Saucier C., Claing A. (2014). The adaptor proteins p66Shc and Grb2 regulate the activation of the GTPases ARF1 and ARF6 in invasive breast cancer cells. J. Biol. Chem..

[50] Lang L., Shay C., Zhao X., Xiong Y., Wang X., Teng Y. (2019). Simultaneously inactivating Src and AKT by saracatinib/capivasertib co-delivery nanoparticles to improve the efficacy of anti-Src therapy in head and neck squamous cell carcinoma. J. Hematol. Oncol..

