# Supplementary material for: Design and Synthesis of Arf1-Targeting γ-Dipeptides as Potential Agents against Head and Neck Squamous Cell Carcinoma
Source: Cells. 2020 Jan 24;9(2):286. doi: 10.3390/cells9020286 (PMC7072570; doi:10.3390/cells9020286)
Supplement: Supplementary file 1 [file cells-09-00286-s001.pdf]

SI: Synthesis of γ-peptidic dimers targeting activation of the GTPase ADP-Ribosylation Factor 1 (ARF1)

Supporting Information

Table des matières

I. General conditions 2

II. Synthetic procedures and characterizations 3

General procedure A : Synthesis of β-Keto Ester 1a,b [8] 3

General procedure B : Synthesis of ATC 3a,b[8] 5

Alternative way to ATC 3c 7

General Procedures C of deprotections 8

General Procedure D : Synthesis of *O*-benzyl esters of 6a' and 6b' 9

Synthesis of *N*-benzyl amide 5a' 11

Alternative way to *N*-benzyl amide 5b' 12

Synthesis of *N*-acetyl-ATC 3d 13

General procedure E : Synthesis of dimers 7a-c and 8a-d 14

General procedure F : Benzoylation of dimers and Synthesis of 9a-c and 10a-c 19

Solid phase synthesis of 10d 22

III. NMR characterization of the folding of the dimers 25

IV. REFERENCES 33

I. General conditions

Commercially available reagents and solvents were used without any further purification. Reactions were monitored by HPLC with an analytical Chromolith Speed Rod RP-C18 185 Pm column (50 X 4.6 mm, 5 µm) using a flow rate of 5.0 ml/min, and gradients from 100/0 to 0/100 eluents A/B over 3 (condition A) or 5 min (condition B), in which eluents A = H_2O / TFA 0.1% and B = CH_3CN / TFA 0.1%. Detection was performed at λ = 214 and 254 nm with a photodiode array detector. The retention times are reported as follows: LC: t_R = [min]. Analytical thin-layer chromatography (TLC) was performed with aluminium-backed silica gel plates coated with a 0.2 mm thickness of silica gel or with aluminium oxide 60 F254, neutral. Column chromatography was performed using 60 Å 40-63 mesh silica gel. The ^{1}H and ^{13}C NMR spectra were recorded at room temperature (RT) in deuterated solvents, using a Bruker AC-300 spectrometer, or a Bruker Avance 600 AVANCE III spectrometer equipped with a 5 mm quadruple-resonance probe (^{1}H, ^{13}C, ^{15}N, ^{31}P). The chemical shifts (δ) are given in parts per million relative to tetramethylsilane (TMS) or by using $CHCl_3$, CD_3OD and DMSO as references (respectively 7.26, 3.31 and 2.5 ppm for ^{1}H spectrum and 77.16, 49.0 and 39.52 ppm for ^{13}C spectrum). Coupling constants (J) are reported in hertz (Hz). Standard abbreviations indicating multiplicity were used as follows: s (singlet), br (broad), d (doublet), dd (doublet doublet), t (triplet), q (quartet), m (multiplet). Infrared (IR) spectra have been recorded on a Perkin Elmer Spectrum One spectrometer. LC–MS spectra (ESI) were recorded with a Quattro micro ESI triple quadrupole mass spectrometer (Micromass, Manchester, UK). The HPLC separations were done using an analytical Chromolith Speed Rod RP-C18 185 Pm column (50 X 4.6 mm, 5 µm) and an Alliance HPLC System (Waters, Milford, USA) at a flow rate of 3.0 ml/min, and a gradient of 100/0 to 0/100 eluents A/B over 2.5 min (solvent A = H_2O / HCOOH 0.1% and solvent B = CH_3CN /HCOOH 0.1%). High-resolution mass spectrometric analyses (HRMS) were performed with a time-of-flight (TOF) mass spectrometer fitted with an electrospray ionization source (ESI). All measurements were performed in the positive-ion mode. Melting points were recorded with a capillary melting point apparatus. All reactions were carried out under an atmosphere of nitrogen unless otherwise indicated.

II. Synthetic procedures and characterizations

ATC **4a,d** was prepared according to the procedure previously reported by our group. [7,8] Intermediates **1a,b** and *N*-Fmoc-ATC-OBn **5a,b** and *N*-Fmoc-ATC-O-dimethylallyl **4c,d**, were previously described.

General procedure A : Synthesis of β-Keto Ester 1a,b [8]

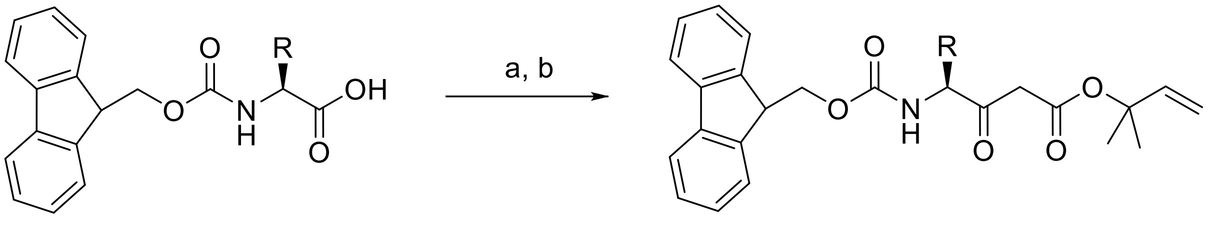

1a,b

Scheme S1 : Synthesis of β-Keto Ester **1a**,**b**. reagents and conditions: a) CDI, DMAP, THF, RT, 1h; b) 1,1-dimethylallyl acetate, LiHMDS, THF, -78°C, 10 min.

Compounds	R	Yields
**1a**	H	60%
**1b**	i-butyl	73%

Imidazolide Formation: In a 250 ml two-neck flask under a nitrogen atmosphere was dissolved the Fmoc-AA-OH (28.59 mmol, 1.0 equiv.) in 100 ml dry THF. Then, CDI (5.10 g, 31.45 mmol, 1.1 equiv.) was added in three portions. A catalytic amount of DMAP (105 mg, 0.857 mmol, 0.03 equiv.) was added 3 min later, and the solution was stirred for 1 h at room temperature (RT).

Enolate Formation: a 500 ml threeneck flask was charged under a nitrogen atmosphere, with LiHMDS (100 ml, 100 mmol, 1 M solution in THF, 3.5 equiv.) followed by dry THF (100 ml). After cooling at –78 °C 1,1-dimethylallyl acetate (14.650 g, 114.3 mmol, 4 equiv.) was added dropwise over 10 min. The solution was stirred at -78 °C for 10 min, then at RT for 10 min, and finally at -78 °C for 20 min.

Condensation: The imidazolide solution was added dropwise to the enolate solution at –78 °C over 5 min. After stirring for 15 min (HPLC/TLC monitoring), the mixture was removed from the cold bath and poured onto a solution of 10 % aqueous citric acid (200 ml) until pH 7. The crude was extracted twice with 100 ml EtOAc. The combined organic layers were washed with water then with 100 ml saturated $NaHCO_3$ solution and brine (3 x 150 ml), dried on $MgSO_4$, filtered, and evaporated under reduced pressure. The crude product was purified by chromatography on silica gel, with a gradient of cyclohexane/ EtOAc from 95/5 vv to 50/50 vv over 10 column volumes.

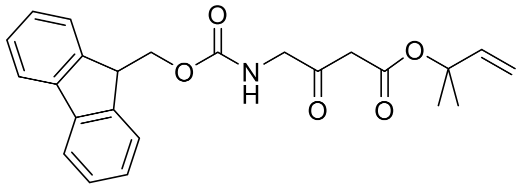

Chemical formula: $C_{24}H_{25}NO_5$
Exact Mass: 407,17
Molecular Weight: 407,46

1,1-Dimethylprop-2-en-1-yl 4-(Fmoc-amino)-3-oxobutanoate (1a) was previously described by Mathieu et al.[7,8]**:**
White solid (7.04 g), yield 60%, m.p. 99–100 °C. ^{1}H NMR (400 MHz, $CDCl_3$): δ = 1.55 (s, 6H), 3.43 (s, 2H), 4.23 (m, 3H), 4.41 (d, *J* = 7.0 Hz, 2H), 5.12 (dd, *J* = 0.6, 10.9 Hz, 1H), 5.21 (d, *J* = 17.5 Hz, 1H), 5.46 (s, 1H), 6.08 (dd, *J* = 10.9, 17.5 Hz, 1H), 7.30 (t, *J* = 7.4 Hz, 2H), 7.40 (t, *J* = 7.4 Hz, 2H), 7.59 (d, *J* = 7.4 Hz, 2H), 7.76 (d, *J* = 7.4 Hz, 2H) ppm. ^{13}C NMR (100 MHz, $CDCl_3$): δ = 26.4 (2 C), 47.3, 47.8, 51.0, 67.3, 83.0, 113.7, 120.1 (2 C), 125.2 (2 C), 127.2 (2 C), 127.9 (2 C), 141.5, 141.7 (2 C), 143.9 (2 C), 156.3, 165.4, 198.5 ppm. LC (conditions A): t_R = 2.19 (major) and 2.40 min (minor; keto–enol equilibrium). FTIR (cm^{-1}): ν_{max} = 3357, 3041, 2979, 1724, 1704, 1449, 1436, 1394, 1306, 1288, 1252, 1109, 1073, 1050, 988, 969, 760, 739. LC–MS (ESI+): m/z (%) = 408.2 (17) $[M + H]^+$, 430.1 (100) $[M + Na]^+$. HRMS (ESI): calcd. for $C_{24}H_{26}NO_5$ $[M + H]^+$ 408.1811; found 408.1811.

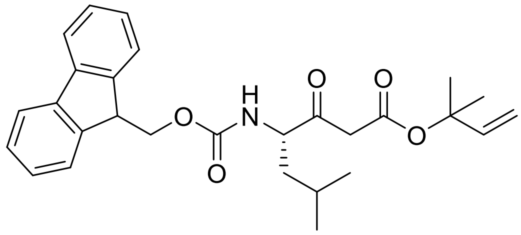

Chemical formula: $C_{28}H_{33}NO_5$
Exact Mass: 463,24
Molecular Weight: 463,57

1,1-Dimethylprop-2-en-1-yl (4S)-4-(Fmoc-amino)-6-methyl-3-oxoheptanoate (1b) was previously described by Mathieu et al.[8]**:**
White solid (8.27 g), yield 73 %, m.p. 117-118 °C. $[\alpha]_D^{20°C}$ = –10.2 (*c* = 1.00, $CHCl_3$). ^{1}H NMR ($CDCl_3$, 400 MHz): δ = 0.95 (t, *J* = 6.2 Hz, 6H), 1.41 (m, 2H), 1.53 (s, 6H), 1.68 (m, 1H), 3.41 (d, *J* = 15.6 Hz, 1H), 3.49 (d, *J* = 15.6 Hz, 1H), 4.22 (t, *J* = 6.6 Hz, 1H), 4.42 (s, 1H), 4.45 (d, *J* = 6.6 Hz, 2H), 5.09 (d, *J* = 10.8 Hz, 1H), 5.19 (d, *J* = 17.4 Hz, 1H), 5.19 (s, 1H), 6.08 (dd, *J* = 10.9, 17.4 Hz, 1H), 7.32 (t, *J* = 7.4 Hz, 2H), 7.40 (t, *J* = 7.4 Hz, 2H), 7.59 (m, 2H), 7.78 (d, *J* = 7.4 Hz, 2H) ppm. ^{13}C NMR ($CDCl_3$, 100 MHz): δ = 21.7, 23.4, 25.0, 26.5 (2 C), 40.3, 47.5 (2 C), 58.8, 67.0, 82.7, 113.5, 120.2 (2 C), 125.1 (2 C), 127.2 (2 C), 127.9 (2 C), 141.5 (2 C), 142.0, 143.9 (2 C), 156.2, 165.7, 202.7 ppm. LC (conditions A): t_R = 2.29 (major) and 2.52 min (minor; keto–enol equilibrium). FTIR (cm^{-1}): ν_{max} = 3375, 3041, 2958, 1739, 1717, 1536, 1452, 1353, 1249, 1237, 1222, 1126, 1098, 1038, 942, 760, 737, 730. LC-MS (ESI+): *m*/*z* (%) = 464.3 (100) $[M + H]^+$, 486.3 (10) $[M + Na]^+$. HRMS (ESI): calcd. for $C_{28}H_{34}NO_5$ $[M + H]^+$ 464.2437; found 464.2416.

General procedure B : Synthesis of ATC 3a,b[8]

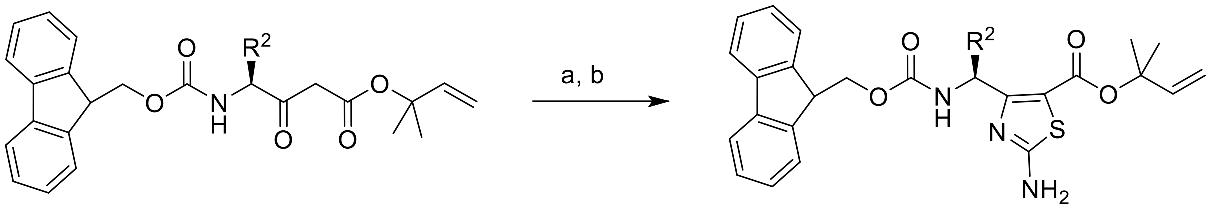

1a,b 3a,b

Scheme S2 : Synthesis of ATC **3a**,**b** . reagents and conditions: a) NBS, $Mg(ClO_4)_2$, ACN, -45°C, 10 min; b) thiourea, EtOH, 40°C, 2h;

Compounds	R^2	Yields
**3a**	H	83%
**3b**	i-butyl	85%

Synthesis of bromo-β-keto esters:

To a solution of the β-keto ester **1a,b** (2.87 mmol, 1.0 equiv.) in CH_3CN (20 ml) was added magnesium perchlorate (210 mg, 0.95 mmol, 0.3 equiv.). The solution was stirred at - 45 °C for 10 min. A solution of NBS (537 mg, 3.01 mmol, 1.1 equiv.) in CH_3CN (15 ml) was then added dropwise over 5 min. The reaction was complete after few minutes stirring at - 45 °C (HPLC monitoring). The mixture was diluted with Et_2O (40 ml) and washed twice with 40 ml water and with brine (3 x 20 ml). The organic layer was dried with $MgSO_4$ and filtered. The solvent was removed under reduced pressure to give the crude product, which was used in the next step without further purification.

Hantzsch cyclization: To a solution of α-monobrominated β-keto ester (2.51 mmol, 1.0 equiv.) in absolute EtOH (50 ml) was added a solution of the thiourea (0.226 g, 3.01 mmol, 1.2 equiv.) dissolved in absolute EtOH (10 ml). The solution was heated for 2 h at 40 °C until completion of the reaction (HPLC and TLC monitoring). The solvent was evaporated under reduced pressure at a temperature lower than 30°C in order to avoid any degradation. The yellowish solid was partitioned between EtOAc (25 ml) and water (25 ml). The organic layer was washed with water (3 x 20 ml) and brine (1 X 20 ml). The combined organic layers were dried with $MgSO_4$ and filtered, and the solvent was evaporated under reduced pressure to yield the crude product. Purification chromatography on a silica gel column, with a gradient of cyclohexane/ EtOAc from 95/5 vv to 50/50 vv afforded ATC **3a,b**.

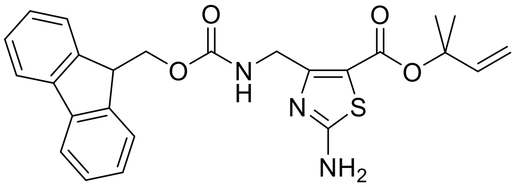

Chemical formula: $C_{25}H_{25}N_3O_4S$
Exact Mass: 463,16
Molecula Weight: 463,55

***N*-Fmoc-{H, NH$_2$}-ATC-*O*-diméthylallyl:** **1,1-Dimethylprop-2-en-1-yl 2-amino-4-[(Fmoc-amino)methyl]-1,3- thiazole-5-carboxylate 3a:**
White solid, yield 83%, m.p. 89-92.2 °C. ^{1}H NMR ($CDCl_3$, 400 MHz): δ = 1.62 (s, 6H), 4.22 (t, J = 7.1 Hz, 1H), 4.38 (d, J = 7.1 Hz, 2H), 4.53 (d, J = 6.1 Hz, 2H), 5.15 (d, J = 10.8 Hz, 1H), 5.23 (d, J = 17.5 Hz, 1H), 5.92 (s, 1H), 6.16 (dd, J = 10.8, 17.5 Hz, 1H), 6.25 (br., 2H), 7.29 (t, J = 7.4 Hz, 2H), 7.38 (t, J = 7.4 Hz, 2H), 7.60 (d, J = 7.4 Hz, 2H), 7.75 (d, J = 7.4 Hz, 2H) ppm. ^{13}C NMR ($CDCl_3$, 100 MHz): δ = 26.7 (2C), 39.9, 47.2, 67.0, 82.3, 113.0, 120.0 (2C), 125.2 (3C), 127.1 (2C), 127.7 (3C), 141.3, 142.4 (2C), 143.9 (2C), 157.3, 161.2, 170.8 ppm. LC (conditions A): t_R = 2.80 min. FTIR (cm^{-1}): ν_{max} = 3319, 3193, 3069, 1684, 1621,1496, 1451, 1280, 1264, 1232, 1137, 1082, 1046, 921, 827, 732. LC-MS (ESI+): m/z (%) = 464.1 (42) [M + H]$^+$, 486.0 (18) [M + Na]$^+$. HRMS (ESI): calcd. for $C_{25}H_{26}N_3O_4S$ [M + H]$^+$ 464.1644; found 464.1646.

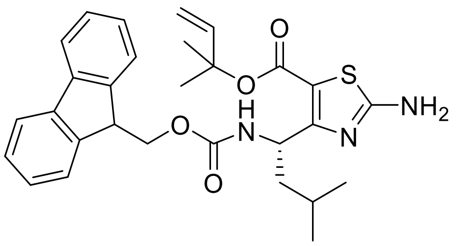

Chemical formula: $C_{29}H_{33}N_3O_4S$
Exact Mass: 519,22
Molecular Weight: 519,66

***N*-Fmoc-{iBu, NH$_2$}-ATC-*O*-diméthylallyl:** **1,1-Dimethylprop-2-en-1-yl 2-Amino-4-[(1*S*)-1-(Fmoc-amino)-3-methylbutyl]-1,3-thiazole-5-carboxylate (3b)** was previously described by Mathieu et al.[8]
White solid, yield 85% , m.p. 113-114 °C. $[\alpha]_D^{20°C}$ = –16.0 (c = 1.00, $CHCl_3$). ^{1}H NMR ($CDCl_3$, 400 MHz): δ = 0.95 (m, 6H), 1.53 (m, 2H), 1.61 (s, 3H), 1.62 (s, 3H), 1.73 (m, 1H), 4.21 (m, 1H), 4.32 (dd, J = 10.3, 7.0 Hz, 1H), 4.40 (dd, J = 10.3, 7.0 Hz, 1H), 5.12 (d, J = 10.9 Hz, 1H), 5.23 (d, J = 17.6 Hz, 1H), 5.42 (m, 1H), 5.82 (br., 2H), 6.04 (m, 1H), 6.17 (dd, J = 17.6, 10.9 Hz, 1H), 7.29 (t, J = 7.4 Hz, 2H), 7.38 (t, J = 7.4 Hz, 2H), 7.59 (d, J = 7.4 Hz, 2H), 7.75 (d, J = 7.4 Hz, 2H) ppm. ^{13}C NMR ($CDCl_3$, 100 MHz): δ = 22.3, 23.3, 25.1, 26.7, 27.0, 44.4, 47.4, 49.2, 66.7, 82.5, 113.1, 120.0 (2C), 125.3 (3C), 127.1 (2C), 127.7 (3C), 141.4 (2C), 142.5, 144.1 (2C), 156.1, 161.6, 170.1 ppm. LC (conditions A): t_R = 2.23 min. LC-MS (ESI+): m/z (%) = 520.2 (100) [M + H]$^+$, 542.2 (10) [M + Na]$^+$. HRMS (ESI): calcd. for $C_{29}H_{34}N_3O_4S$ [M + H]$^+$ 520.2270; found 520.2274. FTIR (cm^{-1}): ν_{max} = 3313, 2955, 1695, 1615, 1496, 1450, 1316, 1257, 1075, 1043, 923, 827, 758, 738.

Alternative way to ATC 3c

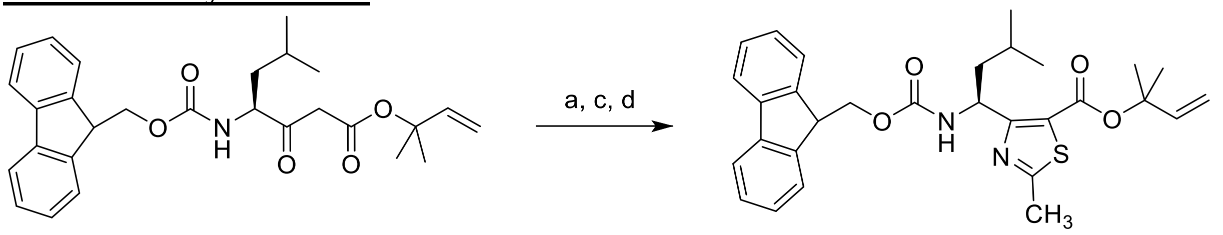

1b 3c

Scheme S3 : Synthesis of ATC **3c** . reagents and conditions: a) NBS, $Mg(ClO_4)_2$, ACN, -45°C, 10 min; c) thioacetamide, $KHCO_3$, DME, RT, 2h; d) DMAP, DIEA, TFAA, DCM, 0°C, 3h.

3c was obtained by a slight modification of our previously reported procedure for *N*-Fmoc protected ATC starting from **1b** and thioacetamide.[8]

To a solution of α-monobrominated β-keto ester **2b** (1.17 g, 2.34 mmol, 1.0 equiv.) in dimethoxyethane (50 ml) were added thioacetamide (0.193 g, 2.57 mmol, 1.1 equiv.) and $KHCO_3$ (1.87 g, 0.0187 mol, 8 equiv.). The mixture was stirred for 2 hours at RT. Afterward, the medium was diluted with EtOAc (60 ml) and washed with water (2 x 40 ml) and with brine (3 x 40 ml). The organic layer was dried over $MgSO_4$, filtered and the solvent was removed under reduced pressure to give a thiazoline intermediate as mixture of two diastereoisomers. HPLC: t_R = 3.50 min (conditions B)

The thiazoline (2.34 mmol) was solubilized in anhydrous DCM (30 ml). The solution was cooled to 0°C, than DMAP (0.286 g, 2.34 mmol, 1.0 equiv.) and *N*,*N*-diisopropylethylamine (DIEA, 3.46 ml, 0.021 mol, 9.0 equiv.) were added in one portion. A solution of TFAA (1.12 ml, 8.07 mmol, 3.45 equiv.) in DCM (5 ml) was added dropwise over 5 minutes at 0°C. After stirring 3 hours at 0°C, the reaction was stopped by adding water (40 ml). The organic layer was washed with water (2 x 40 ml) and with brine (2 x 40 ml). The organic phase was then dried over $MgSO_4$, filtered and the solvent was removed under reduced pressure to give the crude product. Purification by chromatography on a silica gel column using a gradient of cyclohexane/EtOAc from 95/5 vv to 70/30 vv afforded the ATC **3c** (640 mg, 58% yield).

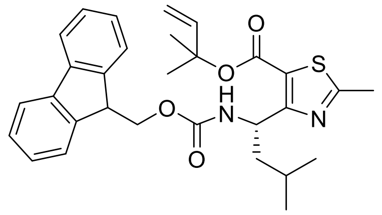

Chemical formula: $C_{30}H_{34}N_2O_4S$
Exact Mass: 518,22
Molecular Weight: 518,67

***N*-Fmoc-{iBu, CH3}-ATC-*O*-diméthylallyl:** **1,1-Dimethylprop-2-en-1-yl 4-[(1*S*)-1-(Fmoc-amino)-3-methylbutyl]-2-methyl-1,3-thiazole-5-carboxylate (3c)** was previously described by Mathieu et al.[8]**:**

White solid, m.p. 98-99°C, $[\alpha]_D^{20°C}$ = -0.6° (c 1.00, $CHCl_3$). ^{1}H NMR ($CDCl_3$, 400 MHz) δ = 0.95 (d, *J* = 6.4 Hz, 3H), 0.99 (d, *J* = 6.4 Hz, 3H), 1.56 (m, 2H), 1.66 (s, 6H), 1.75 (m, 1H), 2.67 (s, 3H), 4.21 (t, *J* = 7.4 Hz, 1H), 4.35 (m, 2H), 5.14 (d, *J* = 10.5 Hz, 1H), 5.26 (d, *J* = 17.5 Hz, 1H), 5.70 (m, 1H), 5.83 (m, 1H), 6.19 (dd, *J* = 17.5, 10.5 Hz, 1H), 7.29 (m, 2H), 7.39 (t, *J* = 7.4 Hz, 2H), 7.60 (m, 2H), 7.75 (d, *J* = 7.4 Hz, 2H) ppm. ^{13}C NMR ($CDCl_3$, 100 MHz) δ = 19.5, 22.3, 23.3, 25.1, 26.7, 26.8, 45.1, 47.4, 48.8, 66.7, 83.3, 113.5, 120.0 (2C), 123.8, 125.3 (2C), 127.1 (2C), 127.7 (2C), 141.4 (2C), 142.1, 144.1, 144.2, 155.9, 160.4, 162.2, 169.8 ppm. LC (condition A): t_R = 2.45 min. FT-IR (cm^{-1}): ν_{max} 3326, 2956, 1699, 1507, 1450, 1327, 1278, 1251, 1191, 1082, 1044, 923, 830, 759, 736. LC-MS: (ESI+): m/z 519.3 ([M+H]$^+$) 100%, 541.3 ([M+Na]$^+$) 10%. HRMS (ESI) calcd for $C_{30}H_{35}N_2O_4S^+$: 519.2305, found 520.2318.

General Procedures C of deprotections

C1: General procedure for dimethyl allyl removal [7,8]
$Pd(PPh_3)_4$ (0.0245 g, 0.0212 mmol, 0.03 equiv.) was dispersed into a solution of *O*-dimethyl allyl ester (0.706 mmol, 1.0 equiv.) and $PhSiH_3$ (0.261 ml, 2.12 mmol, 3 equiv.) in 30 ml of anhydrous THF under nitrogen atmosphere. After stirring for 3 hours (HPLC monitoring), the reaction was stopped by adding a mix of diethyl ether (10 ml) and ethyl acetate (2ml). A white solid formed in the medium was collected by filtration and was used without any further purification.

C2: General procedure for Fmoc removal
Fmoc derivative (1.23 mmol, 1.0 equiv.) was solubilized in 25 ml of diethylamine / DMF (1:9 vv) and stirred for 30 min at RT. The solvent was evaporated and the crude was used without any further purification.

General Procedure D : Synthesis of *O*-benzyl esters of 6a' and 6b'

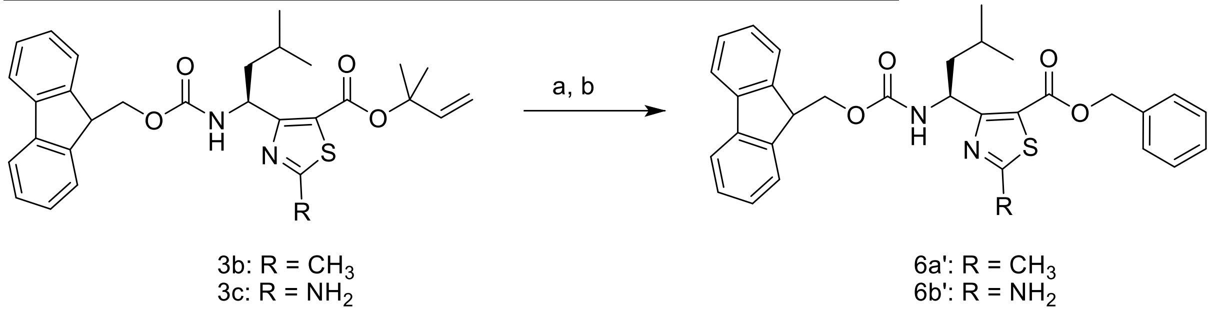

Scheme S4 : Synthesis of ATC **6a',b'**. reagents and conditions: a) $Pd(PPh_3)_4$, $PhSiH_3$, THF, RT, 3h; c) Cs_2CO_3, MeOH, RT, 10 min.; d) BnBr, DMF, RT, 3h.

Compounds	R	Yields
**6a'**	CH_3	73%
**6b'**	NH_2	84%

3b,c were deprotected following the general procedure C1 to afford the free acid **4b,c** which was used without any further purification. The acidic intermediate **4b,c** (1.703 g, 3.772 mmol, 1 equiv.) was converted to its cesium salt by dissolving in MeOH (50 ml) and addition of a solution of 20 % mv cesium carbonate in water (3.0 ml, 0.615 g, 1.886 mmol, 0.5 equiv.). The solvent was removed under reduced pressure then the product was dissolved in DMF (20 ml). Benzyl bromide (0.496 ml, 4.149 mmol, 1.1 equiv.) was added to the mixture. The solution was stirred 1 hour at RT and the DMF was evaporated under reduced pressure. The solid residue was partitioned between AcOEt (120 ml) and water (80 ml). The organic layer was washed with water (1 x 80 ml) and brine (1 x 80 ml) then dried with $MgSO_4$ and filtered. The solvent was evaporated under reduced pressure to yield the crude product. Purification by chromatography on a silica gel column with a gradient of cyclohexane/ EtOAc from 95/5 to 50/50 afforded **6a',b'**.

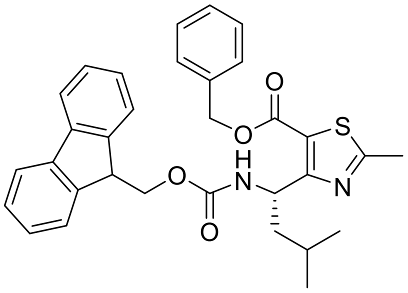

Chemical formula: $C_{32}H_{32}N_2O_4S$
Exact Mass: 540,21
Molecular Weight: 540,67

_N_-Fmoc-{iBu, CH_3}-ATC-_O_-benzyl: Benzyl 4-[(1S)-1-(N-Fmoc)-amino-3-methylbutyl]-2-methyl-1,3-thiazole-5-carboxylate 6a' was previously described by Mathieu et al.[7]**:**
Pale yellow solid, yield: 73%, m.p. 80.0-81.2 °C, $[\alpha]_D^{20°C}$ = +3.6 (*c* =1.00, $CHCl_3$). ^{1}H NMR ($CDCl_3$, 400 MHz) δ = 0.94 (d, *J* = 8.0 Hz, 3H), 0.96 (d, *J* = 8.0 Hz, 3H), 1.54 (m, 1H), 1.66-1.72 (m, 2H), 2.69 (s, 3H), 4.22 (t, *J* = 7.0 Hz, 1H), 4.36 (m, 2H), 5.34 (s, 2H), 5.77 (s, 2H), 7.27-7.44 (m, 9H), 7.60 (t, *J* = 7.0 Hz, 2H), 7.76 (d, *J* = 7.0 Hz, 2H) ppm. ^{13}C NMR ($CDCl_3$, 100 MHz) δ = 19.5, 21.9, 23.4, 25.1, 45.2, 47.4, 48.9, 66.8, 67.3, 120.0 (2C), 121.7, 125.3 (2C), 127.1 (2C), 127.7 (2C), 128.5 (2C), 128.6, 128.8 (2C), 135.4, 141.4 (2C), 144.1, 144.2, 156.0, 161.2, 163.6, 170.4 ppm. LC t_R = 2.13 min (conditions A). FT-IR (cm^{-1}): ν_{max} 3320, 2955, 1695, 1614, 1496, 1450, 1371, 1253, 1175, 1042, 758, 739. LC-MS (ESI+): *m/z* (%) = 541.1 (100) [M+H]]$^+$, 563.1 (30) [M+Na]$^+$. HRMS (ESI): calcd for $C_{32}H_{33}N_2O_4S$: [M+H]$^+$ 541.2161, found 541.2164.

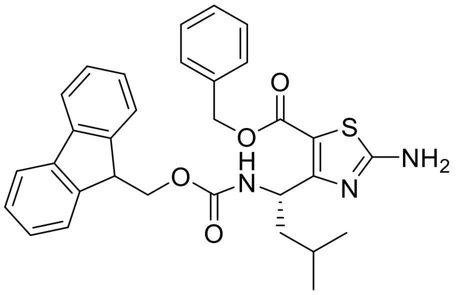

Chemical formula: $C_{31}H_{31}N_3O_4S$
Exact Mass: 541,20
Molecular Weight: 541,66

_N_-Fmoc-{iBu, NH_2}-ATC-_O_-benzyl: Benzyl 2-amino-4-[(1S)-1-(N-Fmoc)-amino-3-methylbutyl]-1,3-thiazole-5-carboxylate 6b' was previously described by Mathieu et al.[7]**:**
Pale yellow solid, yield 84% (1.72 g), m.p. 80.0-81.2 °C, $[\alpha]_D^{20°C}$ = -6.4 (*c* = 1.00, $CHCl_3$). ^{1}H NMR ($CDCl_3$, 400 MHz) δ = 0.91 (d, *J* = 7.0 Hz, 6H), 1.50 (m, 1H), 1.64-1.70 (m, 2H), 2.1 (s, 2H,), 4.22 (t, J = 7.0 Hz, 1H), 4.36 (m, 2H), 5.28 (s, 2H), 5.53 (m, 1H), 6.01 (d, *J* = 7.5 Hz, 1H), 7.27-7.43 (m, 9H), 7.60 (t, *J* = 7.0 Hz, 2H), 7.74 (d, *J* = 7.0 Hz, 2H) ppm. ^{13}C NMR ($CDCl_3$, 100 MHz) δ = 21.8, 23.3, 25.0, 44.3, 47.4, 48.9, 66.9, 67.0, 120.1 (2C), 121.8, 125.4 (2C), 127.2 (2C), 127.8 (2C), 128.4 (2C), 128.5, 128.7 (2C), 135.7, 141.4 (2C), 144.1, 144.2, 156.2, 161.4, 163.4, 170.1 ppm. LC t_R = 2.11 min (conditions A). FT-IR (cm^{-1}): ν_{max} 3320, 2955, 1695, 1614, 1496, 1450, 1371, 1253, 1175, 1042, 758, 739.LC-MS (ESI+): *m/z* (%) = 542.1 (100) [M+H]$^+$, 564.2 (10) [M+Na]$^+$. HRMS (ESI): calcd for $C_{31}H_{32}N_3O_4S$: [M+H]$^+$ 542.2126, found 542.2114.

<u>Synthesis of *N*-benzyl amide 5a'</u>

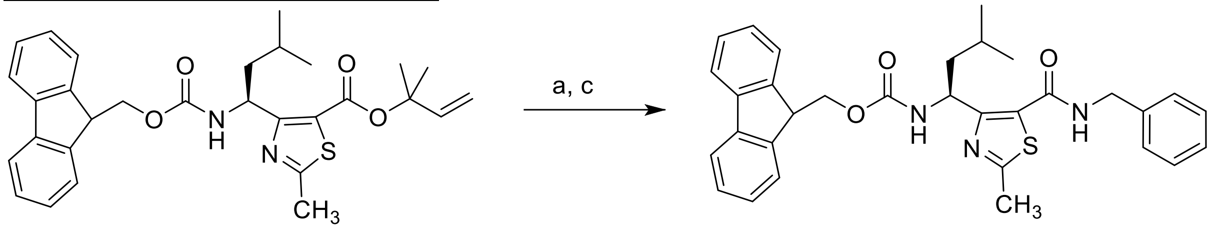

3b 5a'

Scheme S5 : Synthesis of ATC **5a'** . reagents and conditions: a) $Pd(PPh_3)_4$, $PhSiH_3$, THF, RT, 3h; c) IBCF, DIEA, benzylamine, THF, 0°C to RT, 2h.

3b was deprotected following the general procedure C1 to afford the free acid **4b** which was used without any further purification. The acidic intermediate **4b** (0.556 g, 1.23 mmol, 1 equiv.) was dissolved in anhydrous THF (30 ml). The solution was cooled to 0°C and isobutylchloroformate (IBCF, 0.177 ml, 1.36 mmol, 1.2 equiv.) and DIEA (0.234 ml, 1.36 mmol, 1.2 equiv.) were added in one portion. The reaction was stirred for 10 minutes at 0°C then 30 minutes at RT then benzylamine (0.270 ml, 2.46 mmol, 2 equiv.) was added and the reaction was stirred at RT for 1 hour. THF was evaporated under reduced pressure and the crude was dissolved in DCM (60 ml) and washed with a saturated solution of $NaHCO_3$ (2 x 30 ml) and brine (2 x 30 ml). The organic layer was dried with $MgSO_4$ and filtered, and the solvents were evaporated under reduced pressure. The crude was purified by chromatography on a silica gel column with a gradient of cyclohexane/ EtOAc from 95/5 to 70/30 afforded **5a'** with a yield of 95% (0.64 g).

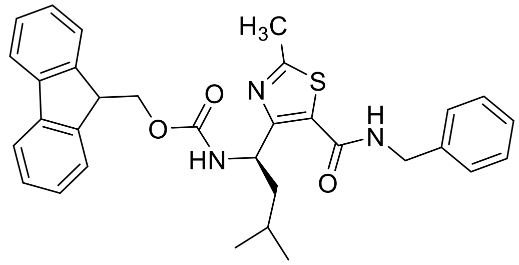

Chemical Formula: $C_{32}H_{33}N_3O_3S$
Exact Mass: 539.22
Molecular Weight: 539.69

<u>*N*-Fmoc-{iBu, CH_3}-ATC-*NH*-benzyl:</u> *N*-Benzyl 4-[(1S)-1-(N-Fmoc)-amino-3-methylbutyl]-2-methyl-1,3-thiazole-5-carboxamide 5a'

Solid, m.p. 76.5-78.5 °C, $[\alpha]_D^{20°C}$ = +0.16 (*c* = 1.00, CH_3OH). ^1H NMR ($CDCl_3$, 300 MHz) δ = 0.85 (d, *J* = 6.7 Hz, 6H), 1.43 (m, 1H), 1.78 (m, 2H), 2.66 (s, 3H), 4.10 (t, *J* = 7.3 Hz, 1H), 4.26 – 4.38 (m, 2H), 4.58 (ABx, *J* = 6.0, 14.6 Hz, 1H), 4.78 (ABx, *J* = 6.0, 14.6 Hz, 1H), 5.05 (q, *J* = 7.8 Hz, 1H), 5.96 (d, *J* = 7.8 Hz, 1H), 7.26 – 7.46 (m, 11H), 7.75, (dd, *J* = 2.7, 7.5 Hz, 2H), 9.35 (t, *J* = 6.0 Hz, 1H) ppm. ^{13}C NMR ($CDCl_3$, 75 MHz) δ = 19.4, 22.4, 22.6, 25.0, 43.6, 44.3, 47.4, 48.7, 67.4, 120.2 (2C), 125.2 (2C), 127.3 (2C), 127.5 (2C), 128.0 (2C), 128.4, 128.8 (2C), 131.4, 138.7, 141.5 (2C), 143.8, 143.9, 153.9, 157.5, 161.4, 168.5 ppm. FT-IR (cm^{-1}): ν_{max} 3257, 3063, 2956, 1689, 1647, 1542, 1450, 1283, 1256, 738. LC t_R = 3.57 min (conditions B). LC-MS (ESI+): *m/z* (%) = 539.9 (100) $[M+H]^+$, 562.1 (20) $[M+Na]^+$. HRMS (ESI): calcd for $C_{32}H_{34}N_3O_3S$: $[M+H]^+$ 540.2321, found 540.2319.

Alternative way to *N*-benzyl amide 5b’

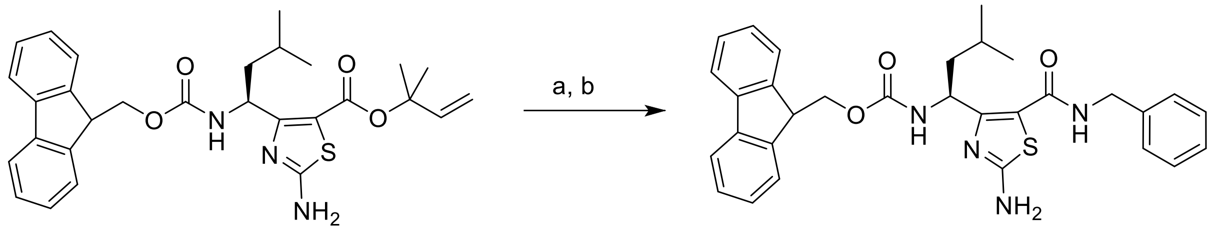

3c 5b'

Scheme S6 : Synthesis of ATC **5b’** . reagents and conditions: a) $Pd(PPh_3)_4$, $PhSiH_3$, THF, RT, 3h; b) EDC.HCl, NMM, DMAP, benzylamine, THF, 0°C to RT, 15h.

5b’ was obtained starting from **4c** and benzylamine by a slight modification of the previous procedure. Indeed the use of IBCF did not lead to the targeted compound **5b’**. We opted then for the use of EDC coupling agent.

3c was deprotected following the general procedure C1 to afford the free acid **4c** which was used without any further purification. The acidic intermediate **4c** (0.166 g, 0.37 mmol, 1.0 equiv.) was dissolved in anhydrous THF (30 ml). EDC.HCl (0.141 g, 0.735 mmol, 2 equiv.), DMAP (0.004 g, 0.0368 mmol, 0.1 equiv.), NMM (0.080 ml, 0.735 mmol, 2 equiv.) were added to the solution. The mixture was stirred at RT for 1 hour then benzylamine (0.080 ml, 0.735 mmol, 2 equiv.) was added in one portion. After stirring overnight at RT under nitrogen atmosphere, THF was evaporated under reduced pressure. The crude was dissolved in DCM (20 ml) and the organic phase was washed with a saturated aqueous solution of $NaHCO_3$ (2 x 30 ml) and brine (2 x 30 ml). The organic layer was dried with $MgSO_4$ and filtered, and the solvent was evaporated under reduced pressure. The crude was purified by chromatography on a silica gel column with a gradient of EtOAc / MeOH from 100 / 0 to 90 / 10 afforded *N*-Fmoc-{iBu, NH_2}-ATC-NHBn **5b’** with a yield of 20% (40 mg).

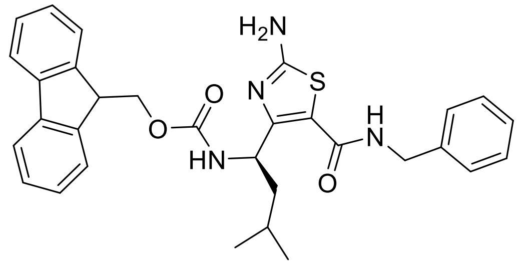

Chemical Formula: $C_{31}H_{32}N_4O_3S$
Exact Mass: 540.22
Molecular Weight: 540.68

<u>*N*-Fmoc-{iBu, NH_2}-ATC-*NH*-benzyl:</u> *N*-Benzyl 2-amino-4-[(1S)-1-(N-Fmoc)-amino-3-methylbutyl]-1,3-thiazole-5-carboxamide 5b’:

Solid. ^1H NMR (CD_3OD, 300 MHz) δ = 0.79 (d, J = 7.0 Hz, 6H), 1.37 (m, 1H), 1.61 (t, J = 7.0 Hz, 2H), 4.13 (t, J = 6.6 Hz, 1H), 4.37 (d, J = 6.6 Hz, 2H), 4.40 (d, J = 14.7 Hz, 1H), 4.63 (d, J = 14.7Hz, 1H), 4.87 (m, 1H), 7.16 - 7.42 (m, 9H), 7.54 (m, 2H), 7.75 (d, J = 7.5 Hz, 2H) ppm. ^{13}C NMR (CD_3OD, 75 MHz) δ = 22.6, 22.9, 25.9, 43.8, 44.5, 48.5 (2C), 67.7, 118.3, 120.8 (2C), 126.1 (2C), 128.1 (2C), 128.2 (2C), 128.7 (2C), 128.9, 129.5 (2C), 139.9, 142.5 (2C), 145.0, 145.2, 154.8, 159.0, 164.0, 171.7 ppm. LC t_R = 3.24 min (conditions B). LC-MS (ESI+): m/z (%) = 541.2 (100) $[M + H]^+$.

Synthesis of *N*-acetyl-ATC 3d

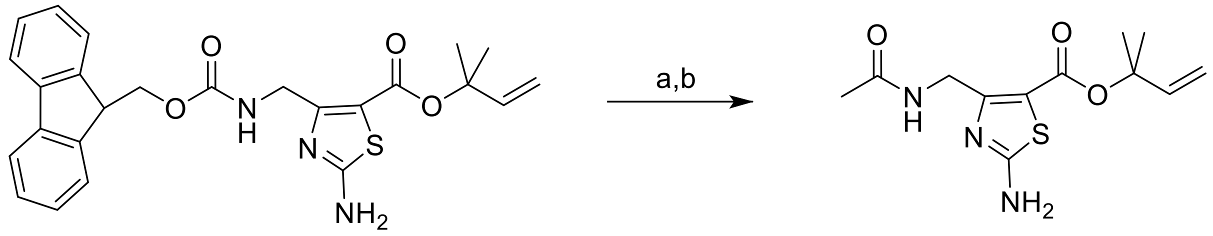

3a 3d

Scheme S7 : Synthesis of ATC **3d** . reagents and conditions: a) diethylamine / DMF (1:9 vv), RT, 30 min; b) Ac_2O, DMF RT, 2h.

3a was deprotected following the general procedure C2 to afford the free amine, which was dissolved in DMF (25 ml). Acetic anhydride (0.081 ml, 0.864 mmol, 1 equiv.) was added to the solution. After 30 minutes stirring at RT, the DMF was removed under reduced pressure. Purification by silica gel column chromatography, with a gradient of EtOAc/MeOH from 100/0 to 95/5 afforded the desired compound **3d** with a yield of 82% (200 mg).

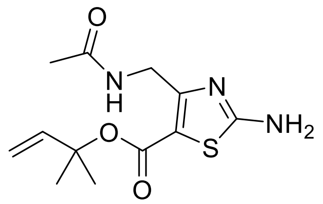

Chemical formula: $C_{12}H_{17}N_3O_3S$
Exact Mass: 283,10
Molecular Weight: 283,35

<u>*N*-acetyl-{H, NH_2}-ATC-*O*-diméthylallyl:</u> 1,1-Dimethylprop-2-en-1-yl 2-amino-4-[(acetylamino)methyl]-1,3- thiazole-5-carboxylate 3d:
White powder. ^{1}H NMR ($CDCl_3$, 300 MHz) δ = 1.60 (s, 6H), 1.99 (s, 3H), 4.56 (d, *J* = 6 Hz, 2H), 5.13 (d, *J* = 10.9 Hz, 1H), 5.22 (d, *J* = 17.5 Hz, 1H), 6.14 (dd, *J* = 10.9, 17.5 Hz, 1H), 6.26 (br., 2H), 6.49 (br., 1H) ppm. ^{13}C NMR ($CDCl_3$, 75 MHz) δ = 23.7 (2C), 27.0, 38.8, 82.6, 113.3, 142.7, 157.7, 159.9, 161.7, 170.1, 170.6 ppm. FT-IR (cm^{-1}): ν_{max} 3363, 2982, 2935, 1715, 1515, 1450, 1247, 1122, 740. LC t_R = 1.62 min (conditions B). LC-MS (ESI+): *m/z* (%) = 284.3 (13) $[M+H]^+$, 306.2 (8) $[M+Na]^+$. HRMS (ESI): calcd for $C_{12}H_{18}N_3O_3S$: $[M+H]^+$ 284.1069, found 284.1066.

General procedure E : Synthesis of dimers 7a-c and 8a-d

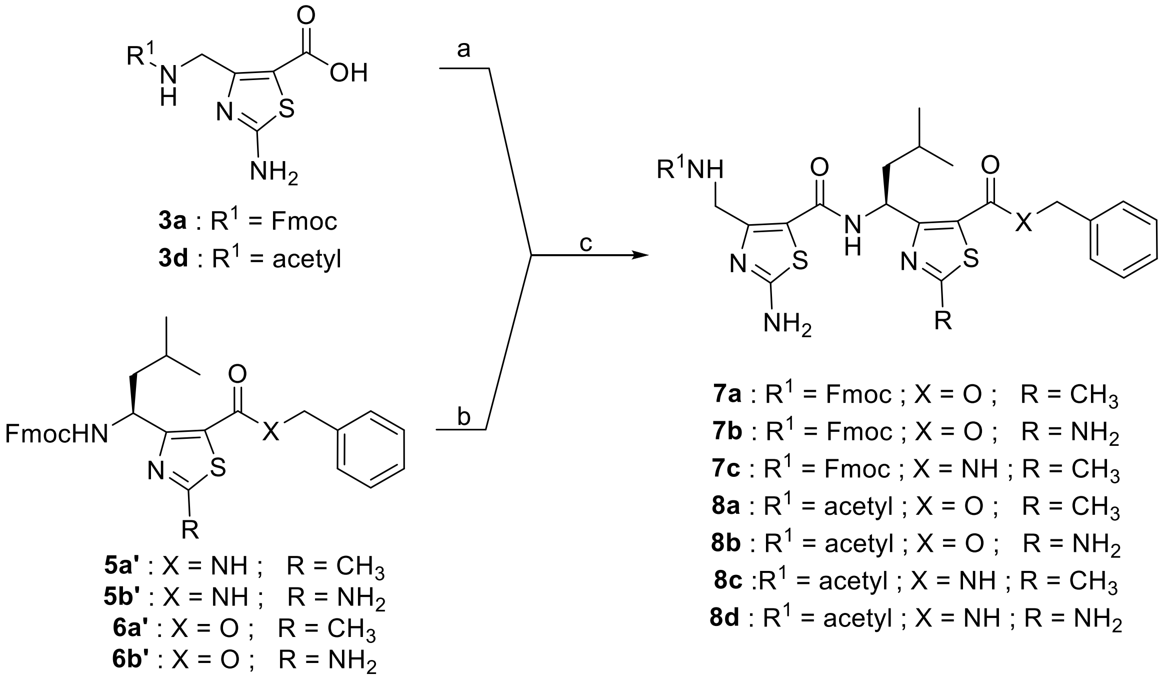

Scheme S8 : Synthesis of dimers **8a-d** and **7a-c** . reagents and conditions: a) $Pd(PPh_3)_4$, $PhSiH_3$, THF, RT, 3h; b) diethylamine / DMF (1:9 vv), RT, 30 min; c) EDC.HCl, NMM, HOBt, DMF, 0°C to RT, 3h.

Compounds	X	R	R^1	Yields
**7a**	-O-	CH_3	Fmoc	48%
**7b**	-O-	NH_2	Fmoc	88%
**7c**	-NH-	CH_3	Fmoc	60%
**8a**	-O-	CH_3	Ac	65%
**8b**	-O-	NH_2	Ac	18%
**8c**	-NH-	CH_3	Ac	61%
**8d**	-NH-	NH_2	Ac	24%

After acidic deprotection of **3a,d** (general procedure C1), the free acid **4a,d** (1.0 equiv., 0.37 mmol) was dissolved in 10 ml anhydrous DMF at 0°C, and EDC.HCl (1.2 equiv. , 0.44 mmol), NMM (1.2 equiv., 0.44 mmol) and HOBt (1.2 equiv., 0.44 mmol) were added. After Fmoc removal for **5a',b'** or **6a',b'** (general procedure C2), the free amine (1.0 equiv., 0.37 mmol) was added to the mixture and was stirred 30 min at 0°C then 4 h at RT until completion (HPLC monitoring). If free amine remains in the medium, free acid and coupling reagents (1.2 equiv.) were added. The solvent was removed under *vacuum*. The crude was purified by chromatography on silica gel (cyclohexane/AcOEt gradient from 70/00 to 0/100) for the compounds **7a-c** or alumina (dichloromethane/ethanol gradient from 99/1 to 90/10) for the compounds **8a-c** or reverse phase (acetonitrile/water gradient gradient from 35/65 to 65/35) for **8d**.

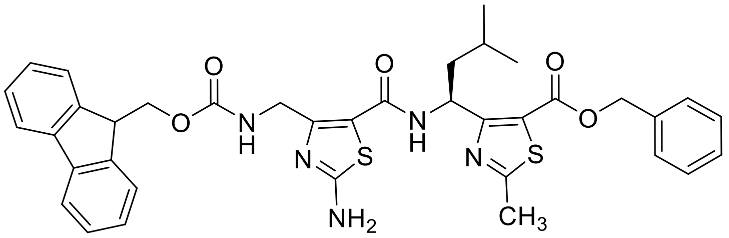

Chemical Formula: $C_{37}H_{37}N_5O_5S_2$
Exact Mass: 695,22
Molecular Weight: 695,85

Benzyl 4-(1-{[2-amino-4-({Fmoc-amino}methyl)-1,3-thiazol-5-yl]formamido}-3-methylbutyl)-2-methyl-1,3-thiazole-5-carboxylate 7a

White powder, yield 48% (122 mg). ^{1}H NMR ($CDCl_3$, 300 MHz) δ = 0.92 (d, *J* = 6.7 Hz, 3H), 0.95 (d, *J* = 6.7 Hz, 3H), 1.51 – 1.64 (m, 1H), 1.64 – 1.79 (m, 1H), 1.79 – 1.92 (m, 1H), 2.57 (s, 3H), 4.10 – 4.24 (m, 2H), 4.35 (d, *J* = 6.8 Hz, 2H), 4.52 (dd, *J* = 15.1 – 6.6 Hz, 1H), 5.31 (s, 2H), 6.02 (m, 1H), 6.24 (br., 2H), 6.73 (br., 1H), 7.19 – 7.42 (m, 9H), 7.50 (m, 2H), 7.69 (d, *J* = 7.4 Hz, 2H), 8.16 (br., 1H) ppm. ^{13}C NMR ($CDCl_3$, 75 MHz) δ = 19.8, 22.0, 23.6, 25.4, 40.0, 44.3, 47.4, 48.1, 67.1, 67.2, 118.5, 120.2 (2C), 121.8, 125.3 (2C), 127.3 (2C), 127.9 (2C), 128.6 (2C), 128.7, 128.9 (2C), 135.7, 141.5 (2C), 144.1 (2C), 151.5, 157.5, 161.6 (2C), 164.4, 169.3, 170.5 ppm. FT-IR (cm^{-1}): ν_{max} 3303, 3194, 3070, 2954, 1702, 1620, 1498, 1449, 1318, 1259, 1076, 739. LC t_R = 2.42 min (conditions B). LC-MS (ESI+): *m/z* (%) = 696.2 (100) $[M+H]^+$, 718.2 (65) $[M+Na]^+$. HRMS (ESI): calcd for $C_{37}H_{37}N_5O_5S_2$: $[M+H]^+$ 696.2314, found 696.2313.

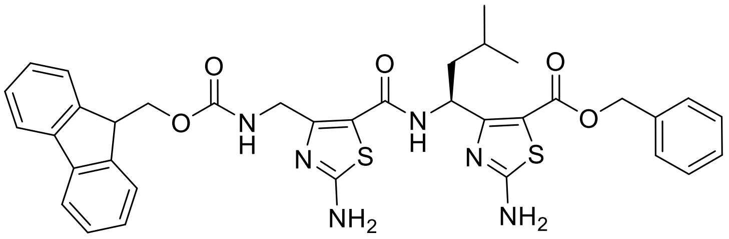

Chemical Formula: $C_{36}H_{36}N_6O_5S_2$
Exact Mass: 696,22
Molecular Weight: 696,84

Benzyl 2-amino 4-(1-{[2-amino-4-({Fmoc-amino}methyl)-1,3-thiazol-5-yl]formamido}-3-methylbutyl)- -1,3-thiazole-5-carboxylate 7b:

White powder, yield 88% (1280 mg). $[\alpha]_D^{20°C}$ = –0.128 (*c* =1.00, CH_3OH). ^{1}H NMR (CD_3OD, 300 MHz) δ = 0.88 (m, 6H), 1.40 – 1.53 (m, 1H), 1.58 – 1.72 (m, 1H), 1.74 – 1.88 (m, 1H), 4.15 (d, *J* = 15.2 Hz, 1H), 4.17 (m, 1H), 4.35 (m, 2H), 4.56 (d, *J* = 15.2 Hz, 1H), 5.24 (s, 2H), 5.80 – 5.96 (m, 1H), 7.20 – 7.40 (m, 9H), 7.56 (m, 2H), 7.74 (d, *J* = 7.4 Hz, 2H), 8.77 (d, *J* = 7.8 Hz, 1H) ppm. ^{13}C NMR (CD_3OD, 75 MHz) δ = 22.0, 23.7, 26.1, 38.9, 44.1, 48.2, 49.3, 67.4, 67.9, 109.9, 118.5, 120.9 (2C), 126.1 (2C), 128.1 (2C), 128.7 (2C), 129.2 (3C), 129.5 (2C), 137.3, 142.5 (2C), 144.9, 145.0, 159.2, 161.5, 163.0, 163.4, 171.0, 173.2, 175.2 ppm. FT-IR (cm^{-1}): ν_{max} 3291, 2958, 1693, 1606, 1490, 1449, 1251, 1131, 1071, 728. LC t_R = 2.13 min (conditions B). LC-MS (ESI+): *m/ z* (%) = 697.2 (55) $[M+H]^+$, 719.2 (15) $[M+Na]^+$. HRMS (ESI): calcd for $C_{36}H_{37}N_6O_5S_2$: $[M+H]^+$ 697.22674, found 697.2277.

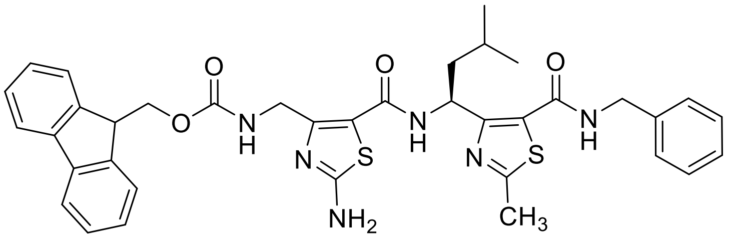

9H-fluoren-9-ylmethyl N-{[2-amino-5-({1-[5-(benzylcarbamoyl)-2-methyl-1,3-thiazol-4-yl]-3-methylbutyl}carbamoyl)-1,3-thiazol-4-yl]methyl}carbamate 7c :
White powder, yield 60% (386 mg). ^{1}H NMR ($CDCl_3$, 300 MHz) δ = 0.83 (d, J = 6.4 Hz, 6H), 1.46 (m, 1H), 1.71 – 1.99 (m, 2H), 2.62 (s, 3H), 4.02 – 4.24 (m, 3H), 4.46 – 4.58 (m, 3H), 4.81 (dd, J = 14.5 – 6.2 Hz, 1H), 5.22 (dd, J = 14.5 – 7.4 Hz, 1H), 6.07 (br., 2H), 6.81 (br., 1H), 7.18 – 7.48 (m, 9H), 7.52 (dd, J = 10.7 – 7.6 Hz, 2H), 7.72 (dd, J = 7.5 – 2.5 Hz, 2H), 8.89 (br., 1H), 10.02 (br., 1H) ppm. ^{13}C NMR ($CDCl_3$, 75 MHz) δ = 19.3, 22.3, 22.4, 24.9, 39.7, 42.7, 43.9, 47.2, 48.3, 66.9, 118.8, 120.1 (2C), 124.9 (2C), 127.1 (2C), 127.2, 127.8 (2C), 128.1 (2C), 128.6 (2C), 131.5, 138.6, 141.3 (2C), 143.7 (2C), 150.0, 153.2, 157.6, 161.7, 162.6, 168.1, 169.4 ppm. FT-IR (cm^{-1}): ν_{max} 3217, 3048, 2956, 1700, 1612, 1556, 1506, 1450, 1327, 1265, 1134, 728. LC t_R = 2.42 min (conditions A). LC-MS (ESI+): m/z (%) = 695.3 (100) $[M+H]^+$, 717.2 (25) $[M+Na]^+$. HRMS (ESI): calcd for $C_{37}H_{39}N_6O_4S_2$: $[M+H]^+$ 695.2474, found 695.2479.

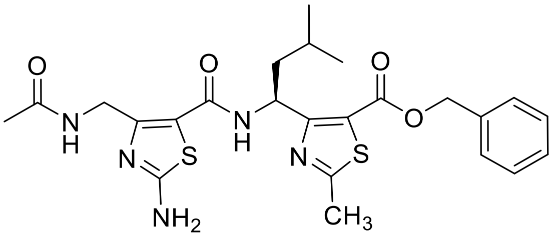

Chemical Formula: $C_{24}H_{29}N_5O_4S_2$
Exact Mass: 515,17
Molecular Weight: 515,65

Benzyl 4-(1-{[2-amino-4-(acetamidomethyl)-1,3-thiazol-5-yl]formamido}-3-methylbutyl)-2-methyl-1,3-thiazole-5-carboxylate 8a:
White powder, yield 65% (100 mg). ^{1}H NMR ($CDCl_3$, 300 MHz) δ = 0.96 (dd, J = 6.3, 3.7 Hz, 6H), 1.55-1.77 (m, 3H), 2.00 (s, 3H), 2.67 (s, 3H), 4.34 (dd, J = 6.2, 14.8 Hz, 1H), 4.58 (dd, J = 6.2, 14.8 Hz, 1H), 5.35 (s, 2H), 5.84 (br., 2H), 6.07 (m, 1H), 6.82 (br., 1H), 7.33-7.48 (m, 5H), 8.12 (br., 1H) ppm. ^{13}C NMR (CD_3OD, 75 MHz) δ = 20.1, 23.0, 23.2, 24.4, 27.2, 40.6, 45.5, 49.9, 69.0, 119.4, 123.9, 130.3, 130.3 (2C), 130.5 (2C), 138.0, 152.2, 163.4, 164.5, 165.8, 172.5, 173.0, 174.6 ppm. FT-IR (cm^{-1}): ν_{max} 3016, 2970, 1738, 1513, 1435, 1366, 1229, 1217, 1092, 901. LC t_R = 2.79 min (conditions B). LC-MS (ESI+): m/z (%) = 516.2 (100) [M+H]$^+$. HRMS (ESI): calcd for $C_{24}H_{30}N_5O_4S_2$: [M+H]$^+$ 516.1739, found 516.1738.

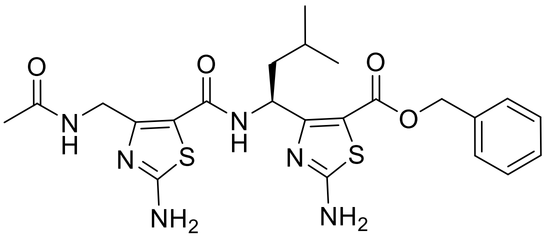

Chemical Formula: $C_{23}H_{28}N_6O_4S_2$
Exact Mass: 516,16
Molecular Weight: 516,64

benzyl 2-amino-4-(1-{[2-amino-4-(acetamidomethyl)-1,3-thiazol-5-yl]formamido}-3-methylbutyl)-1,3-thiazole-5-carboxylate 8b:
White powder, yield 18% (34 mg). ^{1}H NMR (CD_3OD, 300 MHz) δ = 0.95 (dd, J = 6.4, 3.9 Hz, 6H), 1.52 – 1.90 (3m, 3H), 2.0 (s, 3H), 4.29 (d, J = 14.9 Hz, 1H), 4.56 (d, J = 14.9 Hz, 1H), 5.29 (d, J = 1.6 Hz, 2H), 5.92 (dd, J = 9.7, 5.1 Hz, 1H), 7.33 – 7.47 (m, 5H) ppm. ^{13}C NMR (CD_3OD, 75 MHz) δ = 23.0, 23.2, 24.5, 27.2, 40.7, 45.5, 49.9, 68.2, 110.7, 119.3, 130.0, 130.1 (2C), 130.4 (2C), 138.5, 152.3, 164.1, 164.3, 165.6, 172.4, 174.2, 174.5 ppm. FT-IR (cm^{-1}): ν_{max} 3019, 2971, 2944, 1739, 1435, 1366, 1229, 1217, 1208. LC t_R = 2.79 min (conditions B). LC-MS (ESI+): m/z (%) = 517.2 (100) [M+H]$^+$. HRMS (ESI): calcd for $C_{23}H_{28}N_6O_4S_2$: [M+H]$^+$ 517.1692, found 517.1689.

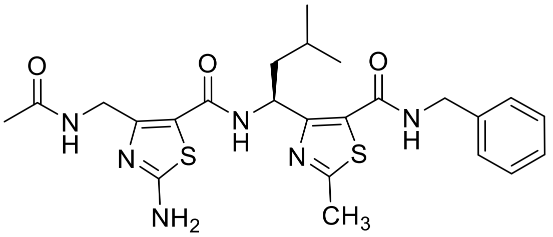

Chemical Formula: $C_{24}H_{30}N_6O_3S_2$
Exact Mass: 514,18
Molecular Weight: 514,66

2-amino-N-{1-[5-(benzylcarbamoyl)-2-methyl-1,3-thiazol-4-yl]-3-methylbutyl}-4-(acetamidomethyl)-1,3-thiazole-5-carboxamide 8c:

White powder, yield 61% (110 mg). ^{1}H NMR (CD_3OD, 300 MHz) δ = 0.77 (d, 6.6 Hz, 3H), 0.80 (d, 6.6 Hz, 3H), 1.32 (hept, J = 6.6 Hz, 1H), 1.84 (m, 2H), 1.97 (s, 3H), 2.64 (s, 3H), 4.19 (d, J = 14.9, 1H), 4.44 (d, J = 14.9 Hz, 1H), 4.46 (d, J = 14.7 Hz, 1H), 4.69 (d, J = 14.7 Hz, 1H), 5.26 (t, J = 7.7 Hz, 1H), 7.20 – 7.34 (m, 3H), 7.39 (d, J = 6.8 Hz, 2H) ppm. ^{13}C NMR (CD_3OD, 75 MHz) δ = 19.9, 23.2, 23.3, 23.8, 26.9, 40.7, 44.5, 45.6, 50.3, 118.2, 129.2, 129.9 (2C), 130.5 (2C), 131.9, 140.5, 153.3, 156.8, 164.2, 165.4, 170.8, 172.6, 174.6 ppm. FT-IR (cm^{-1}): ν_{max} 3050, 2971, 1739, 1622, 1509, 1435, 1366, 1229, 1217, 1208. LC t_R = 2.39 min (conditions B). LC-MS (ESI+): m/z (%) = 515.1 (100) $[M+H]^+$, 537.1 $[M+Na]^+$. HRMS (ESI): calcd for $C_{24}H_{31}N_6O_3S_2$: $[M+H]^+$ 515.1899, found 515.1899.

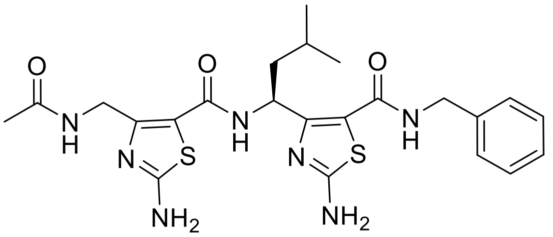

Chemical Formula: $C_{23}H_{29}N_7O_3S_2$
Exact Mass: 515,18
Molecular Weight: 515,65

2-amino-N-{1-[2-amino-5-(benzylcarbamoyl)-1,3-thiazol-4-yl]-3-methylbutyl}-4-(acetamidomethyl)-1,3-thiazole-5-carboxamide 8d:

White powder, yield 24% (21 mg). ^{1}H NMR (CD_3OD, 300 MHz) δ = 0.84 (d, 6.6 Hz, 6H), 0.89 (d, 6.6 Hz, 6H), 1.48 (m, 1H), 1.85 (t, J = 7.4 Hz, 2H), 2.03 (s, 3H), 4.29 (d, J = 15.4, 1H), 4.48 (d, J = 14.8 Hz, 1H), 4.55 (d, J = 15.4 Hz, 1H), 4.67 (d, J = 14.8 Hz, 1H), 5.28 (t, J = 7.4 Hz, 1H), 7.23 – 7.45 (m, 5H) ppm. ^{13}C NMR (CD_3OD, 75 MHz) δ = 22.2, 22.4, 22.8, 26.1, 38.3, 42.8, 44.7, 48.8, 117.2, 118.5, 128.4, 129.0 (2C), 129.6 (2C), 139.6, 146.1, 148.5, 162.8, 163.2, 171.6, 171.7, 174.4 ppm. FT-IR (cm^{-1}): ν_{max} 3229, 3070, 1639, 1569, 1441, 1307, 1202, 1141. LC t_R = 2.16 min (conditions B). LC-MS (ESI+): m/z (%) = 516.1 (75) $[M+H]^+$, 538.1 (100) $[M+Na]^+$. HRMS (ESI): calcd for $C_{23}H_{30}N_7O_3S_2$: $[M+H]^+$ 516.1852, found 516.1849.

General procedure F : Benzoylation of dimers and Synthesis of 9a-c and 10a-c

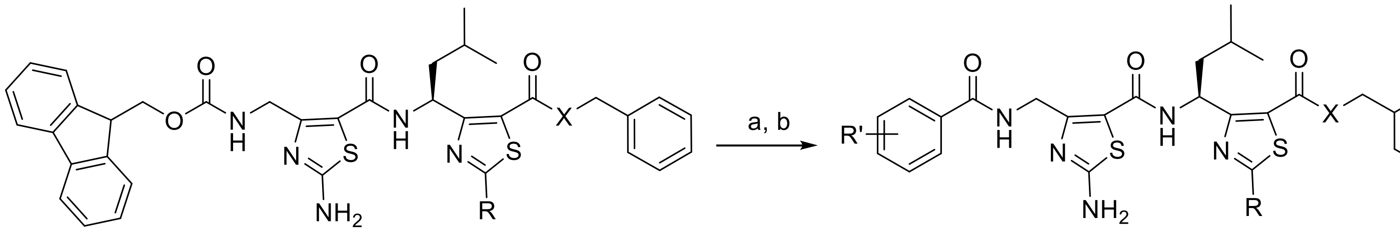

7a-c

9a-c: R' = 4-hydroxy-2-methyl

10a-c: R' = 2,4,6-trihydroxy

7a-c ; 9a-c; 10a-c:

a : R^1 = Fmoc ; X = O ; R = CH_3

b : R^1 = Fmoc ; X = O ; R = NH_2

c : R^1 = Fmoc ; X = NH ; R = CH_3

Scheme S9 : Synthesis of dimers **9a-c** and **10a-c** . reagents and conditions: a) diethylamine / DMF (1:9 vv), RT, 30 min; b) EDC.HCl, NMM, HOBt, 2,4,6-trihydroxybenzoic acid or 2-methyl-4-hydroxybenzoic acid, DMF, 0°C to RT, 3h.

Compounds	X	R	R'	Yields
**9a**	-O-	CH_3	2- CH_3-4-OH	70%
**9b**	-O-	NH_2	2- CH_3-4-OH	64%
**9c**	-NH-	CH_3	2,4,6-triOH	12%
**10a**	-O-	CH_3	2,4,6-triOH	34%
**10b**	-O-	NH_2	2,4,6-triOH	36%
**10c**	-NH-	CH_3	2- CH_3-4-OH	33%

The dimers **7a-c** were deprotected following the general procedure C2. The corresponding amino derivatives were engaged in the next step without further purification. to afford the free amines NH_2-{H, NH_2}-ATC-NH-{iBu, CH_3}-ATC-OBn, NH_2-{H, NH_2}-ATC-NH-{iBu, NH_2}-ATC-OBn, NH_2-{H, NH_2}-ATC-NH-{iBu, CH_3}-ATC-NHBn and NH_2-{H, NH_2}-ATC-NH-{iBu, NH_2}-ATC-NHBn.

To a solution of 2,4,6-trihydroxybenzoic acid or 2-methyl-4-hydroxybenzoic acid (0.963 mmol, 1.0 equiv.) in 20 ml anhydrous DMF at 0°C were added EDC.HCl (1.15 mmol, 1.2 equiv.), NMM (1.15 mmol, 1.2 equiv.) and HOBt (1.15 mmol, 1.2 equiv.). After 1 h stirring at RT, the amino-dipeptide was added. The mixture was stirred 4 h at RT. The reaction was monitored by HPLC until completion. Activated acid can be added if needed. Solvent was removed under *vacuum*. The crude was purified by reverse phase chromatography on a C18 column with a gradient of H_2O / Acetonitrile containing 0.1% TFA

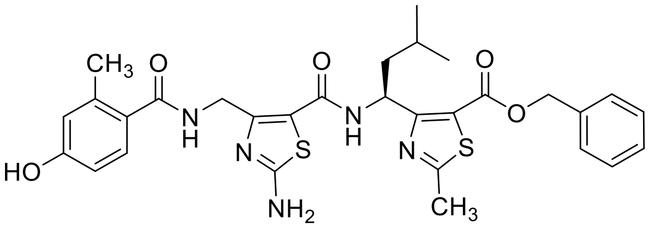

Chemical Formula: $C_{30}H_{33}N_5O_5S_2$
Exact Mass: 607,19
Molecular Weight: 607,74

Benzyl 4-{1-[(2-amino-4-{[(4-hydroxy-2-methylphenyl)formamido]methyl}-1,3-thiazol-5-yl)formamido]-3-methylbutyl}-2-methyl-1,3-thiazole-5-carboxylate (9a)

White powder, yield 70% % (110 mg). ^{1}H NMR (CD_3OH, 400 MHz) δ = 0.94 (d, J = 6.6 Hz, 3H), 0.95 (d, J = 6.7 Hz, 3H), 1.57 – 1.65 (m, 1H), 1.72 – 1.83 (m, 1H), 1.92 – 2.01 (m, 1H), 2.38 (s, 3H), 2.66 (s, 3H), 4.31 (dd, J = 14.8 – 5.6 Hz, 1H), 4.79 (dd, J = 14.8 – 5.6 Hz, 1H), 5.35 (s, 2H), 6.00 – 6.07 (m, 1H), 6.62 (dd, J = 8.3 – 2.4 Hz, 1H), 6,65 (d, J = 2.4 Hz, 1H), 7.07 (br., 2H), 7.32 – 7.41 (m, 4H), 7,46 (d, J = 8.3 Hz, 2H), 8.45 (br, 1H), 9.53 (br., 1H) ppm. ^{13}C NMR (CD_3OH, 100 MHz) δ = 19.2, 20.4, 21.9, 23.5, 26.2, 40.1, 44.6, 68.0, 113.3, 118.6 (2C), 123.0, 127.6, 129.3, 129.4, 129.5 (2C), 130.2 (2C), 137.0, 140.0, 151.4, 160.4, 162.4, 163.8, 165.0, 171.7, 172.0, 173.1, 173.1. LC t_R = 1.97 min (conditions B). LC-MS (ESI+): *m/z* (%) = 608.3 (100) $[M+H]^+$, 630.3 (21) $[M+Na]^+$. HRMS (ESI): calcd for $C_{30}H_{34}N_5O_5S_2$: $[M+H]^+$ 608.2001, found 608.2004.

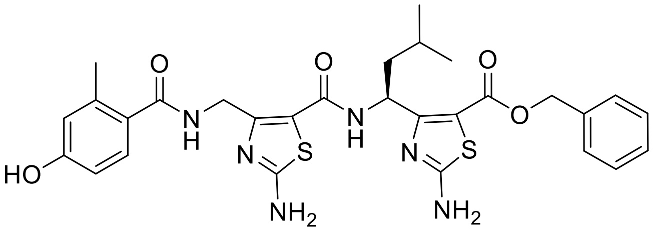

Chemical Formula: $C_{29}H_{32}N_6O_5S_2$
Exact Mass: 608,19
Molecular Weight: 608,73

benzyl 2-amino-4-{1-[(2-amino-4-{[(4-hydroxy-2-methylphenyl)formamido]methyl}-1,3-thiazol-5-yl)formamido]-3-methylbutyl}-1,3-thiazole-5-carboxylate (9b)

White powder, yield 54% (284 mg). ^{1}H NMR (CD_3OH, 600 MHz) δ = 0.91 (d, J = 6.6 Hz, 3H), 0.92 (d, J = 6.6 Hz, 3H), 1.55 – 1.60 (m, 1H), 1.69 – 1.76 (m, 1H), 1.84 – 1.90 (m, 1H), 2.36 (s, 3H), 4.42 (dd, J = 15.2 – 5.3 Hz, 1H), 4.83 (d, J = 15.4 Hz, 1H) (signal hidden by H_2O, visible on spectra in CD_3OD), 5.27 (dd, J = 15.2 – 12.4 Hz, 2H), 5.87 – 5.92 (m, 1H), 6.60 (dd, 8.4 – 2.3 Hz, 1H), 6.64 (d, J = 2.3 Hz, 1H), 7.28 – 7.42 (m, 6H), 8.46 (br., 1H), 9.28 (br., 1H) ppm. ^{13}C NMR (CD_3OH, 150 MHz) δ = 20.4, 21.9, 23.5, 26.2, 39.0, 44.4, 49.9, 67.4, 110.0, 113.4, 118.4, 118.7, 127.1, 127.2, 129.2 (3C), 129.5 (2C), 130.3, 137.5, 140.2, 160.6, 162.4, 163.0, 163.4, 171.4, 173.3, 173.4 ppm. FT-IR (cm^{-1}): ν_{max} 3184, 2960, 1633, 1576, 1496, 1436, 1379, 1298, 1256, 1188, 1135, 1093, 721. LC t_R = 1.86 min (conditions A). LC-MS (ESI+): *m/z* (%) = 609,1 (100) $[M+H]^+$. HRMS (ESI): calcd for $C_{29}H_{32}N_6O_5S_2$: $[M+H]^+$ 609.1954, found 609.1960.

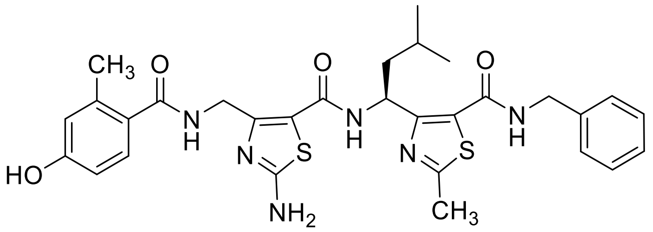

Chemical Formula: $C_{30}H_{34}N_6O_4S_2$
Exact Mass: 606,21
Molecular Weight: 606,76

4-{1-[(2-amino-4-{[(4-hydroxy-2-methylphenyl)formamido]methyl}-1,3-thiazol-5-yl)formamido]-3-methylbutyl}-N-benzyl-2-methyl-1,3-thiazole-5-carboxamide (9c)

White powder, yield 33% (72 mg). ^{1}H NMR (CD_3OD, 300 MHz) δ = 0.81 (d, J = 6.4 Hz, 3H), 0.83 (d, J = 6.4 Hz, 3H), 1.43 (sept, J = 6.7 Hz, 1H), 1.79 – 1.97 (m, 2H), 2.39 (s, 3H), 2.66 (s, 3H), 4.36 (d, J = 15.3 Hz, 1H), 4.47 (d, J = 14.7 Hz, 1H), 4.67 (d, J = 14.7 Hz, 1H), 4.69 (d, J = 15.3 Hz, 1H), 5.36 (t, J = 7.7 Hz, 1H), 6.64 (td, 8.3 – 2.3 Hz, 1H), 6.66 (s, 1H), 7.22 – 7.42 (m, 6H) ppm. ^{13}C NMR (CD_3OD, 75 MHz) δ = 19.1, 20.6, 22.6, 22.7, 26.1, 38.4, 43.8, 44.7, 49.0, 113.5, 117.8, 118.8, 126.7, 128.4, 129.0 (2C), 129.6 (2C), 130.6, 139.6, 140.6, 145.2, 156.2 (2C), 160.8, 162.8, 163.3, 170.0, 171.5, 173.4 ppm. FT-IR (cm^{-1}): ν_{max} 3217, 3021, 2971, 1739, 1639, 1576, 1441, 1203, 1141. LC t_R = 1.86 min (conditions A). LC-MS (ESI+): m/z (%) = 607,3 (100) $[M+H]^+$. HRMS (ESI): calcd for $C_{30}H_{35}N_6O_4S_2$: $[M+H]^+$ 607.2161, found 607.2160.

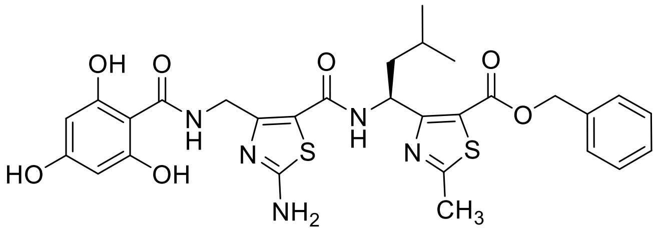

Chemical Formula: $C_{29}H_{31}N_5O_7S_2$
Exact Mass: 625,17
Molecular Weight: 625,72

Benzyl 4-{1-[(2-amino-4-{[(2,4,6-trihydroxyphenyl)formamido]methyl}-1,3-thiazol-5-yl)formamido]-3-methylbutyl}-2-methyl-1,3-thiazole-5-carboxylate (10a)

White powder, yield 34% (43 mg). ^{1}H NMR (CD_3OH, 600 MHz) δ = 0.93 (d, J = 6.7 Hz, 3H), 0.94 (d, J = 6.7 Hz, 3H), 1.59 – 1.64 (m, 1H), 1.70 – 1.77 (m, 1H), 1.91 – 1.96 (m, 1H), 2.65 (s, 3H), 4.44 (dd, J = 15.0 – 3.8 Hz, 1H), 4.74 (dd, J = 14.9 – 5.6 Hz, 1H), 5.31 (s, 2H), 5.85 (s, 2H), 6.03 – 6.07 (m, 1H), 7.31 (d, J = 7.2 Hz, 1H), 7.35 (t, J = 7.0 Hz, 2H), 7.42 (d, J = 7.2 Hz, 2H), 7.76 (br., 2H), 9.37 (br., 1H), 9.77 (br., 1H) ppm. ^{13}C NMR (CD_3OD, 75 MHz) δ = 20.2, 22.8, 24.5, 27.3, 37.9, 45.6, 69.1, 97.0, 97.0, 97.5, 116.5, 120.5, 124.1, 130.4 (3C), 130.5 (2C), 137.9, 141.6, 162.1, 163.3, 164.4, 165.1, 165.3, 165.3, 172.3, 173.4, 173.9 ppm. FT-IR (cm^{-1}): ν_{max} 3337, 3207, 2959, 1613, 1585, 1539, 1513, 1455, 1386, 1254, 1188, 1135, 1078, 827, 799, 722, 696. LC t_R = 2.97 min (conditions B). LC-MS (ESI+): m/z (%) = 626.1 (100) $[M+H]^+$. HRMS (ESI): calcd for $C_{29}H_{32}N_5O_7S_2$: $[M+H]^+$ 626.1743, found 626.1744.

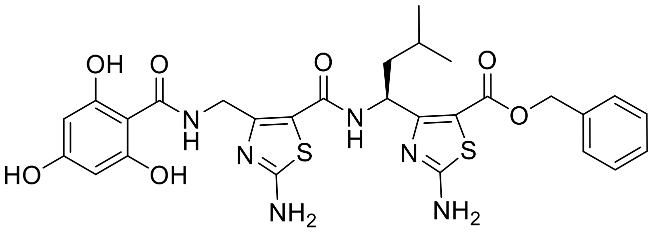

Chemical Formula: $C_{28}H_{30}N_6O_7S_2$
Exact Mass: 626,16
Molecular Weight: 626,70

Benzyl 2-amino-4-{1-[(2-amino-4-{[(2,4,6-trihydroxyphenyl)formamido]methyl}-1,3-thiazol-5-yl)formamido]-3-methylbutyl}-1,3-thiazole-5-carboxylate (10b)

White powder, yield 36% (22 mg). ^{1}H NMR (CD_3OH, 600 MHz) δ = 0.92 (d, J = 6.6 Hz, 3H), 0.93 (d, J = 6.6 Hz, 3H), 1.55 – 1.62 (m, 1H), 1.68 – 1.76 (m, 1H), 1.85 – 1.91 (m, 1H), 4.49 (dd, J = 15.4 – 5.3 Hz, Hz, 1H), 4.82 (d, J = 15.4 Hz, 1H) (signal hidden by H_2O, visible on spectra in CD_3OD), 5.26 (dd, J = 15.4 – 12.4 Hz, 2H), 5.87 (s, 2H), 5.87 – 5.98 (m, 1H), 7.30 (t, J = 7.1 Hz, 1H), 7.35 (t, J = 7.5 Hz, 2H), 7.41 (d, J = 7.8 Hz, 2H), 9.37 (br., 1H), 9.72 (br., 1H) ppm. ^{13}C NMR (CD_3OH, 75 MHz) δ = 21.9, 23.6, 26.3, 37.5, 44.5, 49.8, 67.4, 96.1, 96.2, 96.3, 110.1, 119.5, 119.5, 129.1, 129.2 (2C), 129.5 (2C), 137.5, 142.6, 161.6, 163.0, 163.3, 163.5, 164.3, 171.5, 172.9, 173.3 ppm. FT-IR (cm^{-1}): ν_{max} 3324, 3197, 2958, 1601, 1584, 1538, 1495, 1455, 1385, 1263, 1165, 1075, 826, 722, 696. LC t_R = 1.84 min (conditions A). LC-MS (ESI+): m/z (%) = 627.1 (70) $[M+H]^+$, 649.0 (50) $[M+Na]^+$. HRMS (ESI): calcd for $C_{28}H_{31}N_6O_7S_2$: $[M+H]^+$ 627.1696, found 627.1700.

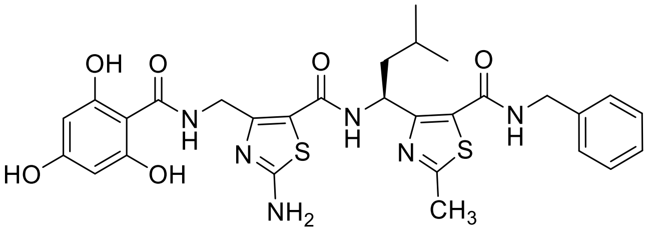

Chemical Formula: $C_{29}H_{32}N_6O_6S_2$
Exact Mass: 624,18
Molecular Weight: 624,73

4-{1-[(2-amino-4-{[(2,4,6-trihydroxyphenyl)formamido]methyl}-1,3-thiazol-5-yl)formamido]-3-methylbutyl}-N-benzyl-2-methyl-1,3-thiazole-5-carboxamide (10c)

White powder, yield 12% (28 mg). ^{1}H NMR (CD_3OH, 600 MHz) δ = 0.82 (d, J = 6.6 Hz, 3H), 0.84 (d, J = 6.6 Hz, 3H), 1.43 (sept, J = 6.6 Hz, 1H), 1.83 – 1.88 (m, 1H), 1.89 – 1.95 (m, 1H), 2.67 (s, 3H), 4.46 – 4.59 (m, 3H), 4.68 (d, J = 14.7 – 4.9 Hz, 1H), 5.36 – 5.42 (m, 1H), 5.86 (s, 2H), 7.24 (t, J = 7.4 Hz, 1H), 7.31 (t, J = 7.4 Hz, 2H), 7.40 (d, J = 7.4 Hz, 2H), 9.36 (br., 1H), 9.86 (br., 1H), 9.95 (br., 1H) ppm. ^{13}C NMR (CD_3OH, 75 MHz) δ = 19.0, 22.6 (2C), 26.1, 38.0, 43.9, 44.7, 49.8, 96.1 (2C), 96.3, 118.3, 128.3, 128.9 (2C), 129.5 (2C), 130.5, 139.6, 146.1, 156.0 (2C), 162.1, 163.3, 163.5, 164.2, 170.0, 171.7, 172.8 ppm. FT-IR (cm^{-1}): ν_{max} 2955, 1611, 1584, 1542, 1508, 1462, 1305, 1268, 1232, 1184, 1135, 1074, 830, 779, 668. LC t_R = 2.67 min (conditions B). LC-MS (ESI+): m/z (%) = 625,2 (100) $[M+H]^+$, 647.0 (23) $[M+Na]^+$. HRMS (ESI): calcd for $C_{29}H_{33}N_6O_6S_2$: $[M+H]^+$ 625.1903, found 625.1909.

Solid phase synthesis of 10d

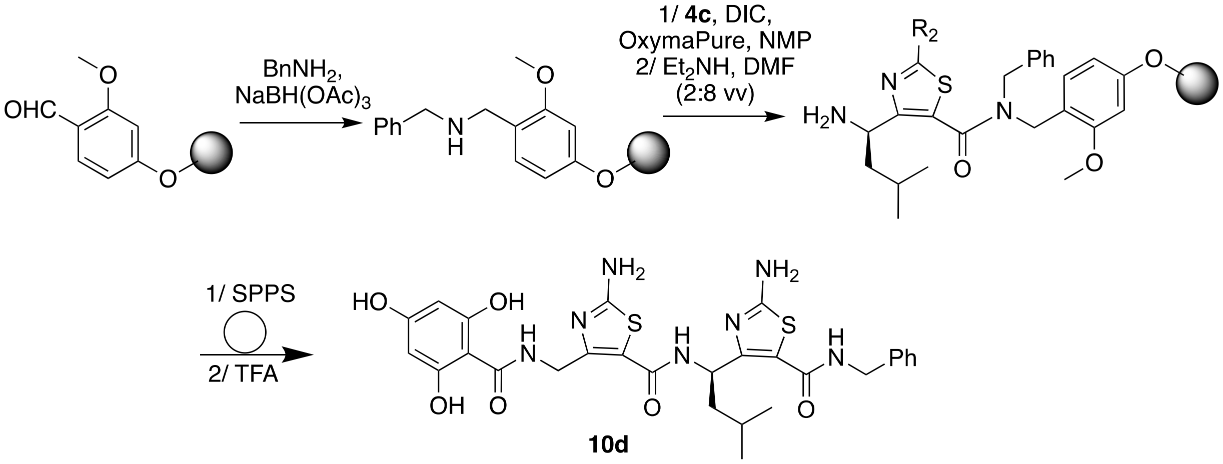

Scheme S10 : Synthesis of dimer **10d** .

The acid sensitive methoxybenzaldehyde (AMEBA) polystyrene resin (0.86 mmol.g^{-1}) was used as solid support [24]. To 0.5 g of resin in 10 ml of DMF with AcOH 5% vv, benzylamine (470 µl, 461 mg, 4.3 mmol, 10 equiv.) and sodium borohydride (272 mg, 4.3 mmol, 10 equiv.) were added. After 15 h stirring at RT, the amine resin was washed by DMF, DCM and MeOH.

General procedure for peptide solid-phase synthesis

Resin was soaked in *N*-methylpyrrolidone (NMP) for 5-10 minutes and filtered. Fmoc-ATC-OH (2.0 equiv.), N,N′-diisopropylcarbodiimide (DIC, 2.0 equiv.), ethyl (hydroxyimino) cyanoacetate (oxyma Pure®, 2.0 equiv.), and NMP were added in this order for each peptide coupling (overnight at RT). Resin was washed using the following procedure: 3×DMF, 3×DCM, 3×DMF, 3×DCM. Deprotection at the *N*-terminus was performed using a 20 % piperidine/DMF solution (3×10 min at r.t.) and the resin was then washed before the next coupling. Deprotection and coupling steps were monitored by Kaiser test.

Synthesis of dimer **10d**

After deprotection at the *N*-terminus with 20 % piperidine/DMF (3×10 min at r.t.), *N*-terminus coupling was carried out by using 2,4,6-trihydroxybenzoic acid (3 equiv.), EDC.HCl (3 equiv.), NMM (3 equiv.), HOBt (3 equiv.) at RT in NMP (10 ml). The γ-peptide was then cleaved from the resin with 10 ml of TFA (1h at RT). The resin was washed (1×MeOH) and filtered. The filtrate was evaporated under reduced pressure. Dimer **10d** was lyophilized then purified by preparative RP-HPLC on a Waters system controller equipped with a C_{18} Waters Delta-Pack column (100×40 mm, 100 Å) flow: 50 ml min^{-1}; UV detection at 214 nm using a Waters 486 Tunable Absorbance Detector and a linear gradient of A = H_2O (0.1 % TFA) and B = ACN (0.1 % TFA).

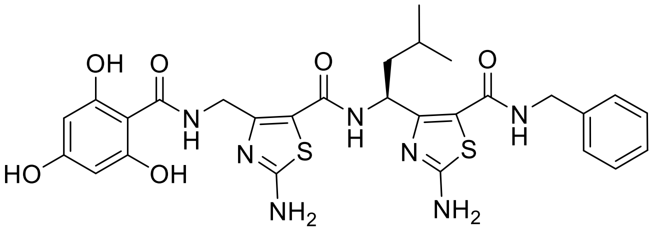

Chemical Formula: $C_{28}H_{31}N_7O_6S_2$
Exact Mass: 625,18
Molecular Weight: 625,72

2-amino-N-{1-[2-amino-5-(benzylcarbamoyl)-1,3-thiazol-4-yl]-3-methylbutyl}-4-{[(2,4,6-trihydroxyphenyl)formamido]methyl}-1,3-thiazole-5-carboxamide 10d:

White powder, yield 10% (8.7 mg). ^{1}H NMR (CD_3OD, 300 MHz) δ = 0.84 (d, J = 6.4 Hz, 3H), 0.86 (d, J = 6.4 Hz, 3H), 1.50 (sept, J = 6.7 Hz, 1H), 1.74 – 1.92 (m, 2H), 4. 44 (d, J = 14.7 Hz, 1H), 4. 46 (d, J = 15.2 Hz, 1H), 4. 64 (d, J = 15.0 Hz, 2H), 5.31 (t, 1H), 5.88 (s, 2H), 7.21 – 7.40 (m, 5H) ppm.^{13}C NMR (CD_3OD, 75 MHz) δ = 22.7 (2C), 26.2, 37.9, 43.5, 44.6, 71.3, 96.1 (2C), 96.3, 118.3, 119.7, 128.3, 128.9 (2C), 129.6 (2C), 139.9, 145.8, 152.3, 162.3, 162.8, 163.0, 163.4, 163.7, 164.2, 171.7, 172.7 ppm. LC t_R = 1.55 min (conditions A). LC-MS (ESI+): *m/ z* (%) = 626.2 (75) $[M+H]^+$, 648.1 (5) $[M+Na]^+$. HRMS (ESI): calcd for $C_{28}H_{32}N_7O_6S_2$: $[M+H]^+$ 626.1855, found 626.1856.

III. NMR characterization of dimers folding

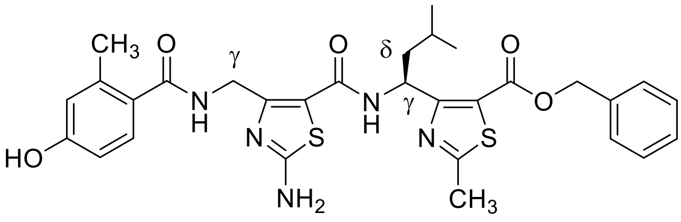

ATC 1 ATC 2

Table S1: ^{1}H NMR chemical shifts for **9a** in CD_3OH at 293 K

ATC number	HN	$^{\gamma}$CH	$^{\delta}$CH	other
ATC 1	8.44	4.31 and 4.79	-	
ATC 2	9.52	6.03	1.61 and 1.95	CH_3: 2.66

Table S2: Coupling Constants $^3J(NH, ^{\gamma}CH)$ (in Hz) for **9a** . Values were measured in CD_3OH at 293K.

ATC number	
ATC 1	5.7
ATC 2	7.9

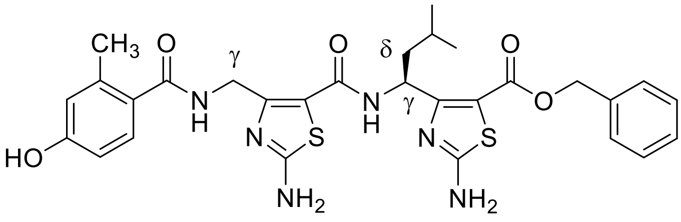

ATC 1 ATC 2

Table S3: ^{1}H NMR chemical shifts for **9b** in CD_3OH at 293 K

ATC number	HN	$^{\gamma}$CH	$^{\delta}$CH	other
ATC 1	8.46	4.42 and 4.83	-	
ATC 2	9.28	5.90	1.57 and 1.86	

Table S4: Coupling Constants $^3J(NH, ^{\gamma}CH)$ (in Hz) for **9b**. Values were measured in CD_3OH at 293K.

ATC number	
ATC 1	nd
ATC 2	7.6

nd: values could not be determined.

Table S5: Inter-residue NOE correlations in **9b** observed in the ROESY spectrum CD_3OH at 293K. Strong < 2.7 Å. 2.7 Å < Medium < 3.3 Å. 3.3 Å < Weak.

**NOE correlations**	**Intensity**
1.NH-2.NH	nd
1.Hγ-2.NH	m
1.Hγ-2. Hδ	nd

nd: not detected

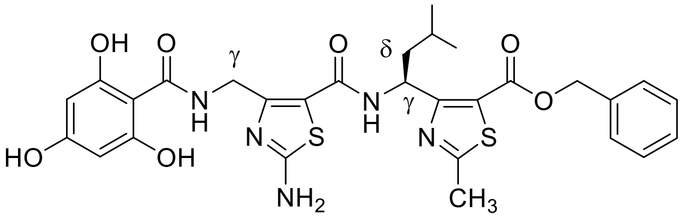

ATC 1 ATC 2

Table S6: ^1H NMR chemical shifts for **10a** in CD_3OH at 293 K

ATC number	HN	$^{\gamma}CH$	$^{\delta}CH$	other
ATC 1	9.37	4.44 and 4.74	-	
ATC 2	9.77	6.05	1.62 and 1.93	CH_3: 2.65

Table S7: Coupling Constants $^3J(NH, ^{\gamma}CH)$ (in Hz) for **10a**. Values were measured in CD_3OH at 293K.

ATC number	
ATC 1	5.6
ATC 2	8.0

Table S8: Inter-residue NOE correlations in **10a** observed in the ROESY spectrum CD_3OH at 293K. Strong < 2.7 Å. 2.7 Å < Medium < 3.3 Å. 3.3 Å < Weak.

**NOE correlations**	**Intensity**
1.NH-2.NH	nd
1.Hγ-2.NH	m
1.Hγ-2. Hδ	w
1.Hγ-2. $^{\tau 1}CH_3$	w

nd: not detected

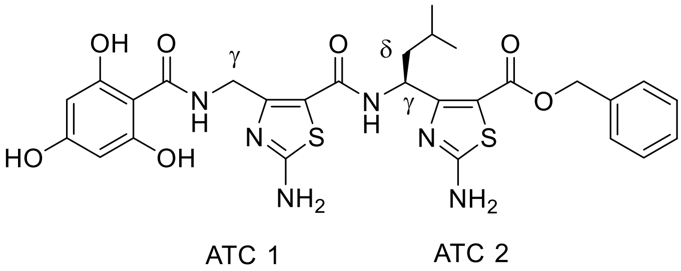

Table S9: ^{1}H NMR chemical shifts for **10b** in CD_3OH at 293 K

ATC number	HN	$^{\gamma}$CH	$^{\delta}$CH	other
ATC 1	9.37	4.49 and 4.82	-	
ATC 2	9.72	5.92	1.58 and 1.88	

Table S10: Coupling Constants $^3J(NH, {}^{\gamma}CH)$ (in Hz) for **10b**. Values were measured in CD_3OH at 293K.

ATC number	
ATC 1	nd
ATC 2	nd

nd: values could not be determined.

Table S11: Inter-residue NOE correlations in **10b** observed in the ROESY spectrum CD_3OH at 293K. Strong < 2.7 Å. 2.7 Å < Medium < 3.3 Å. 3.3 Å < Weak.

**NOE correlations**	**Intensity**
1.NH-2.NH	nd
1.Hγ-2.NH	m
1.Hγ-2. Hδ	nd

nd: not detected

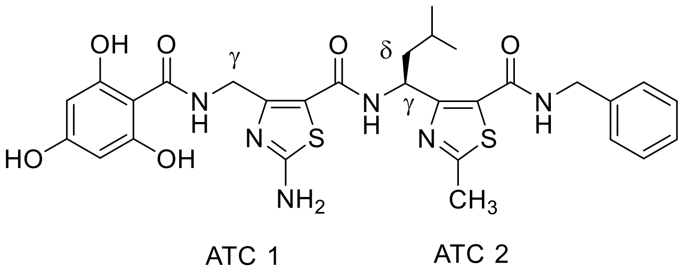

Table S12: ^{1}H NMR chemical shifts for **10c** in CD_3OH at 293 K

ATC number	HN	$^{\gamma}$CH	$^{\delta}$CH	other
ATC 1	9.36	4.51	-	
ATC 2	9.95	5.39	1.85 and 1.92	CH_3: 2.67
NH-CH_2-Ph	9.86			CH_2: 4.68 and 4.49

Table S13: Coupling Constants $^3J(NH, {}^{\gamma}CH)$ (in Hz) for **10c**. Values were measured in CD_3OH at 293K.

ATC number	
ATC 1	5.6
ATC 2	7.2
NH-CH_2-Ph	5.22

nd: not detected

Table S14: Inter-residue NOE correlations in **10c** observed in the ROESY spectrum CD_3OH at 293K. Strong < 2.7 Å. 2.7 Å < Medium < 3.3 Å. 3.3 Å < Weak.

**NOE correlations**	**Intensity**
1.NH-2.NH	nd
1.Hγ-2.NH	m
1.Hγ-2. Hδ	w
1.Hγ-2. $^{\tau1}CH_3$	w
2.NH-NHBn	nd
2.Hγ-NHBn	s

nd: not detected

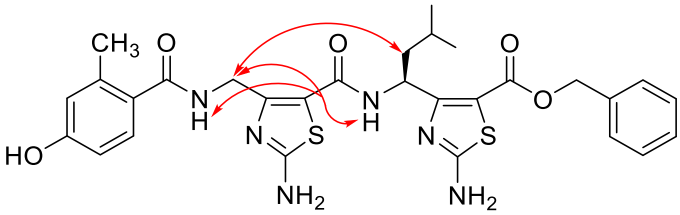

Figure S1: ROESY spectra of **9b** in CD3OH at 293K:

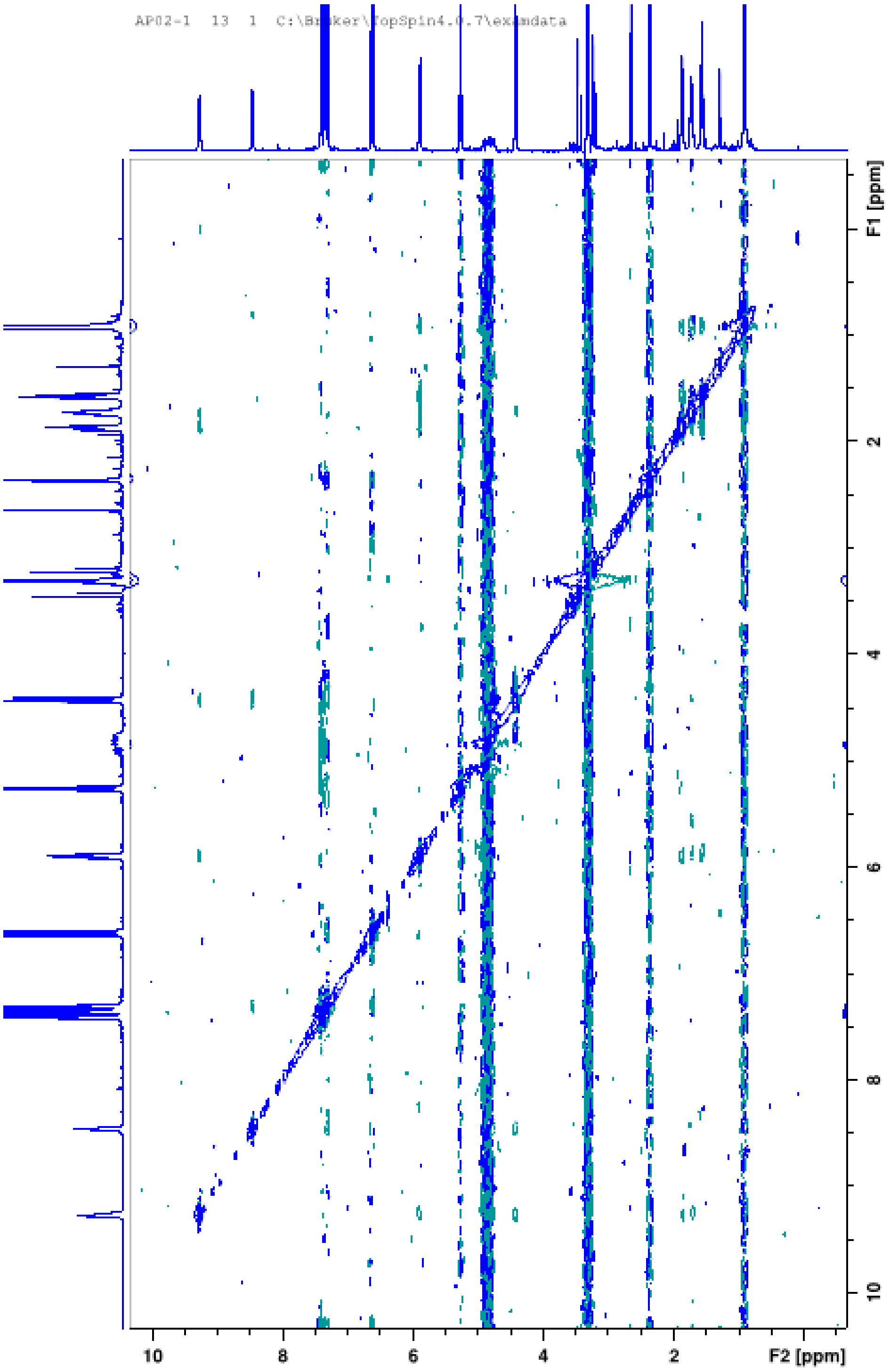

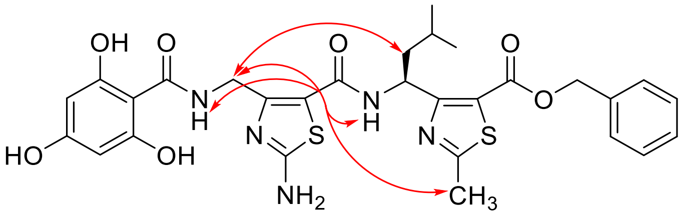

Figure S2: ROESY spectra of **10a** in CD_3OH at 293K:

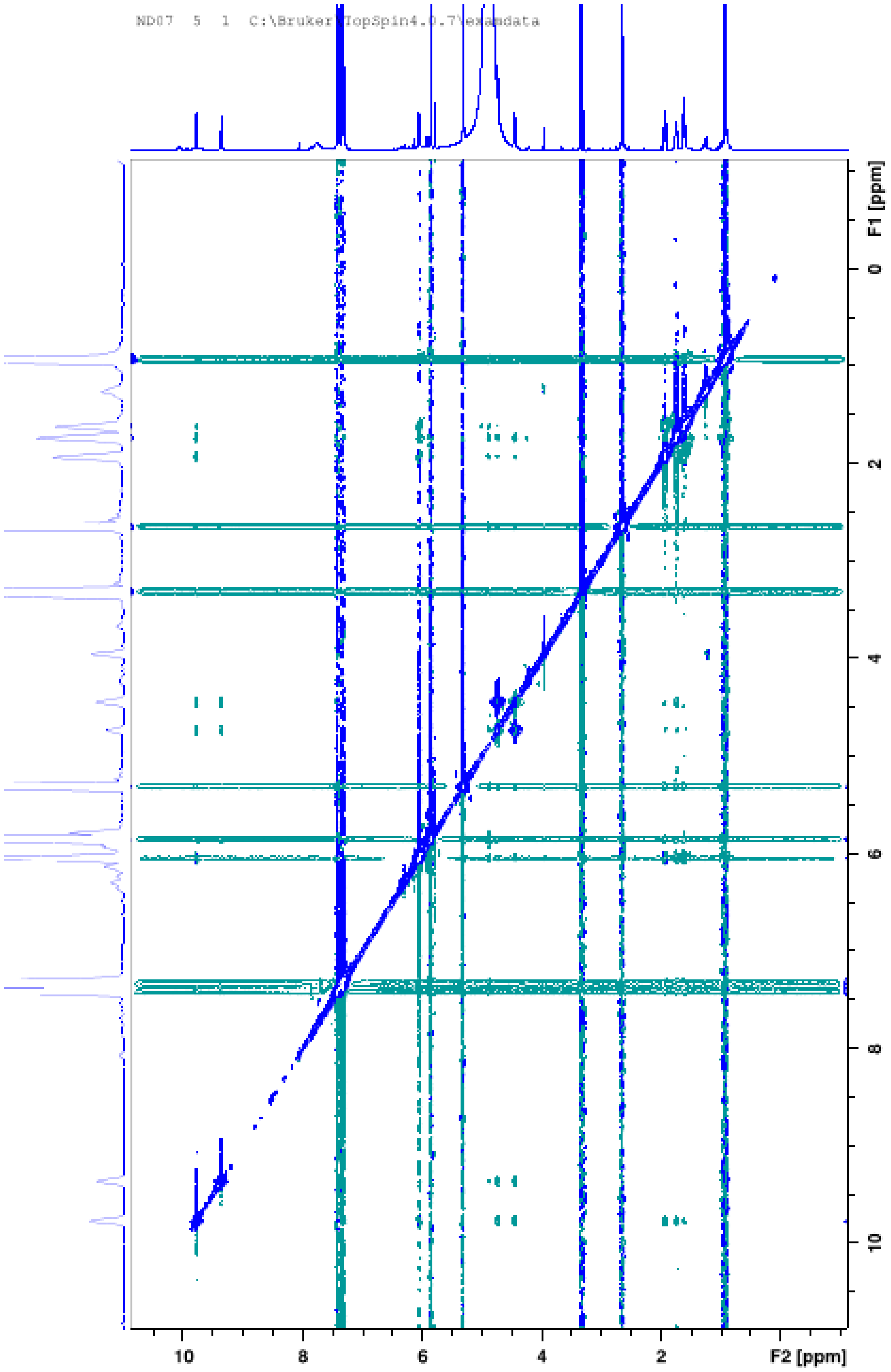

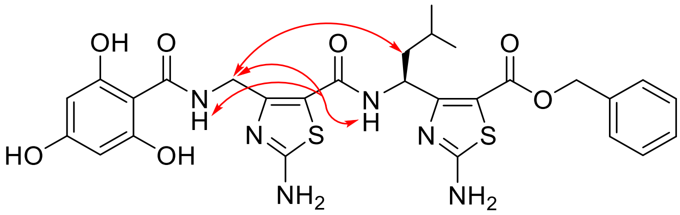

Figure S3: ROESY spectra of **10b** in CD3OH at 293K:

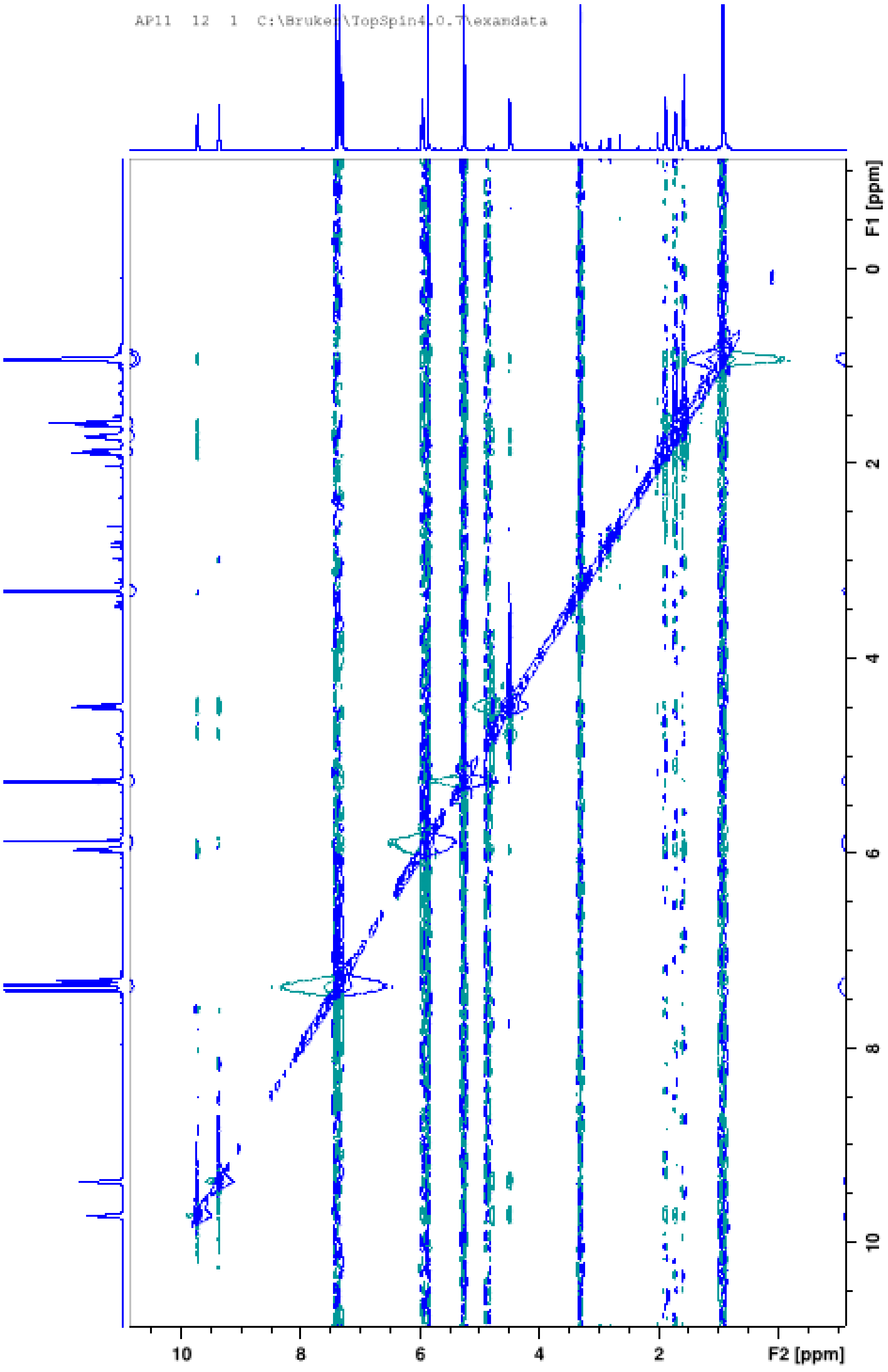

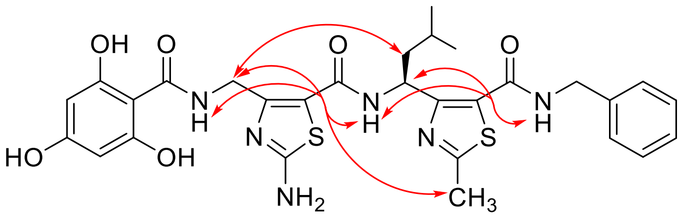

Figure S4: ROESY spectra of **3c** in CD_3OH at 293K:

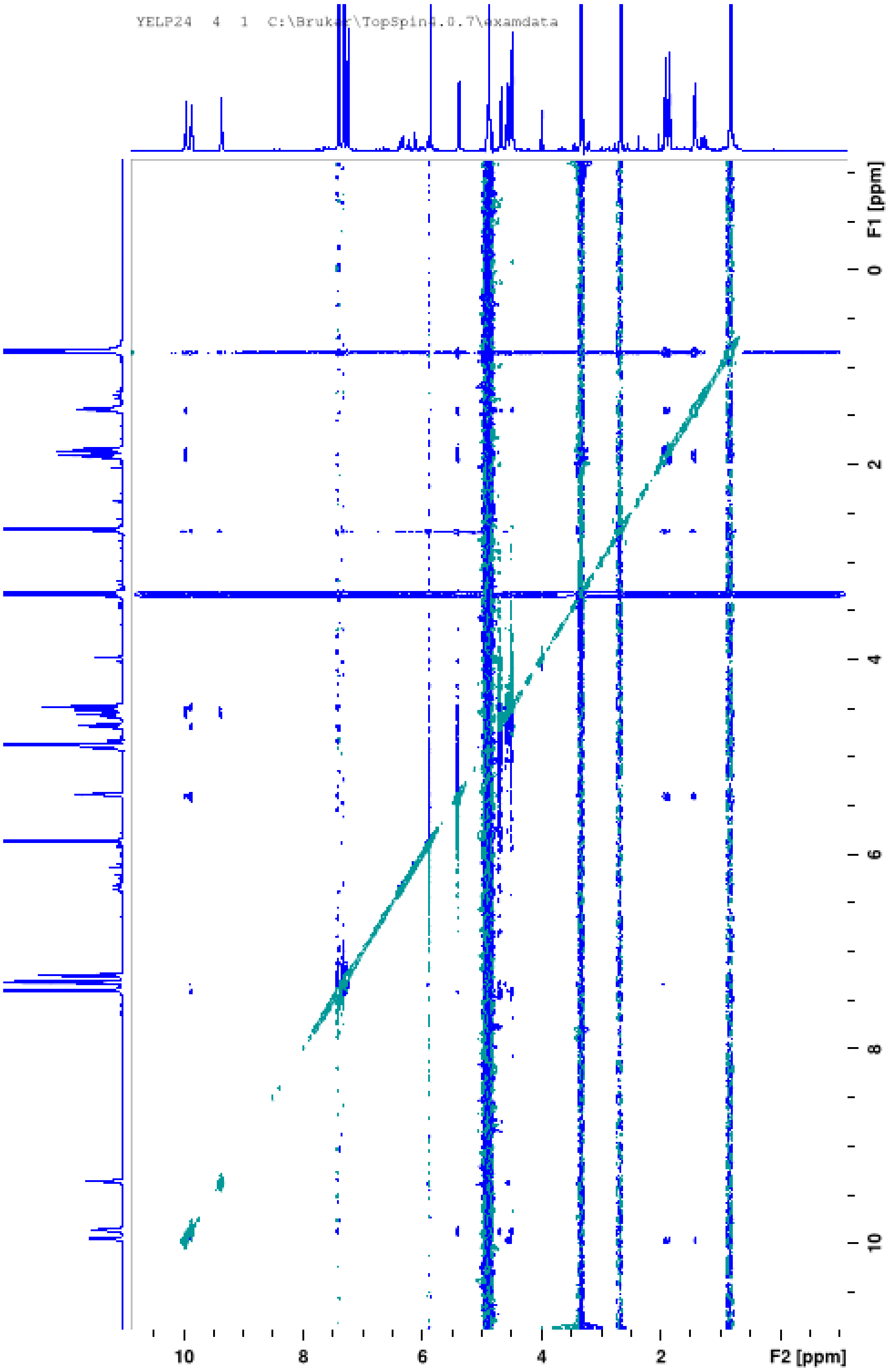

IV. REFERENCES

[1] P. A. Smith, B. C. Tripp, E. A. DiBlasio-Smith, Z. Lu, E. R. LaVallie, J. M. McCoy, *Nucleic Acids Res.* **1998**, *26*, 1414–1420.

[2] J. Viaud, M. Zeghouf, H. Barelli, J.-C. Zeeh, A. Padilla, B. Guibert, P. Chardin, C. A. Royer, J. Cherfils, A. Chavanieu, *Proc. Natl. Acad. Sci. U. S. A.* **2007**, *104*, 10370–10375.

[3] J.-C. Zeeh, M. Zeghouf, C. Grauffel, B. Guibert, E. Martin, A. Dejaegere, J. Cherfils, *J. Biol. Chem.* **2006**, *281*, 11805–11814.

[4] J. Rouhana, A. Padilla, S. Estaran, S. Bakari, S. Delbecq, Y. Boublik, J. Chopineau, M. Pugniere, A. Chavanieu, *J. Biol. Chem.* **2013**, *288*, 4659–4672.

[5] J. Rouhana, F. Hoh, S. Estaran, C. Henriquet, Y. Boublik, A. Kerkour, R. Trouillard, J. Martinez, M. Pugniere, A. Padilla, et al., *J. Med. Chem.* **2013**, *56*, 8497–8511.

[6] D. Stalder, H. Barelli, R. Gautier, E. Macia, C. L. Jackson, B. Antonny, *J. Biol. Chem.* **2011**, *286*, 3873–3883.

[7] L. Mathieu, B. Legrand, C. Deng, L. Vezenkov, E. Wenger, C. Didierjean, M. Amblard, M.-C. Averlant-Petit, N. Masurier, V. Lisowski, et al., *Angew. Chem. Int. Ed.* **2013**, *52*, 6006–6010.

[8] L. Mathieu, C. Bonnel, N. Masurier, L. T. Maillard, J. Martinez, Vincent. Lisowski, *Eur. J. Org. Chem.* **2015**, *2015*, 2262–2270.

Liste des abréviations
CDI
CH3CN
DCM
DIEA
DMAP
DME
ESI
Et2O
EtOAc
EtOH
HCOOH
HPLC
HRMS
IR
LiHMDS
MgSO4
NaHCO3
NBS
NMR
RT
TFA
TFAA
THF
TLC
Fmoc: [(9H-fluoren-9-ylmethoxy)carbonyl]

Compound 5c

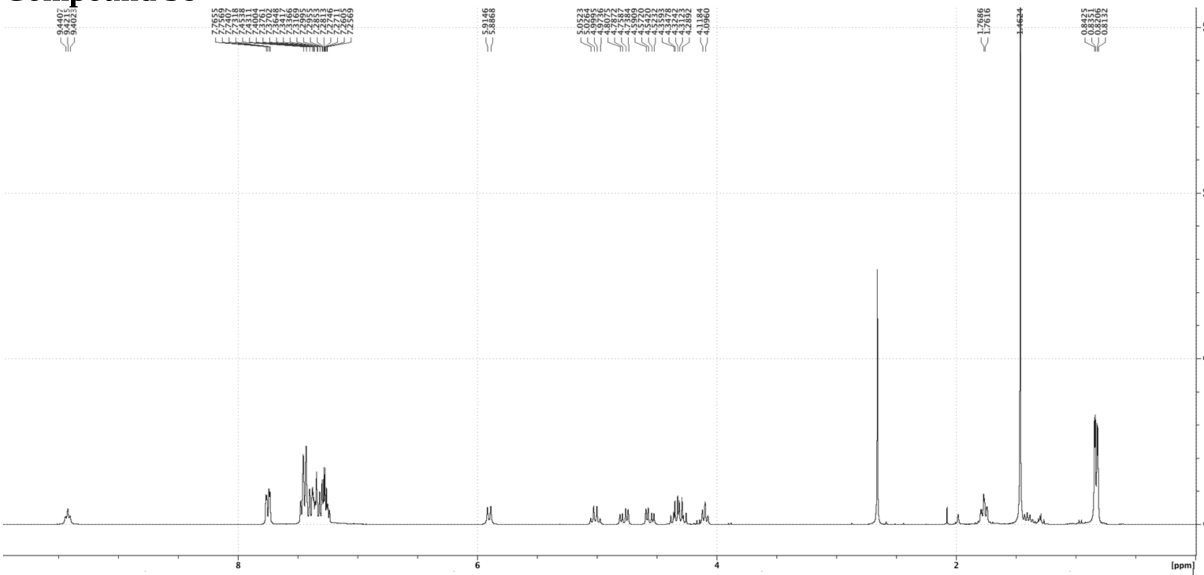

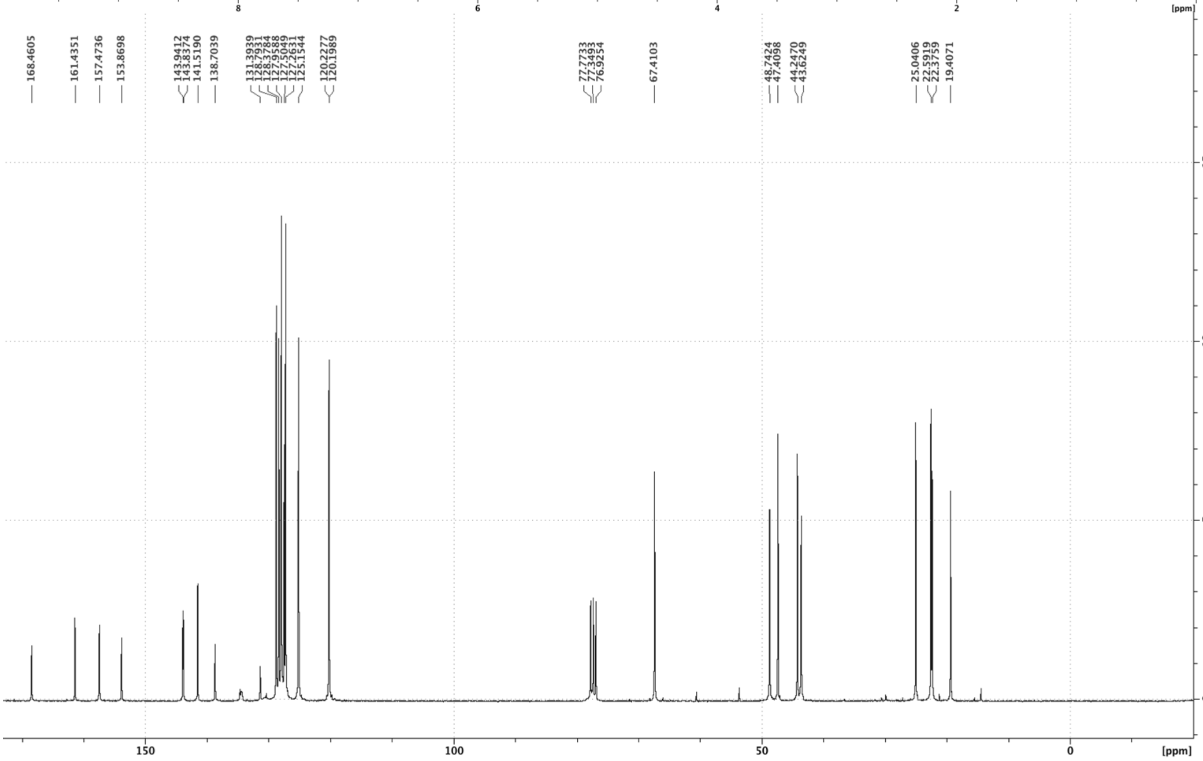

Compound 5d

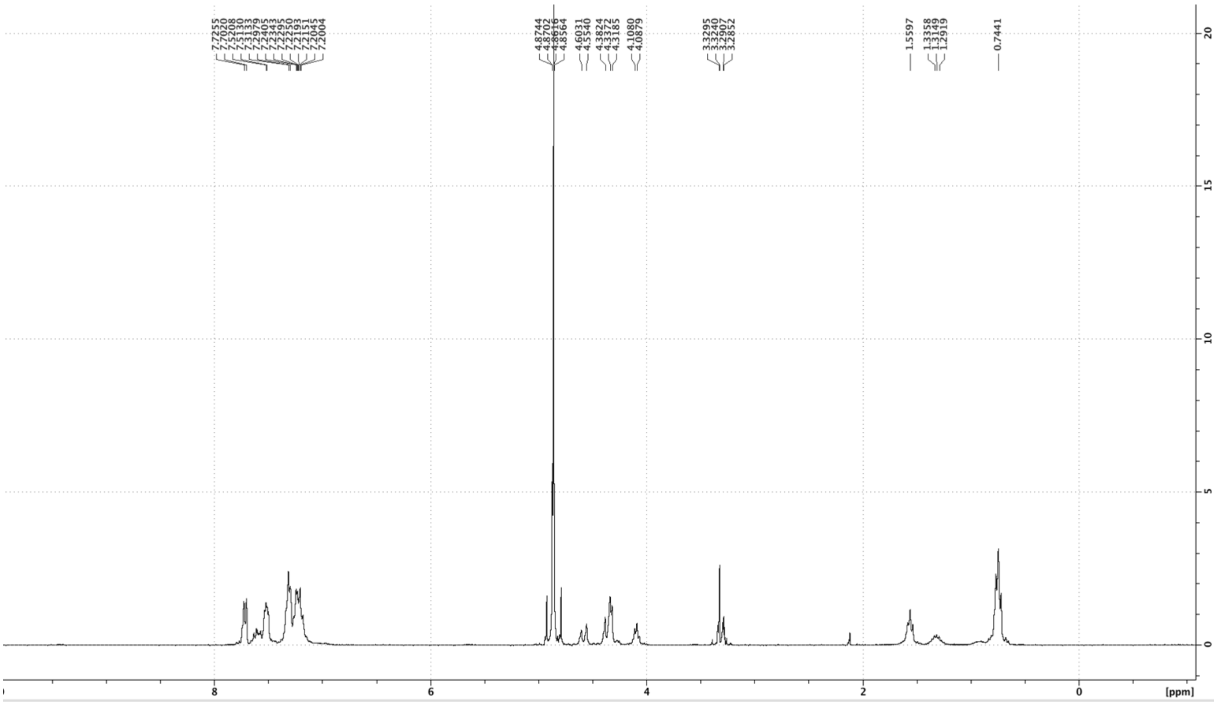

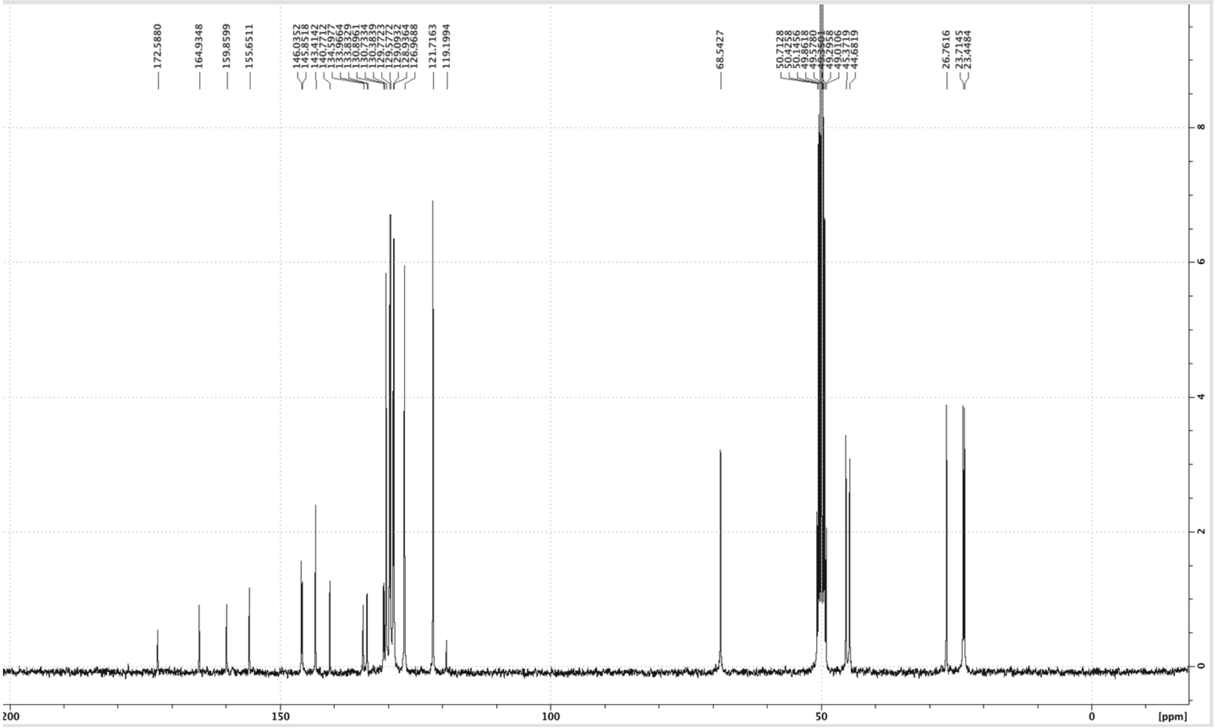

Compound 4a

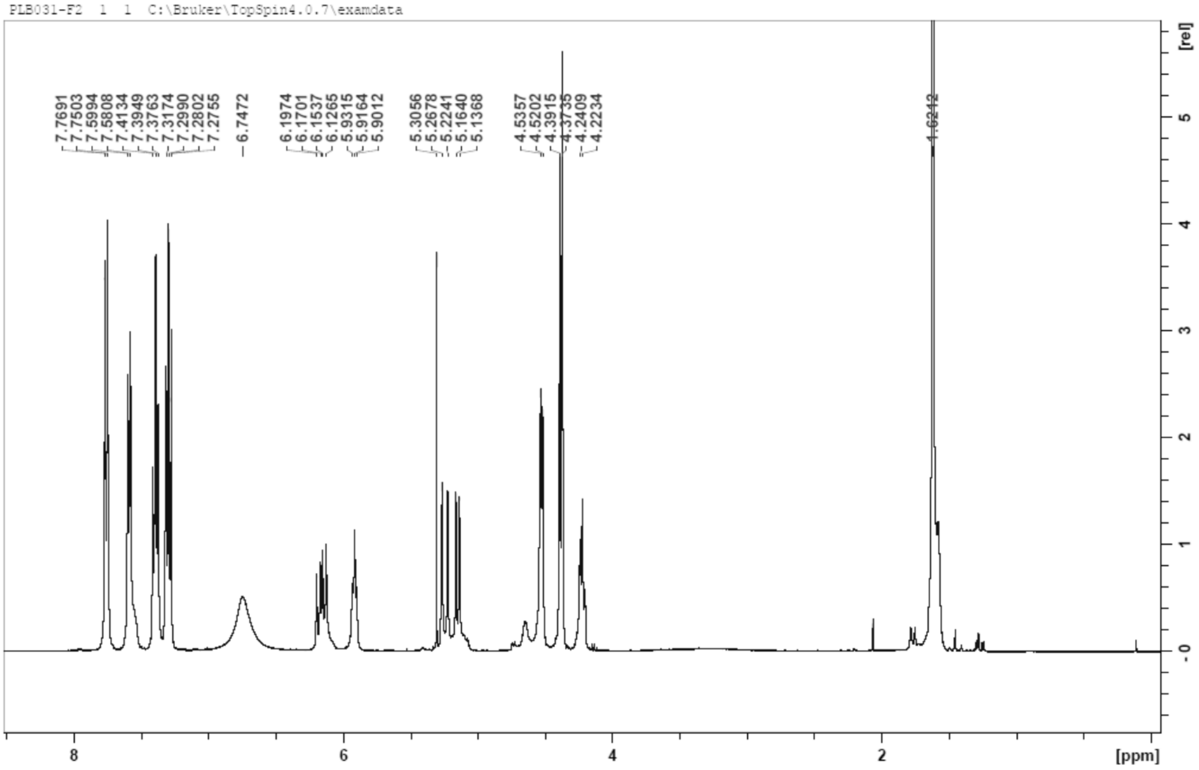
PLB031-F2 1 1 C:\Bruker\TopSpin4.0.7\examdata
7.7691
7.7503
7.5994
7.5808
7.4134
7.3949
7.3763
7.3174
7.2990
7.2802
7.2755
6.7472
6.1974
6.1701
6.1537
6.1265
5.9315
5.9164
5.9012
5.3056
5.2678
5.2241
5.1640
5.1368
4.5357
4.5202
4.3915
4.3735
4.2409
4.2234
1.6212
[rel]
[ppm]

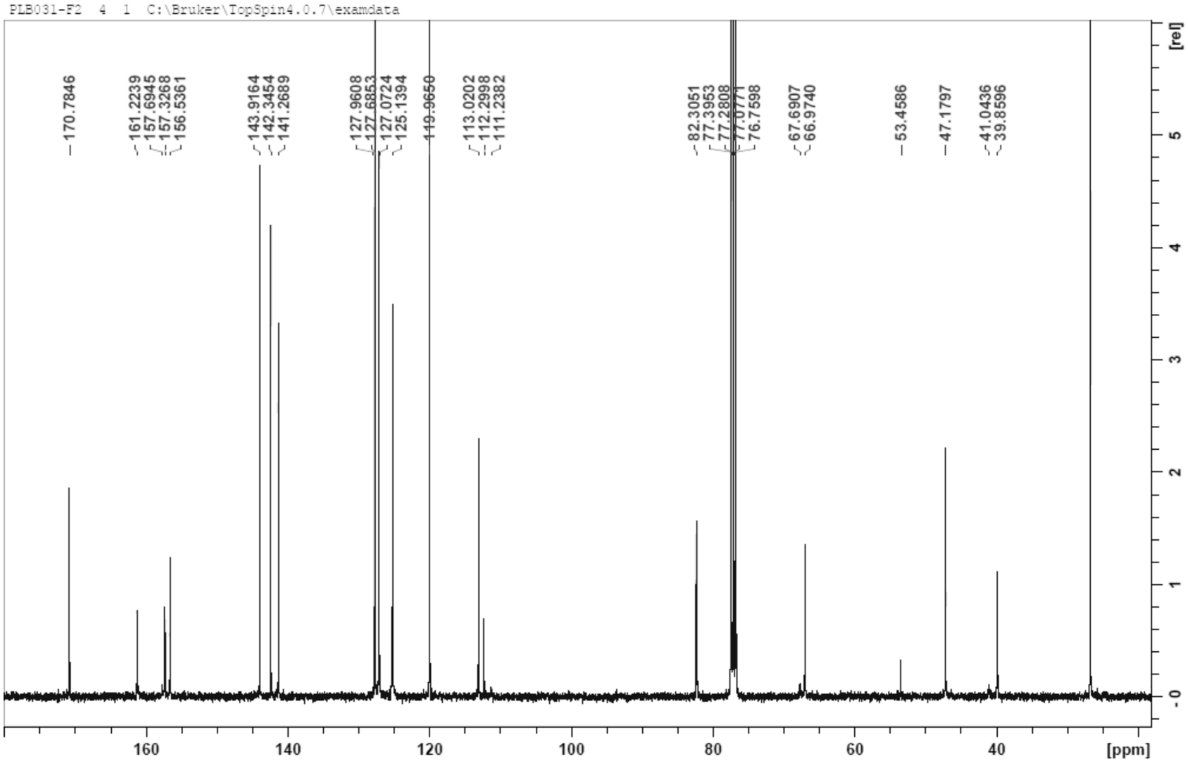
PLB031-F2 4 1 C:\Bruker\TopSpin4.0.7\examdata
170.7846
161.2239
157.6945
157.3268
156.5361
143.9164
142.3454
141.2689
127.9608
127.6853
127.0724
125.1394
119.9650
113.0202
112.2998
111.2382
82.3051
77.3953
77.2808
77.0771
76.7598
67.6907
66.9740
53.4586
47.1797
41.0436
39.8596
[rel]
[ppm]

Compound 4b

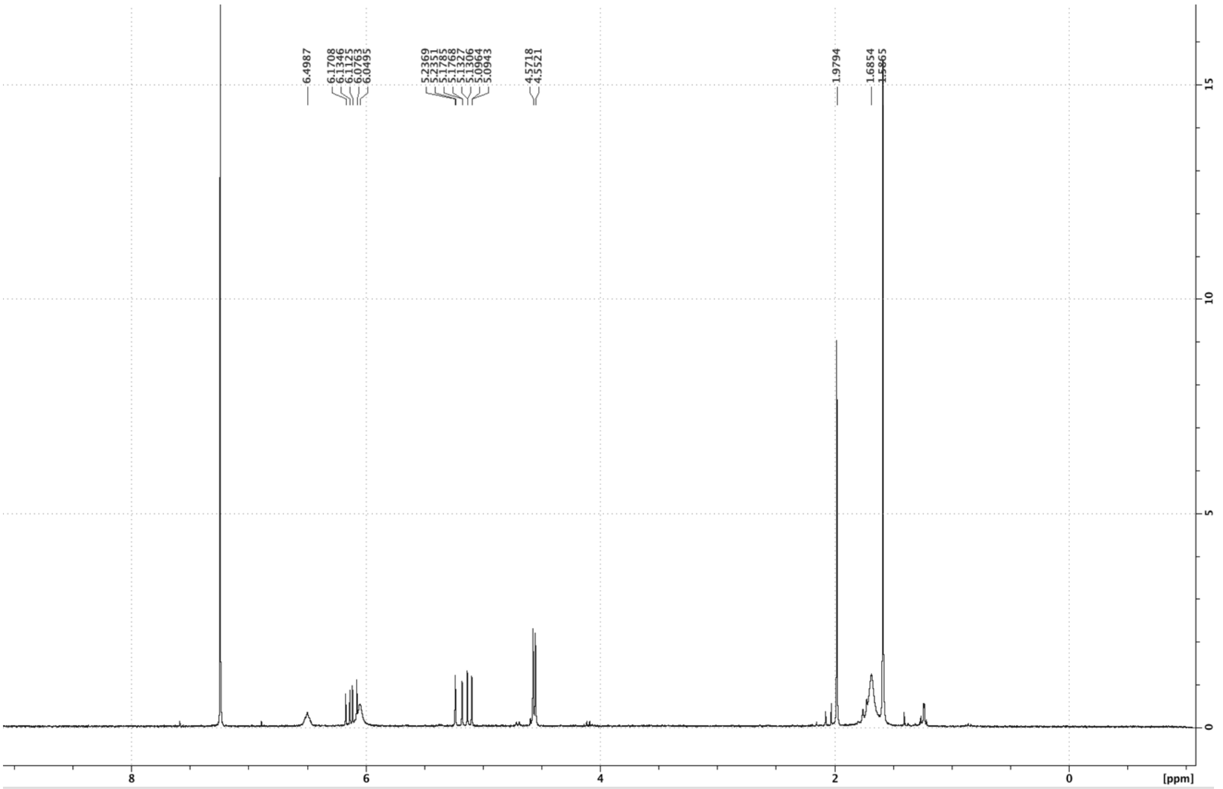
6.4987
6.1708
6.1346
6.0763
6.0495
5.2369
5.0943
4.5718
4.5521
1.9794
1.6854
[ppm]

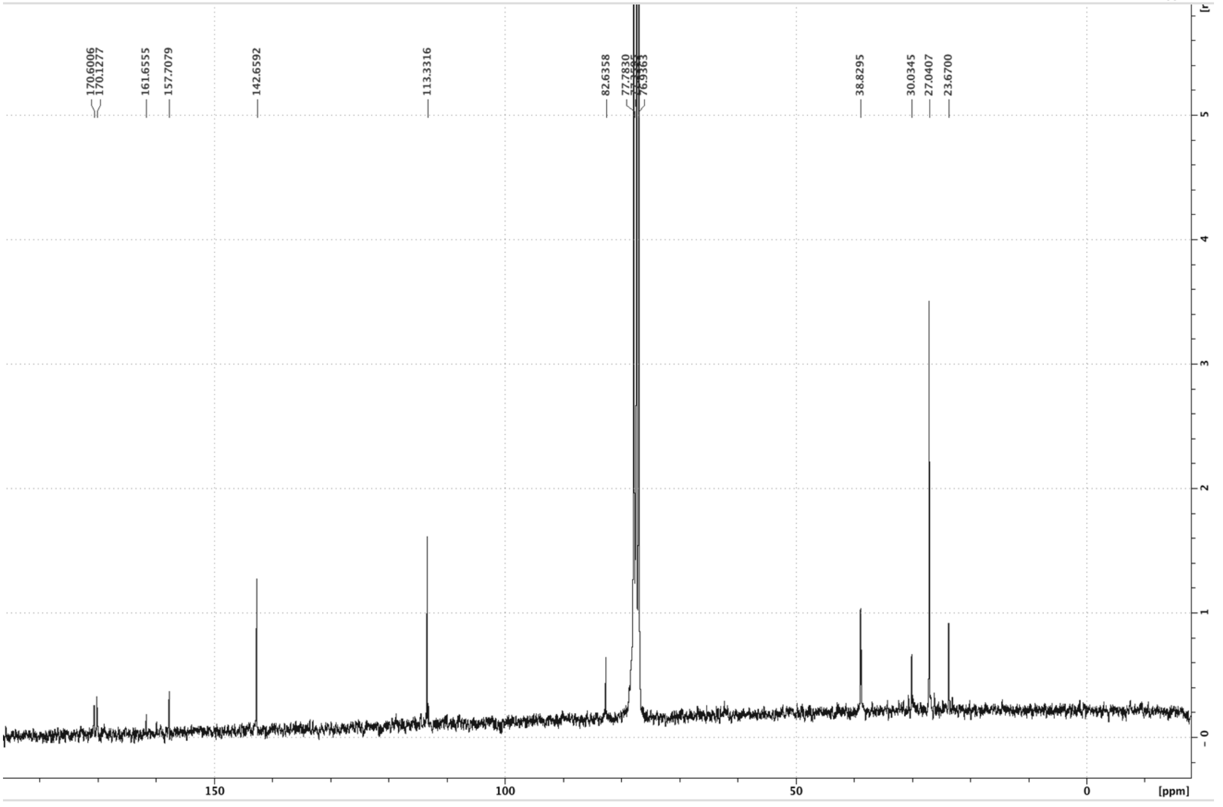
170.6006
170.1277
161.6555
157.7079
142.6592
113.3316
82.6358
77.7830
76.9363
38.8295
30.0345
27.0407
23.6700
[ppm]

Compound 1a

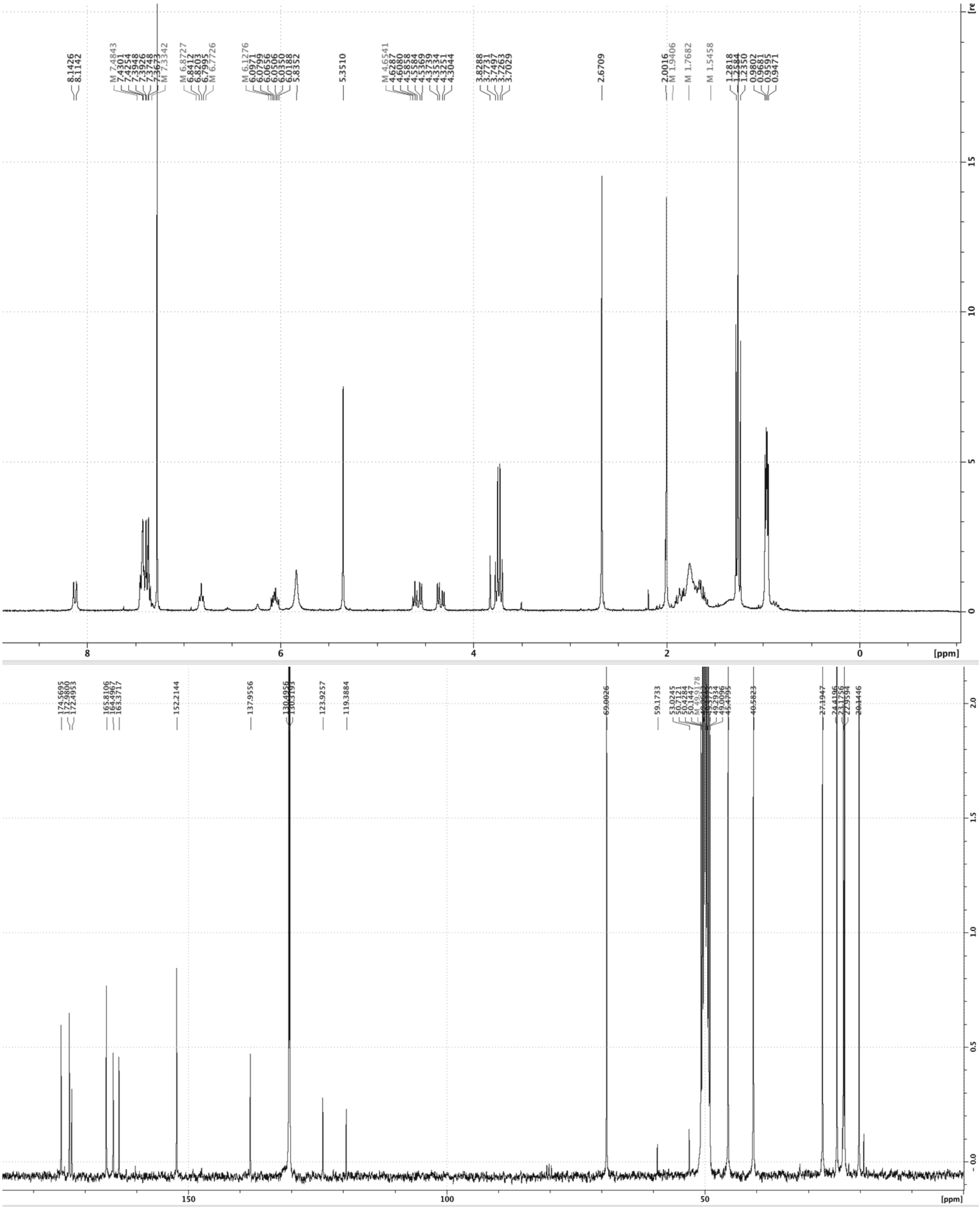

Compound 1b

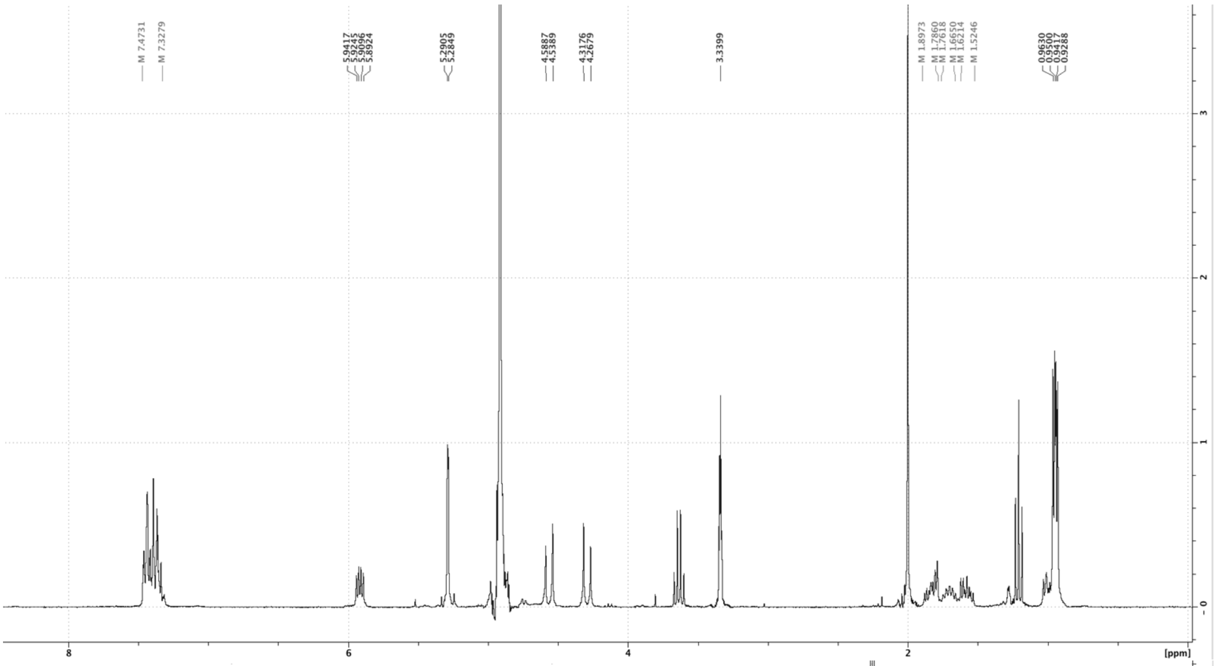
M 7.4731
M 7.3279
5.9417
5.9245
5.9096
5.8924
5.2905
5.2849
4.5887
4.5389
4.3176
4.2679
3.3399
M 1.8973
M 1.7860
M 1.7618
M 1.6650
M 1.6214
M 1.5246
0.9630
0.9500
0.9417
0.9288
[ppm]

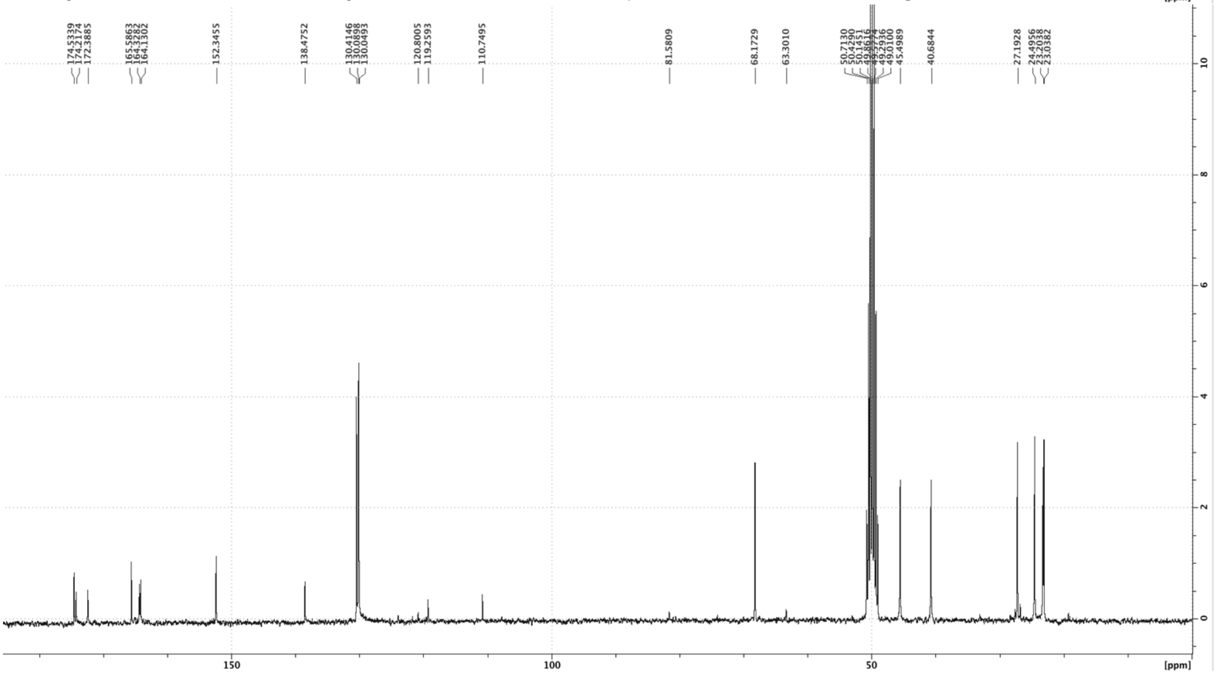
174.5339
174.2174
172.3885
165.5863
164.3282
164.1302
152.3455
138.4752
130.4146
130.0898
130.0493
120.8005
119.2593
110.7495
81.5809
68.1729
63.3010
50.7130
50.4290
50.1451
49.8616
49.5774
49.2936
49.0100
45.4989
40.6844
27.1928
24.4956
23.2038
23.0382
[ppm]

Compound 1c

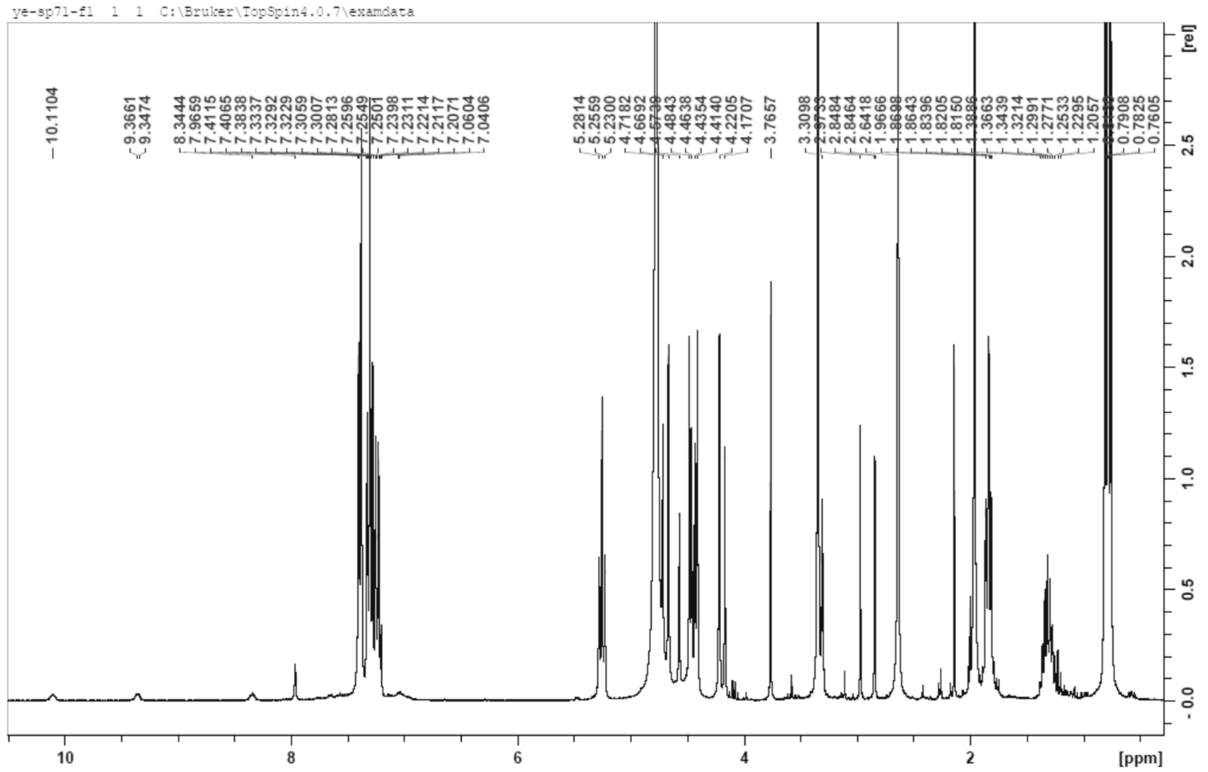

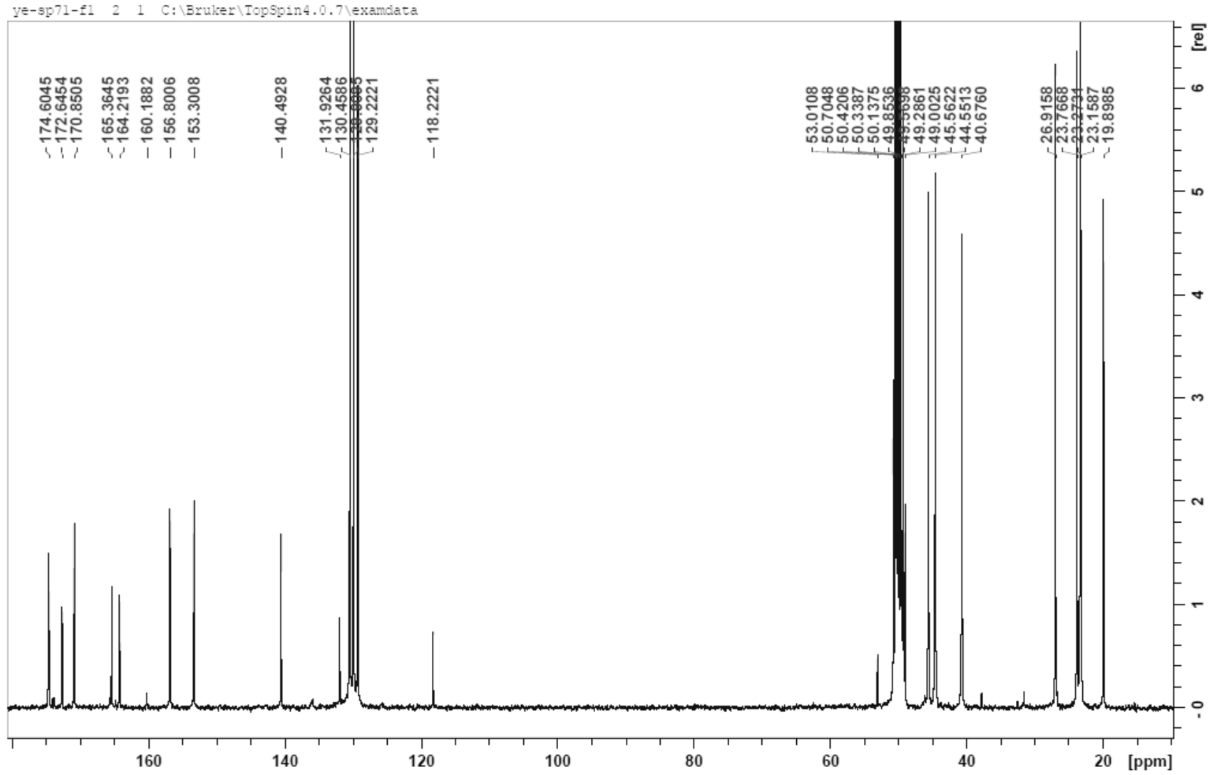

Compound 1d

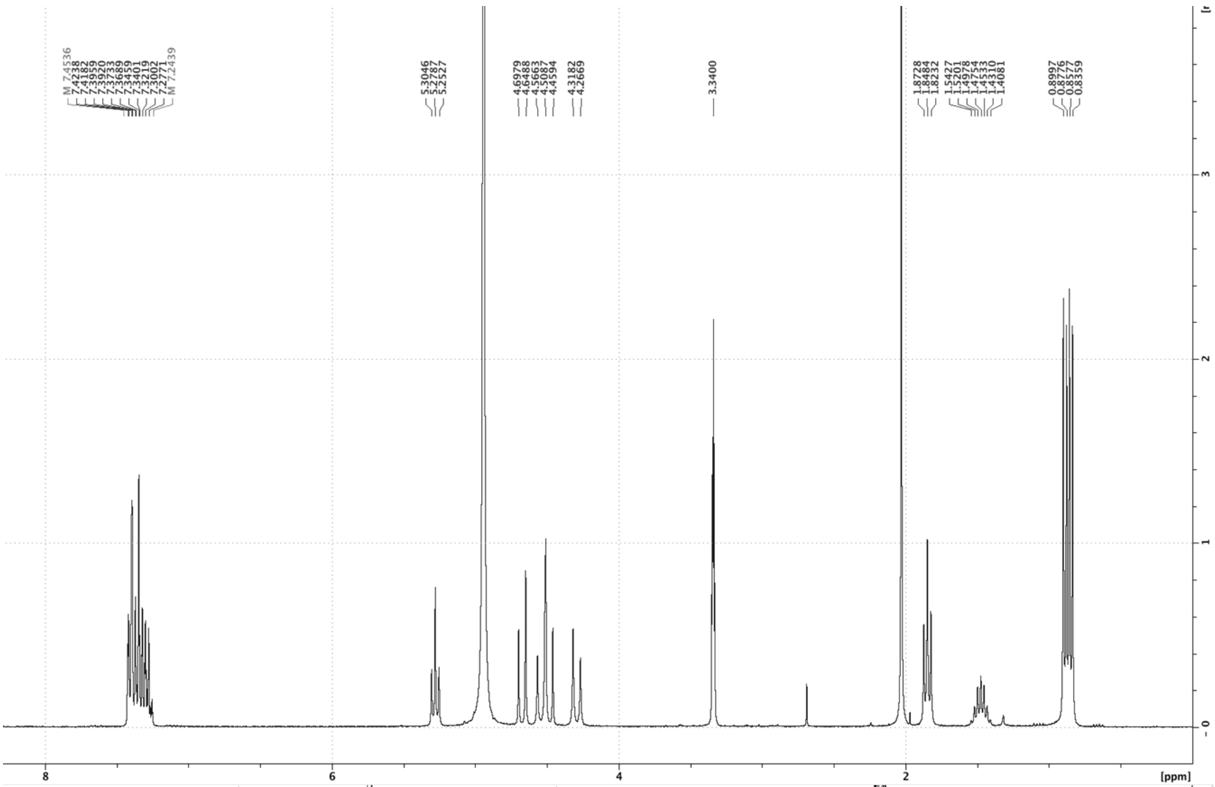

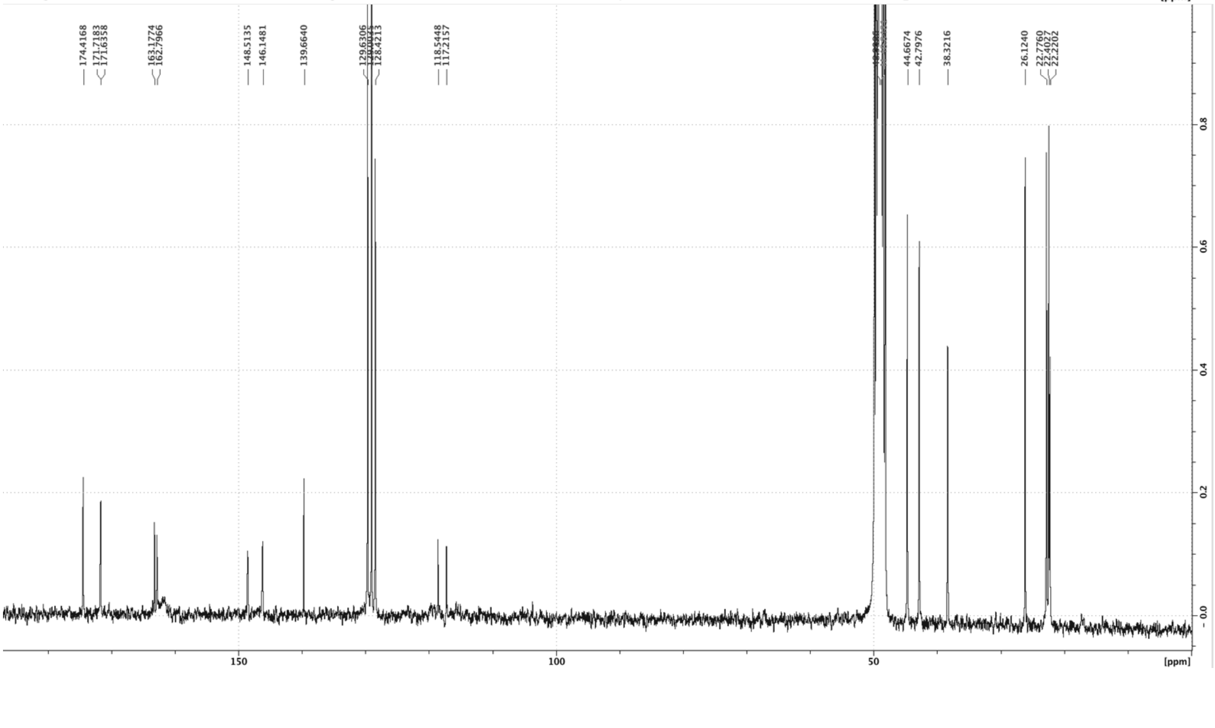

Compound 6a

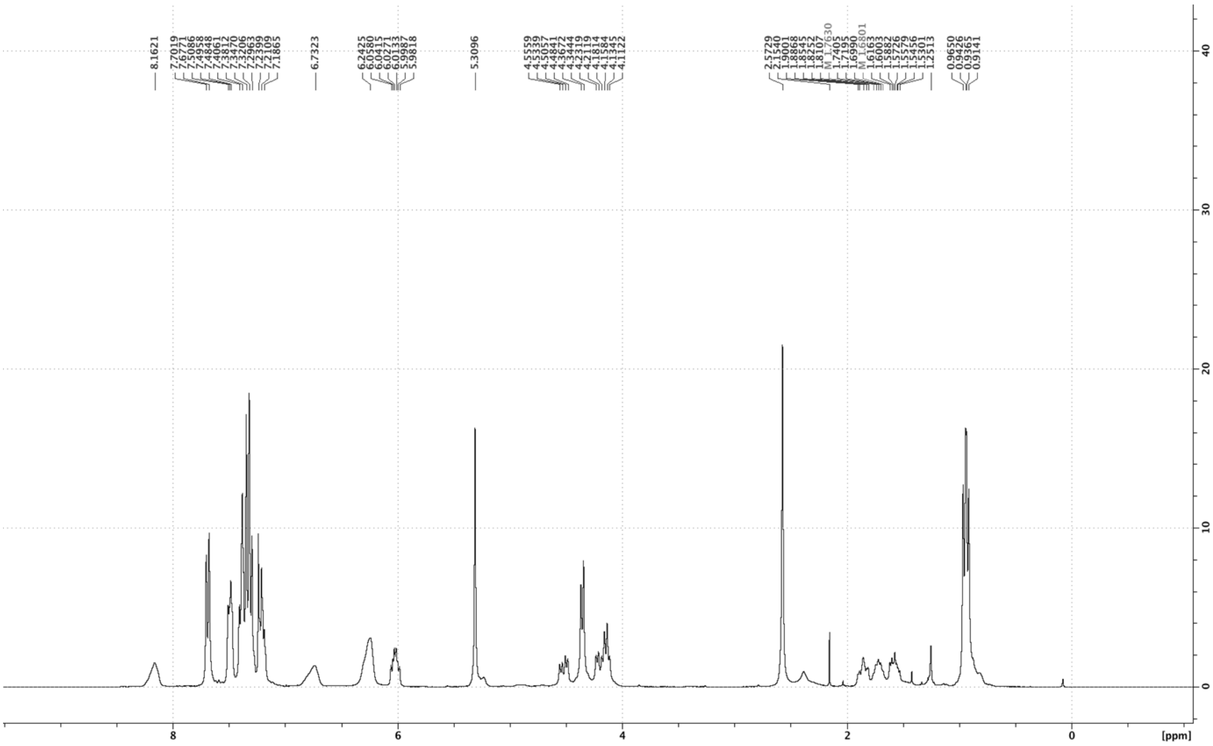

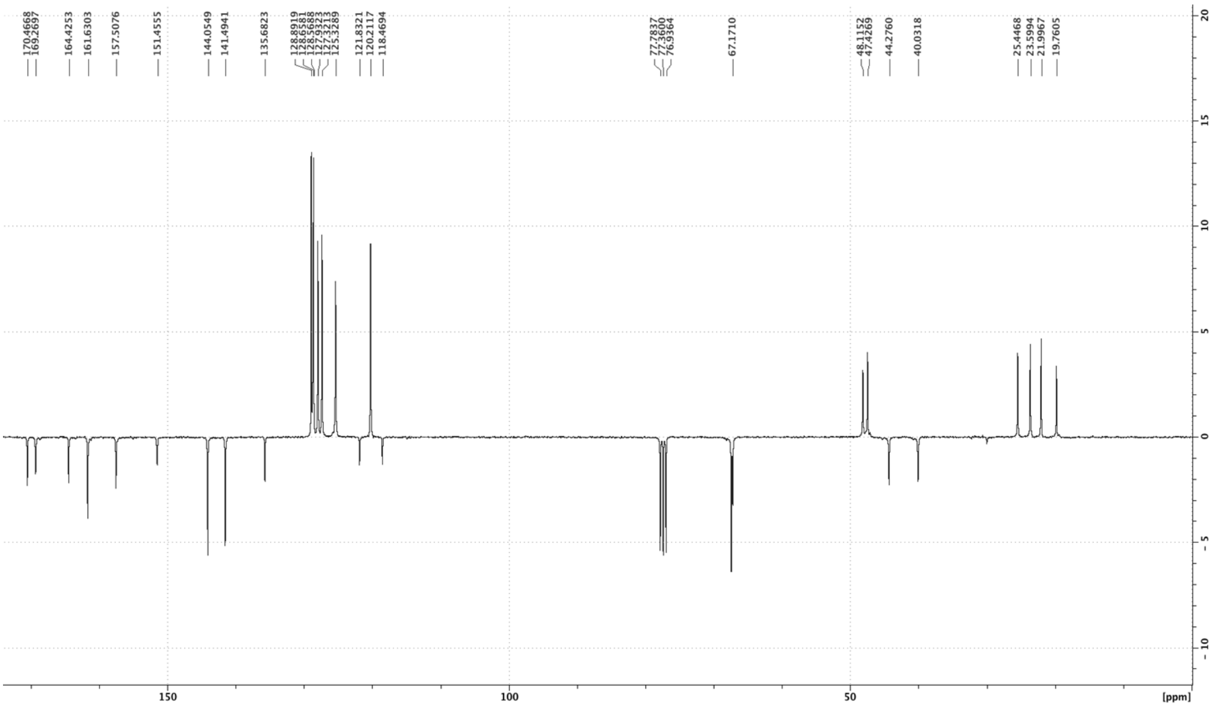

Compound 5b

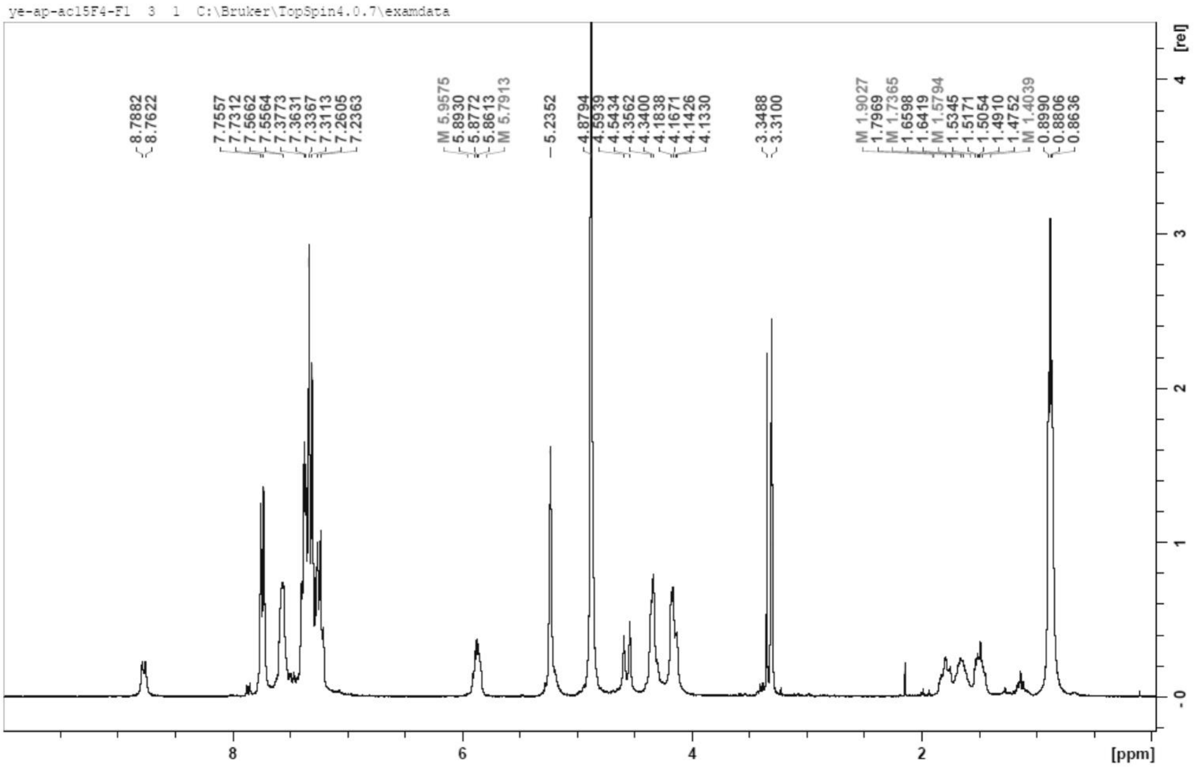
ye-ap-ac15F4-F1 3 1 C:\Bruker\TopSpin4.0.7\examdata
8.7882
8.7622
7.7557
7.7312
7.5662
7.5564
7.3773
7.3631
7.3367
7.3113
7.2605
7.2363
M 5.9575
5.8930
5.8772
5.8613
M 5.7913
5.2352
4.8794
4.5939
4.5434
4.3562
4.3400
4.1838
4.1671
4.1426
4.1330
3.3488
3.3100
M 1.9027
1.7969
M 1.7365
1.6598
1.6419
M 1.5794
1.5345
1.5171
1.5054
1.4910
1.4752
M 1.4039
0.8990
0.8806
0.8636
[rel]
[ppm]

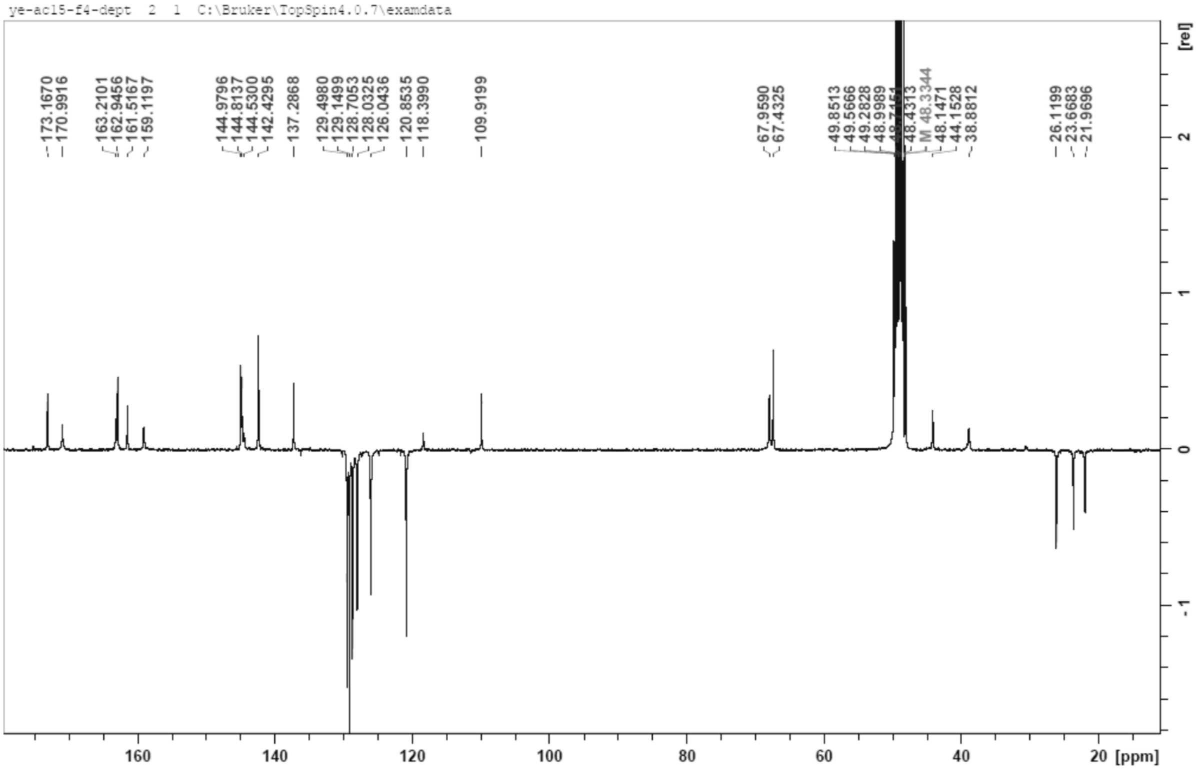
ye-ac15-f4-dept 2 1 C:\Bruker\TopSpin4.0.7\examdata
173.1670
170.9916
163.2101
162.9456
161.5167
159.1197
144.9796
144.8137
144.5300
142.4295
137.2868
129.4980
129.1499
128.7053
128.0325
126.0436
120.8535
118.3990
109.9199
67.9590
67.4325
49.8513
49.5666
49.2828
48.9989
48.4313
M 48.3344
48.1471
44.1528
38.8812
26.1199
23.6683
21.9696
[rel]
[ppm]

Compound 6c

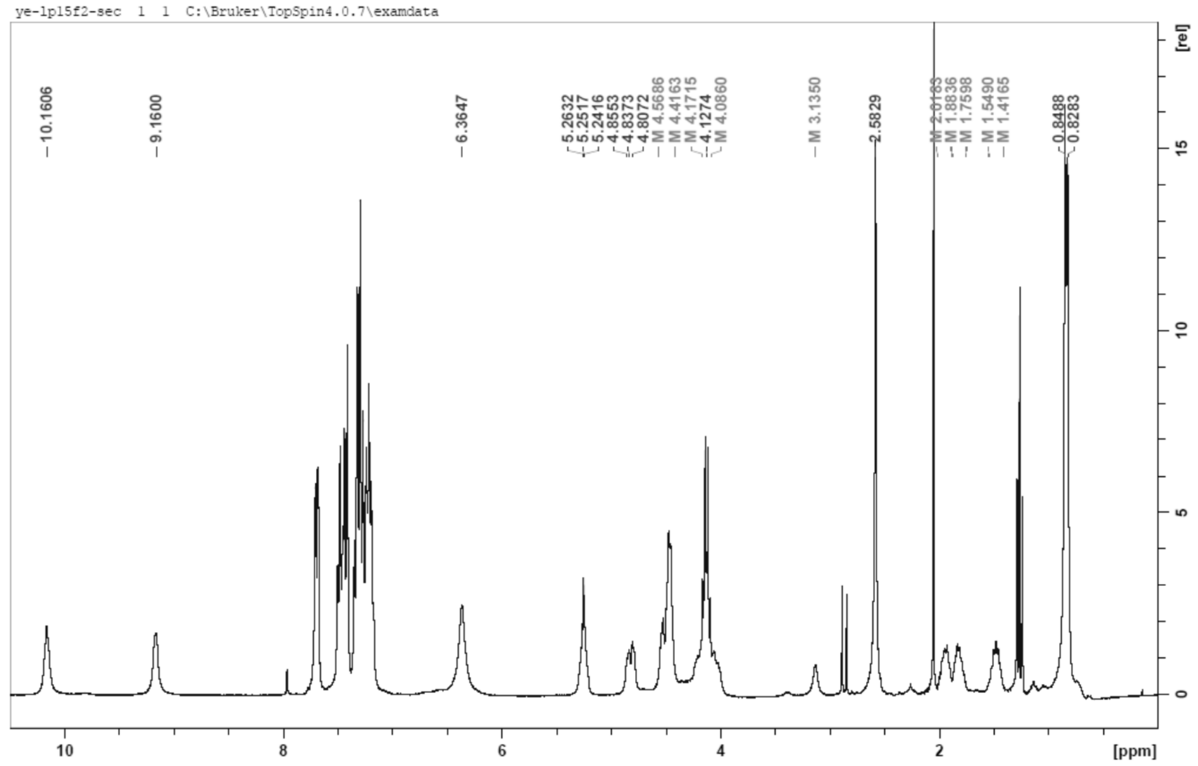
ye-1p15f2-sec 1 1 C:\Bruker\TopSpin4.0.7\examdata
[rel]
[ppm]

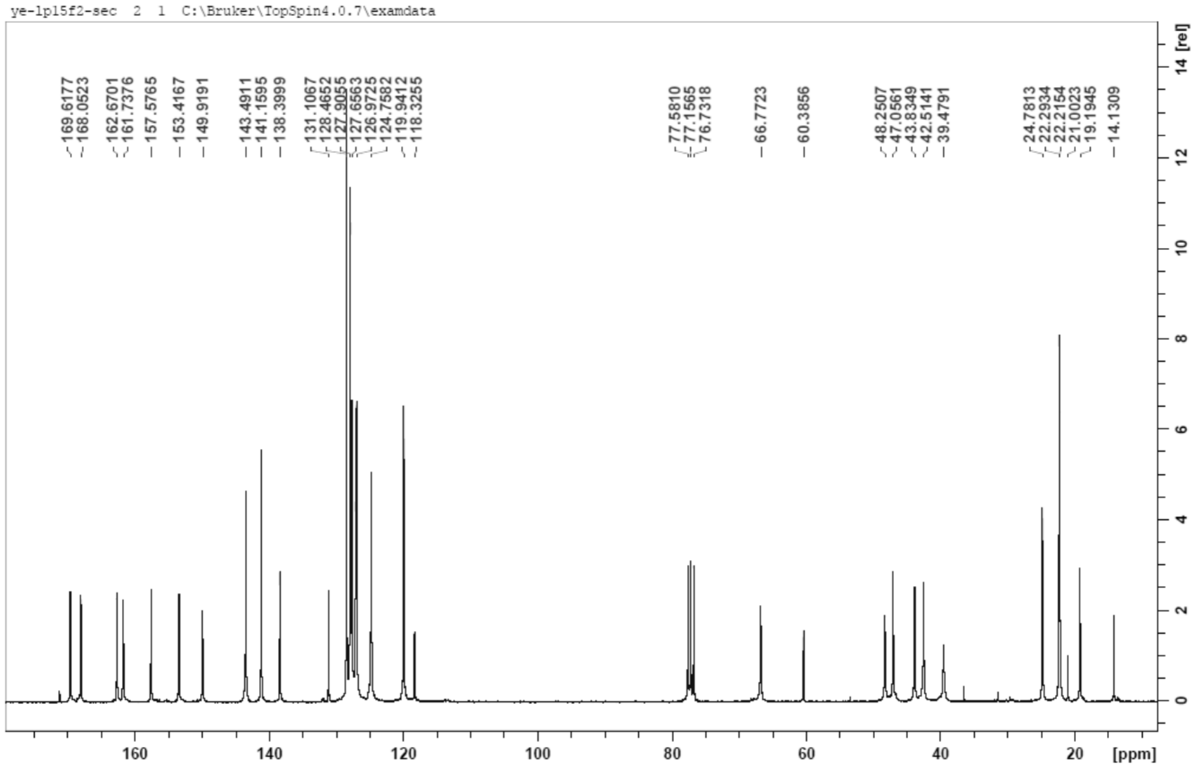
ye-1p15f2-sec 2 1 C:\Bruker\TopSpin4.0.7\examdata
[rel]
[ppm]

Compound 2a

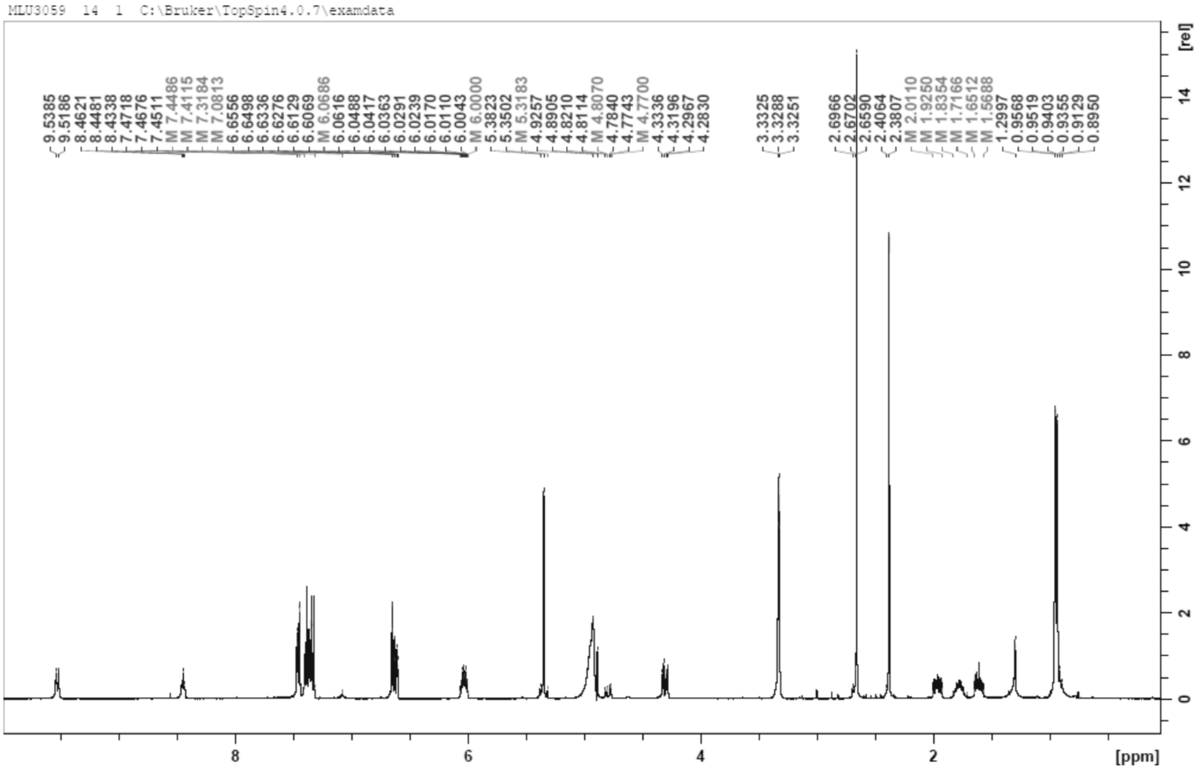

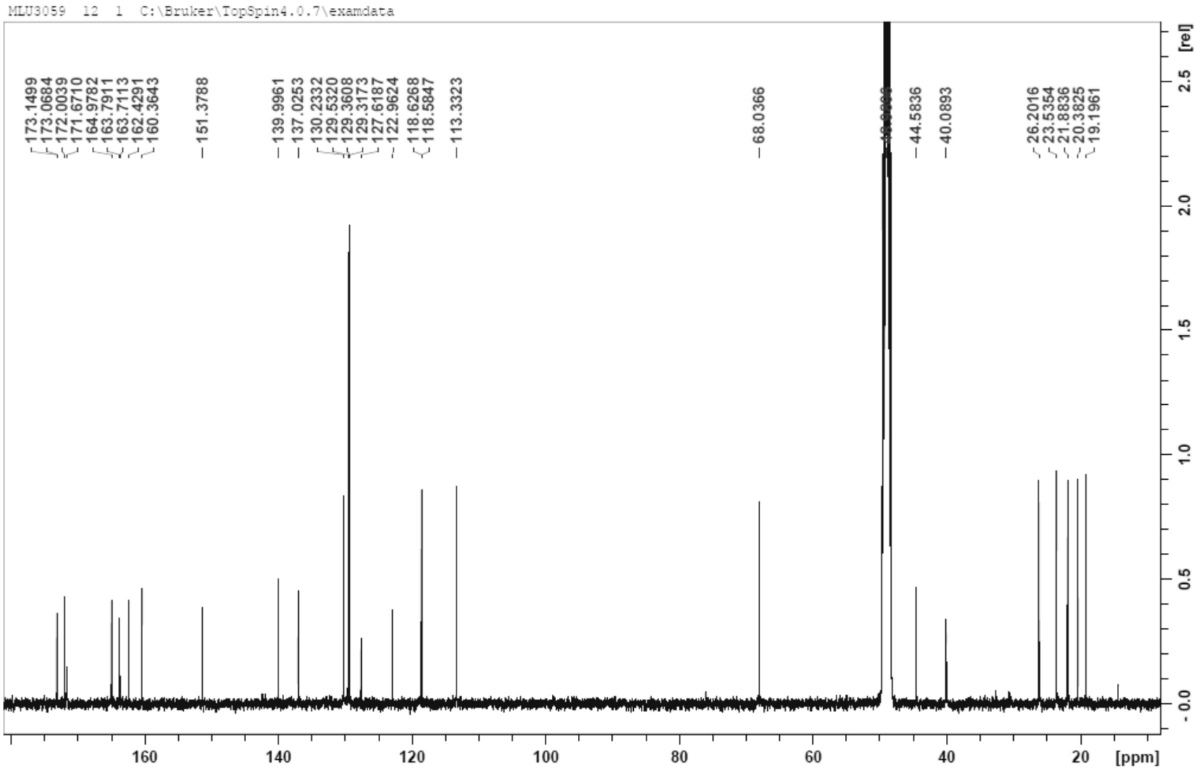

Compound 2b

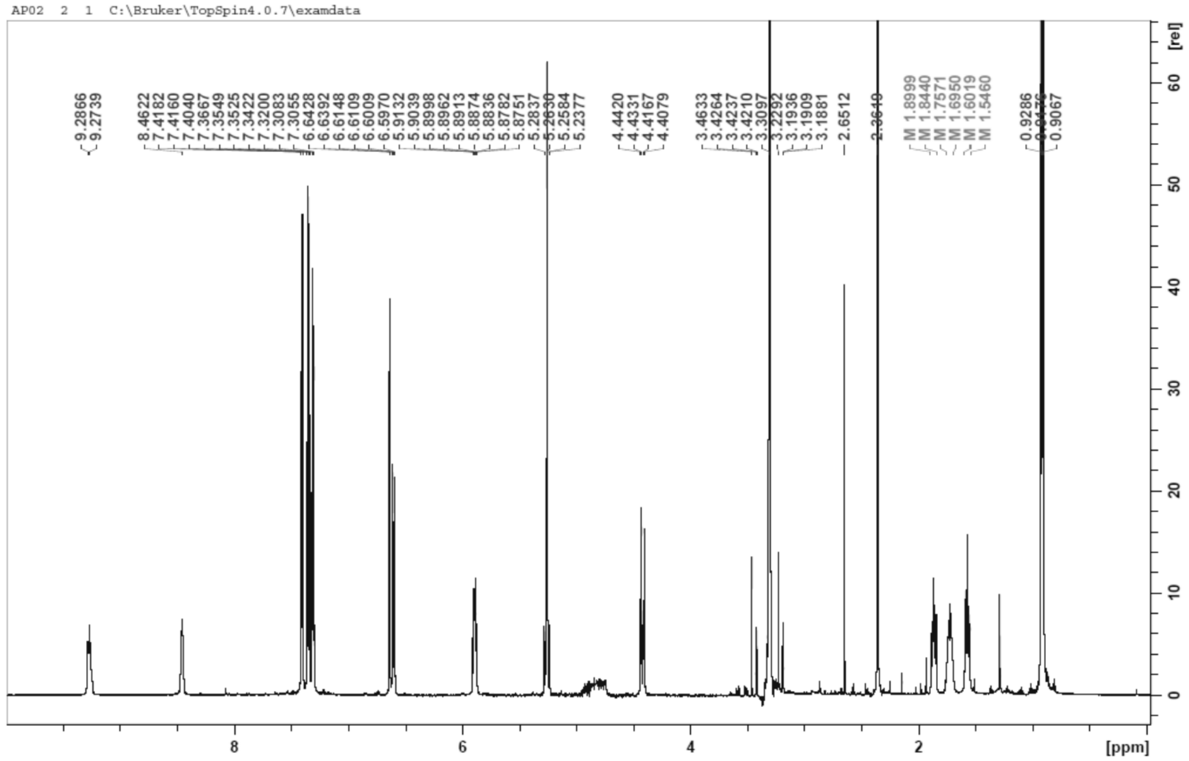
AP02 2 1 C:\Bruker\TopSpin4.0.7\examdata
9.2866
9.2739
8.4622
7.4182
7.4160
7.4040
7.3667
7.3549
7.3525
7.3422
7.3200
7.3083
7.3055
6.6428
6.6392
6.6148
6.6109
6.6009
6.5970
5.9132
5.9039
5.8998
5.8962
5.8913
5.8874
5.8836
5.8782
5.8751
5.2837
5.2630
5.2584
5.2377
4.4420
4.4331
4.4167
4.4079
3.4633
3.4264
3.4237
3.4210
3.3097
3.2292
3.1936
3.1909
3.1881
2.6512
2.3640
M 1.8999
M 1.8440
M 1.7571
M 1.6950
M 1.6019
M 1.5460
0.9286
0.9176
0.9067
[rel]
[ppm]

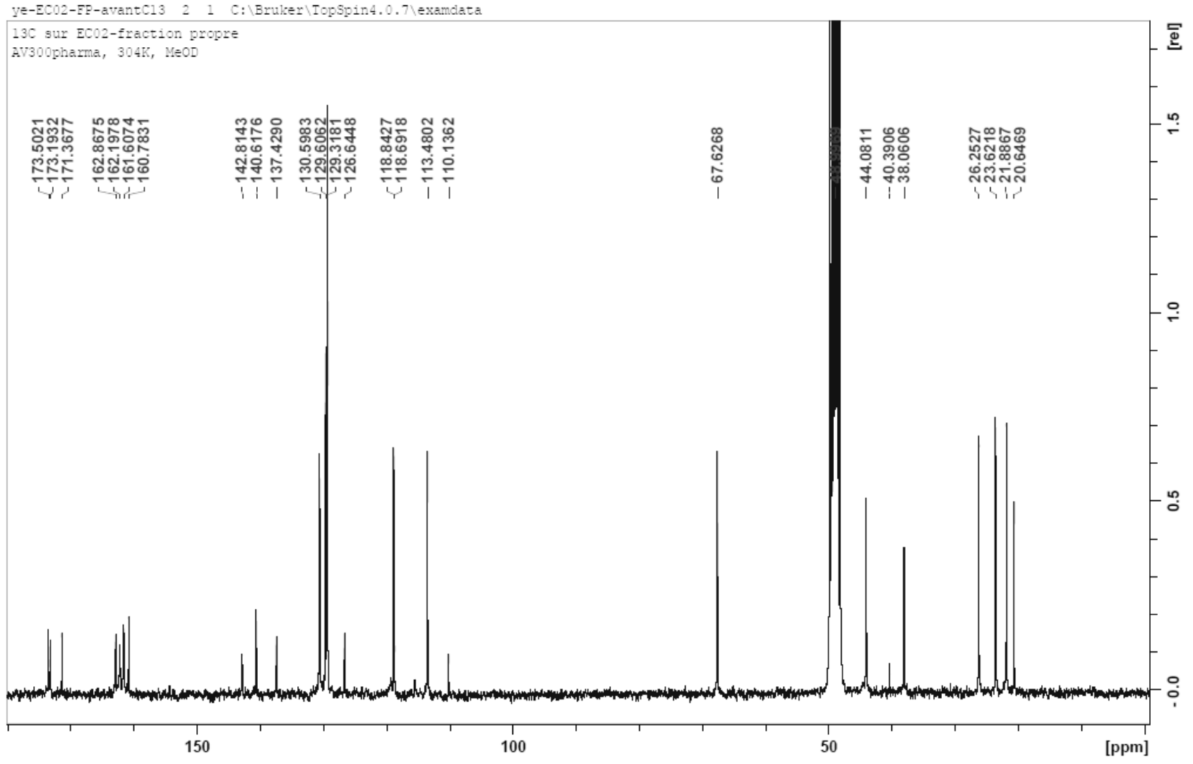
ye-EC02-FP-avantC13 2 1 C:\Bruker\TopSpin4.0.7\examdata
13C sur EC02-fraction propre
AV300pharma, 304K, MeOD
173.5021
173.1932
171.3677
162.8675
162.1978
161.6074
160.7831
142.8143
140.6176
137.4290
130.5983
129.6062
129.3181
126.6448
118.8427
118.6918
113.4802
110.1362
67.6268
44.0811
40.3906
38.0606
26.2527
23.6218
21.8867
20.6469
[rel]
[ppm]

Compound 2c

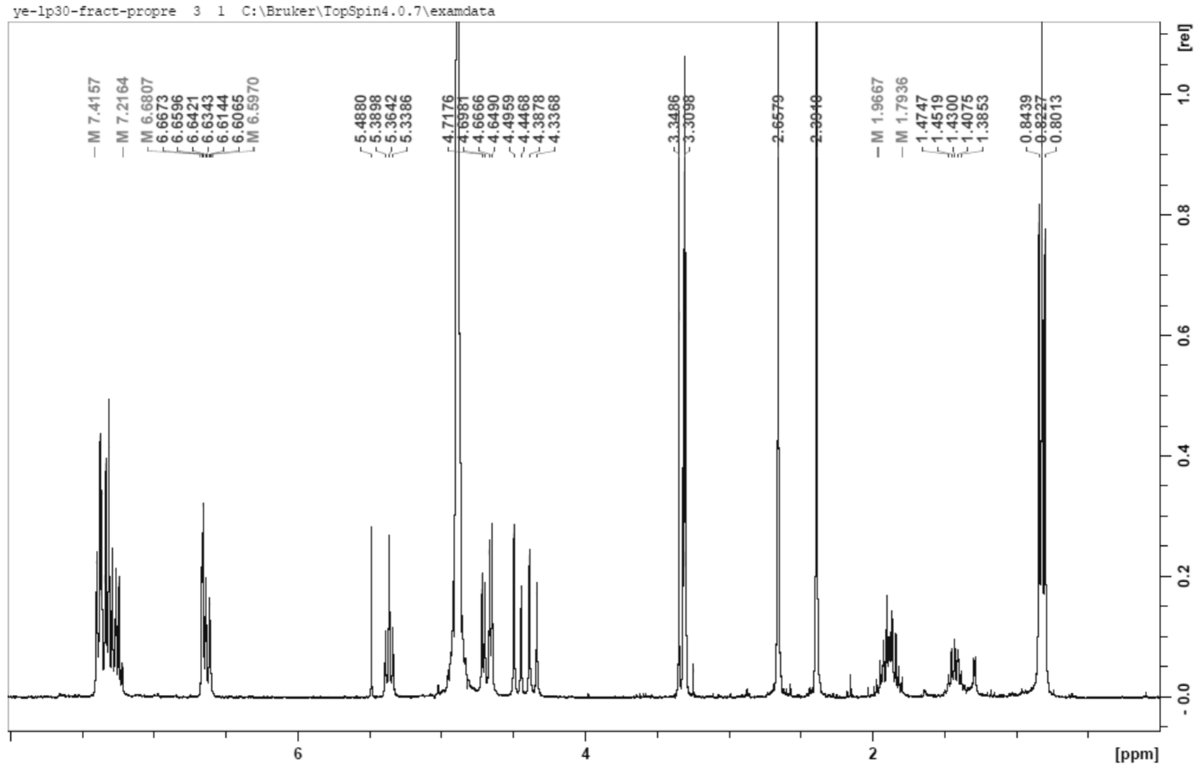
ye-lp30-fract-propre 3 1 C:\Bruker\TopSpin4.0.7\examdata
M 7.4157
M 7.2164
M 6.6807
6.6673
6.6596
6.6421
6.6343
6.6144
6.6065
M 6.5970
5.4880
5.3898
5.3642
5.3386
4.7176
4.6981
4.6666
4.6490
4.4959
4.4468
4.3878
4.3368
3.3486
3.3098
2.6579
2.3949
M 1.9667
M 1.7936
1.4747
1.4519
1.4300
1.4075
1.3853
0.8439
0.8227
0.8013
[rel]
[ppm]

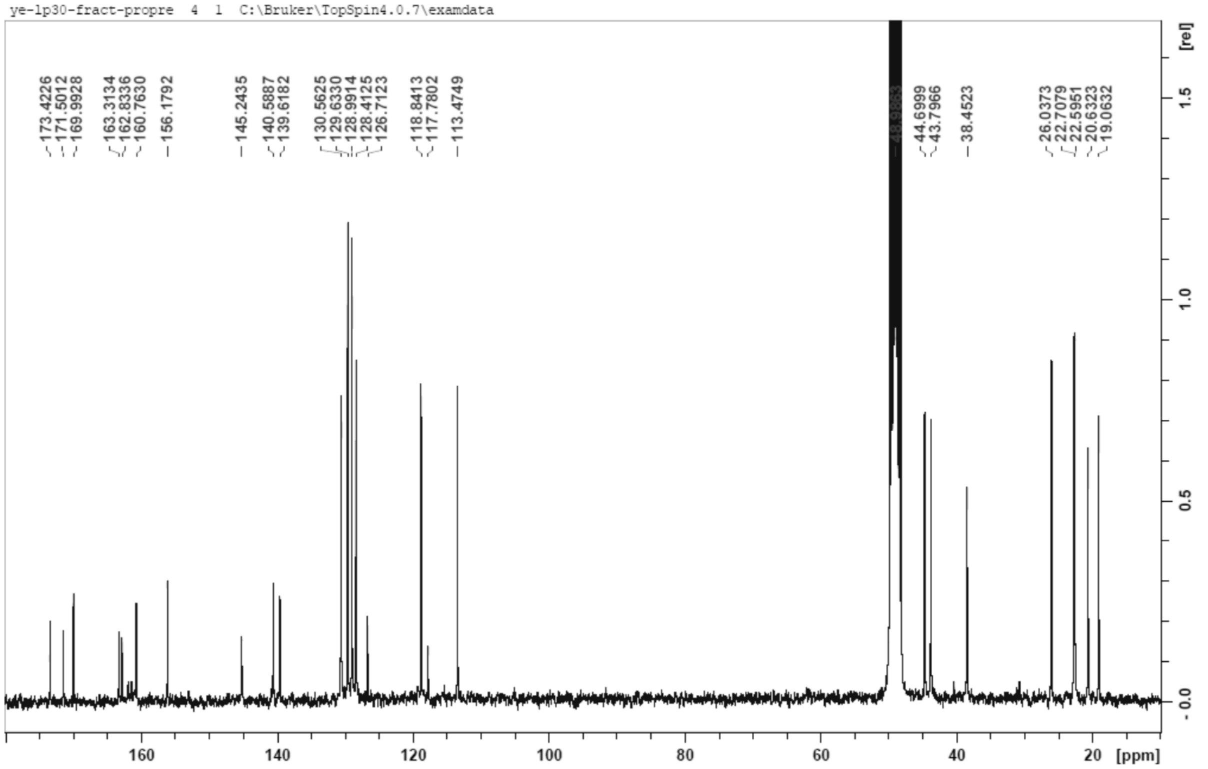
ye-lp30-fract-propre 4 1 C:\Bruker\TopSpin4.0.7\examdata
173.4226
171.5012
169.9928
163.3134
162.8336
160.7630
156.1792
145.2435
140.5887
139.6182
130.5625
129.6330
128.9914
128.4125
126.7123
118.8413
117.7802
113.4749
44.6999
43.7966
38.4523
26.0373
22.7079
22.5951
20.6323
19.0632
[rel]
[ppm]

Compound 3a

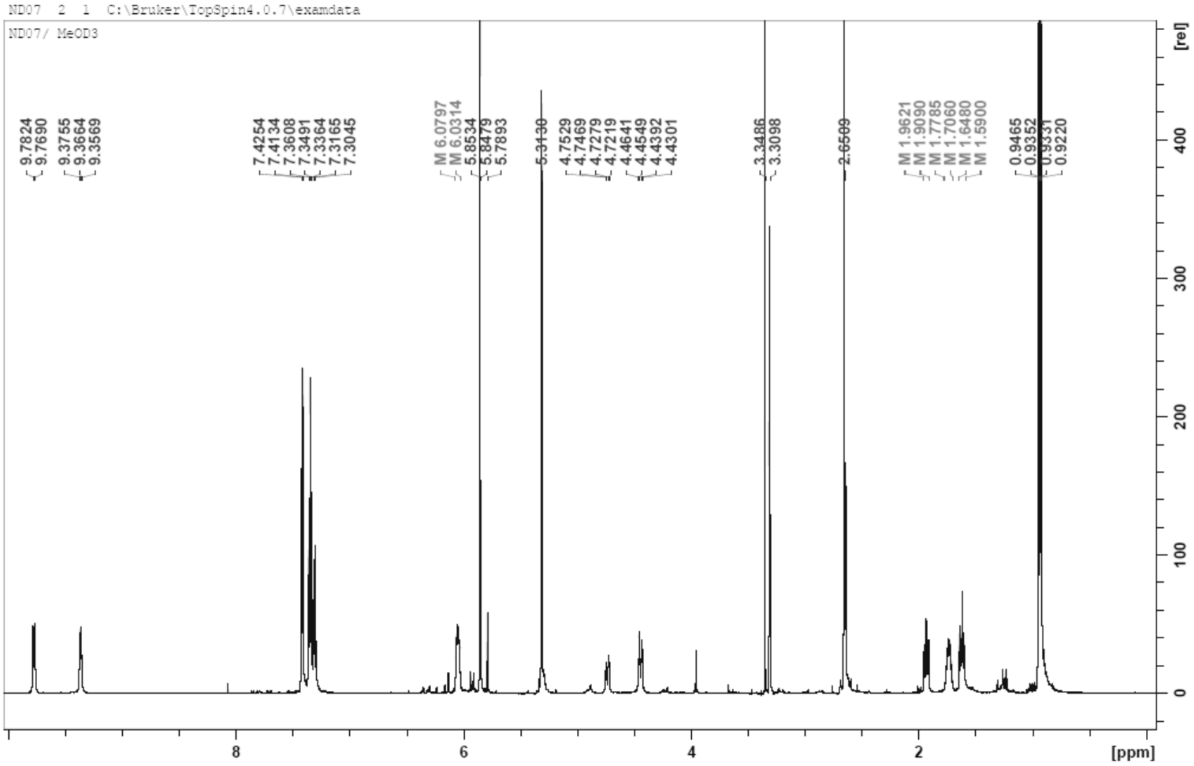
ND07 2 1 C:\Bruker\TopSpin4.0.7\examdata
ND07/ MeOD3
9.7824
9.7690
9.3755
9.3664
9.3569
7.4254
7.4134
7.3608
7.3491
7.3364
7.3165
7.3045
M 6.0797
M 6.0314
5.8534
5.8479
5.7893
5.3130
4.7529
4.7469
4.7279
4.7219
4.4641
4.4549
4.4392
4.4301
3.3486
3.3098
2.6509
M 1.9621
M 1.9090
M 1.7785
M 1.7060
M 1.6480
M 1.5900
0.9465
0.9352
0.9331
0.9220
[rel]
[ppm]

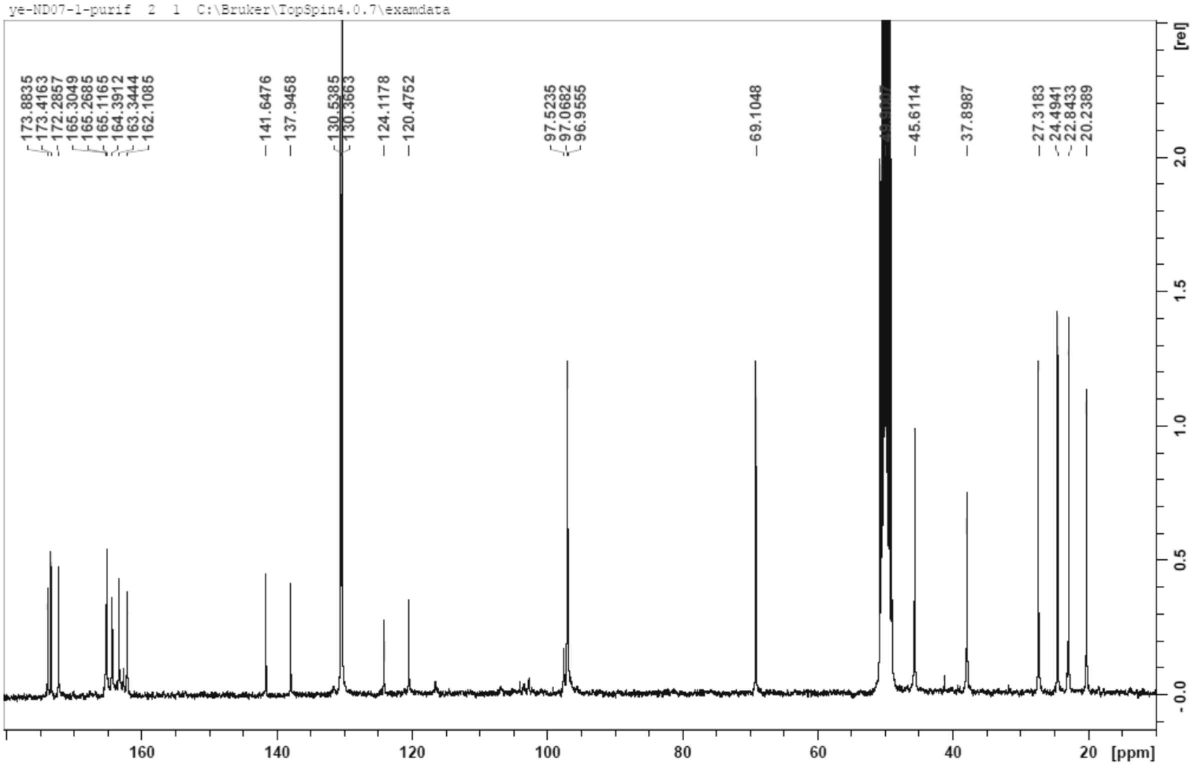
ye-ND07-1-purif 2 1 C:\Bruker\TopSpin4.0.7\examdata
173.8835
173.4163
172.2857
165.3049
165.2685
165.1165
164.3912
163.3444
162.1085
141.6476
137.9458
130.5385
130.3663
124.1178
120.4752
97.5235
97.0682
96.9555
69.1048
45.6114
37.8987
27.3183
24.4941
22.8433
20.2389
[rel]
[ppm]

Compound 3b

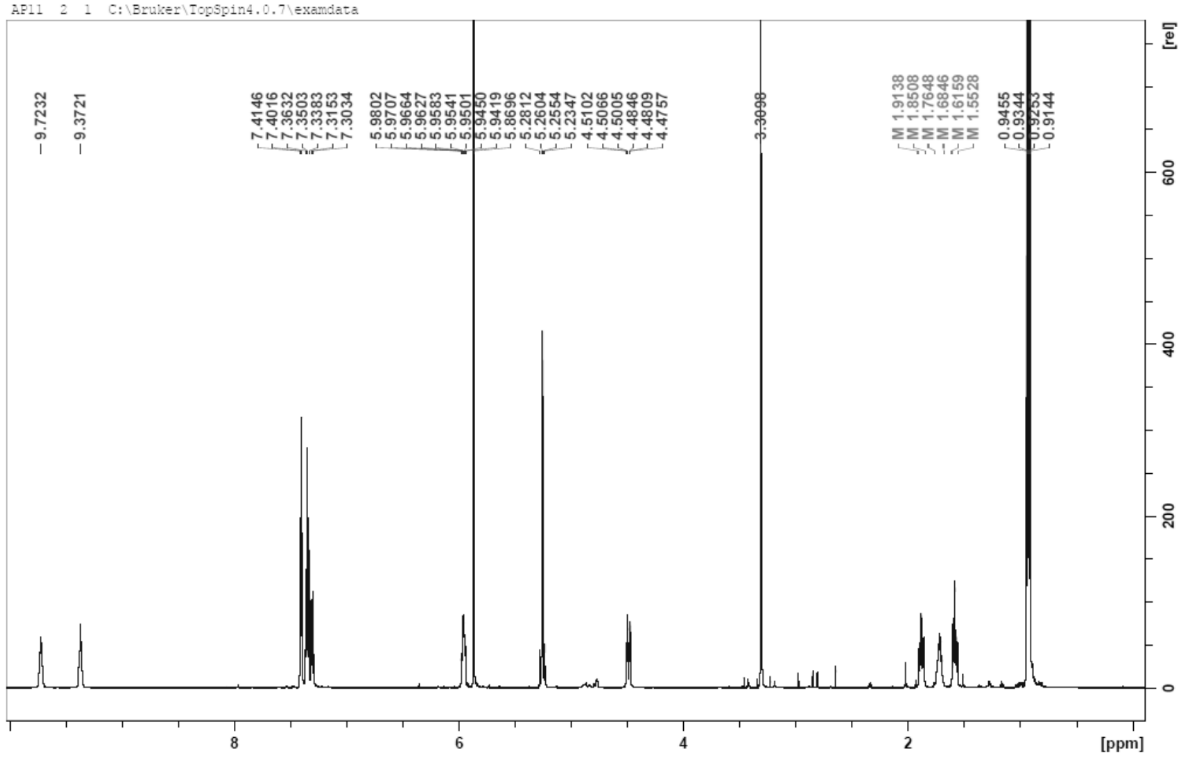
AP11 2 1 C:\Bruker\TopSpin4.0.7\examdata
[ppm]
[rel]

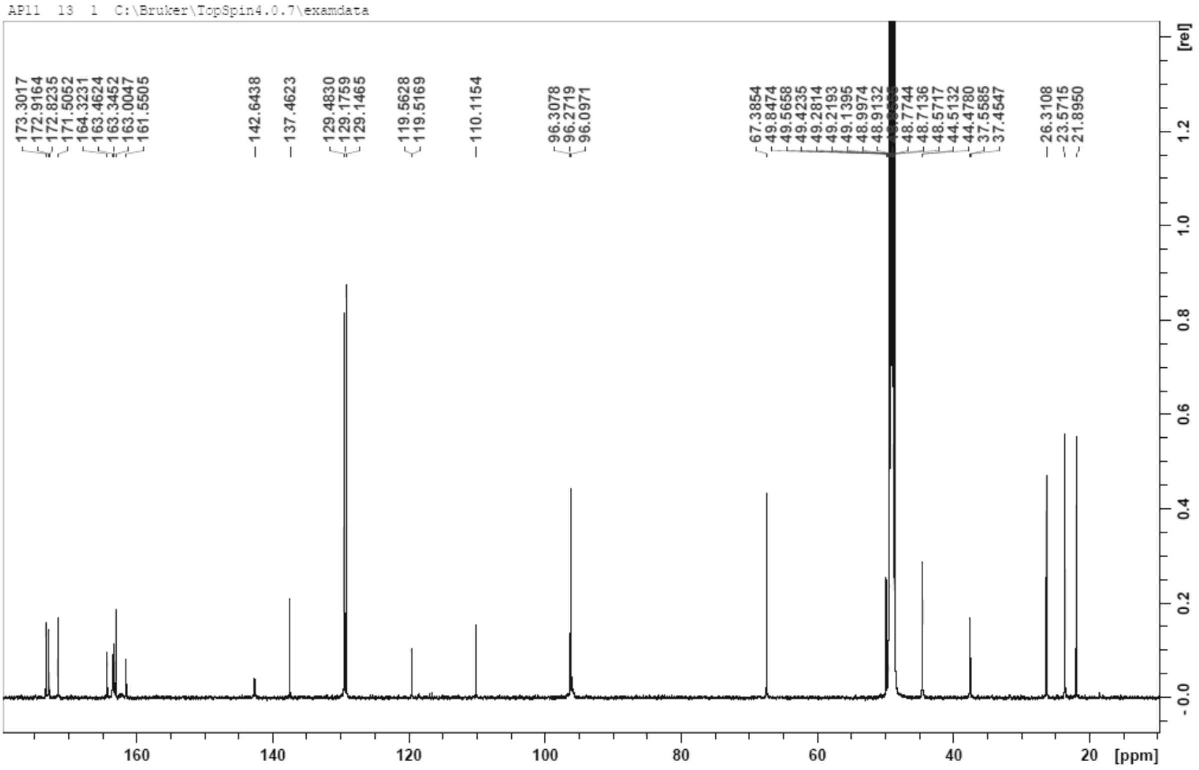
AP11 13 1 C:\Bruker\TopSpin4.0.7\examdata
[ppm]
[rel]

Compound 3c

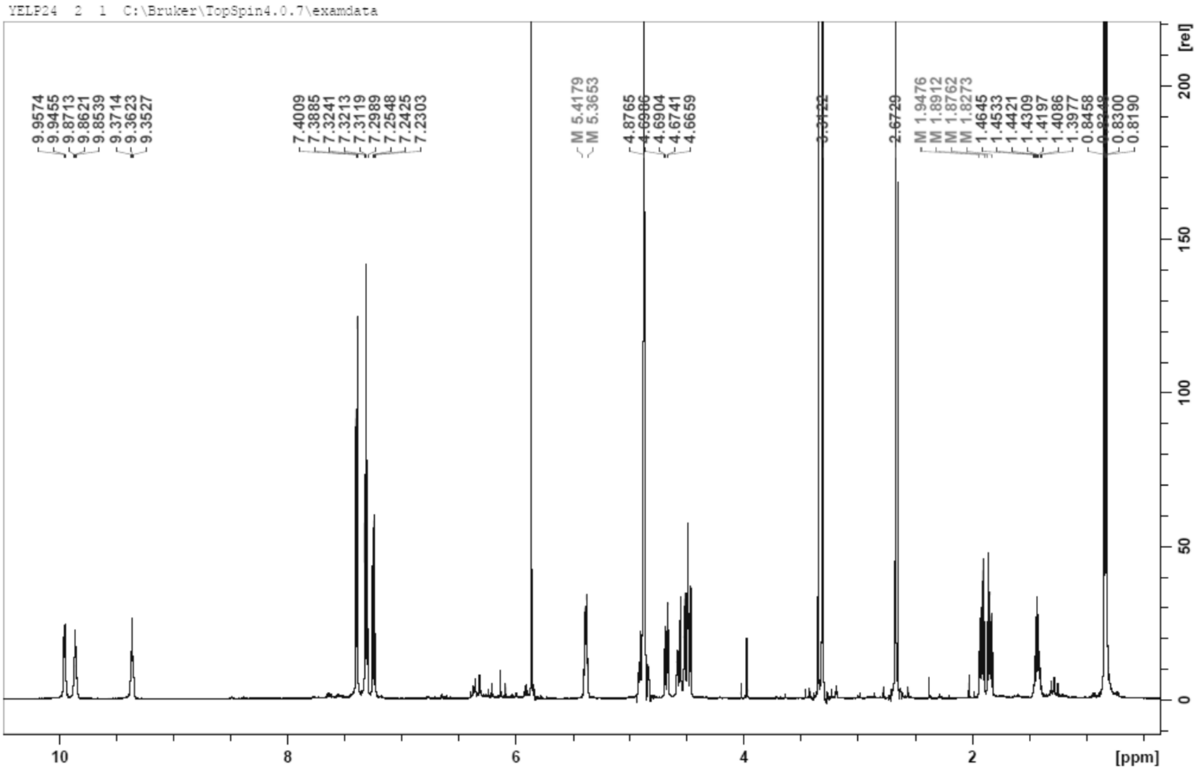

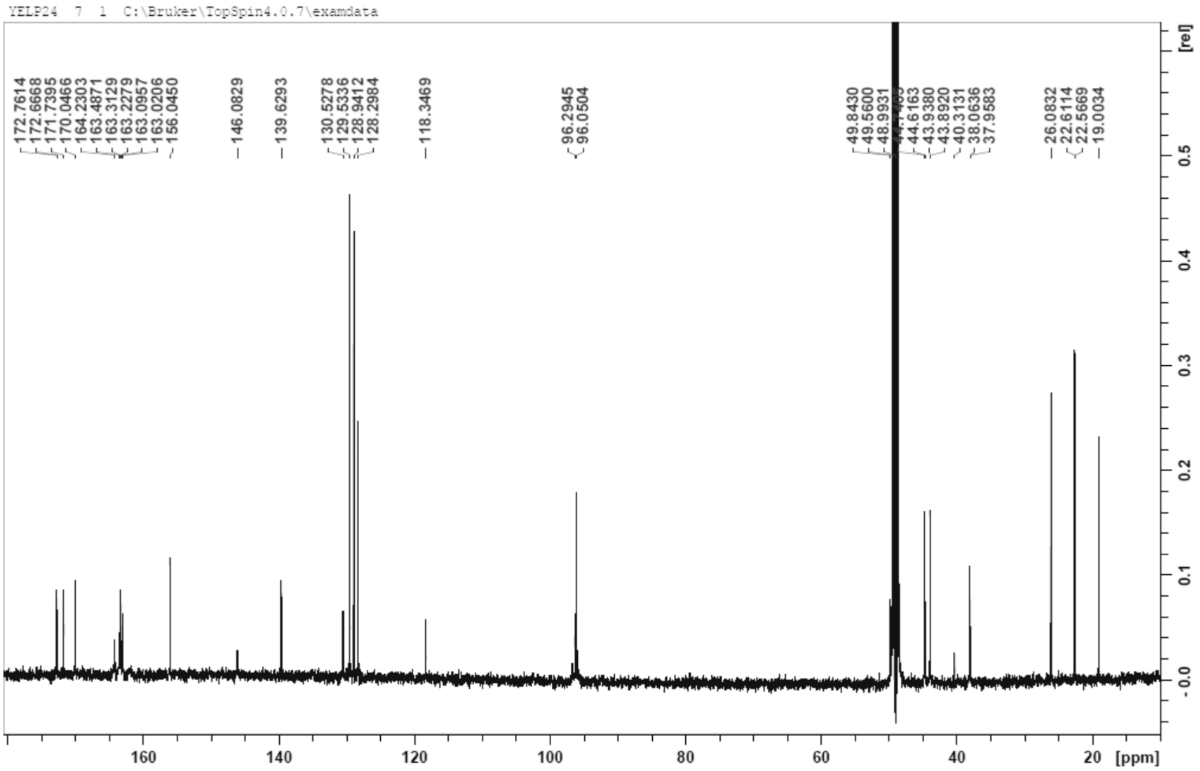

Compound 3d

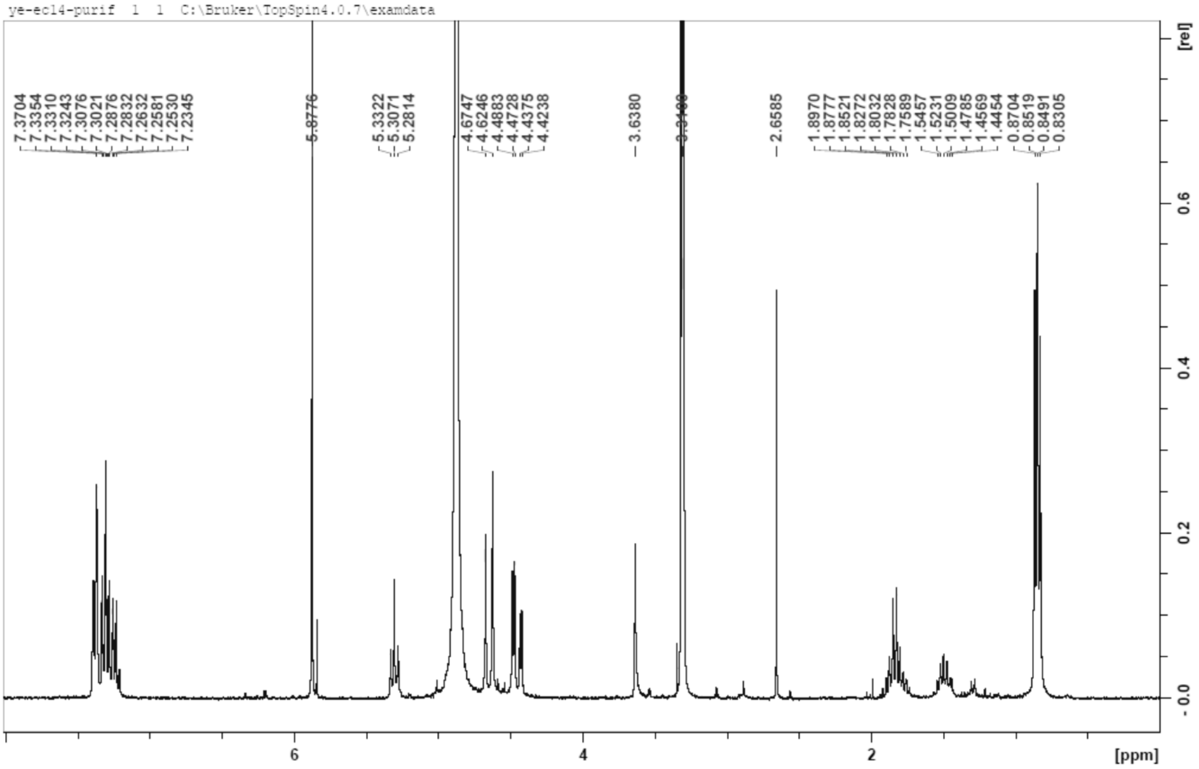
ye-ec14-purif 1 1 C:\Bruker\TopSpin4.0.7\examdata
7.3704
7.3354
7.3310
7.3243
7.3076
7.3021
7.2876
7.2832
7.2632
7.2581
7.2530
7.2345
5.8776
5.3322
5.3071
5.2814
4.6747
4.6246
4.4883
4.4728
4.4375
4.4238
3.6380
2.6585
1.8970
1.8777
1.8521
1.8272
1.8032
1.7828
1.7589
1.5457
1.5231
1.5009
1.4785
1.4569
1.4454
0.8704
0.8519
0.8491
0.8305
[rel]
[ppm]

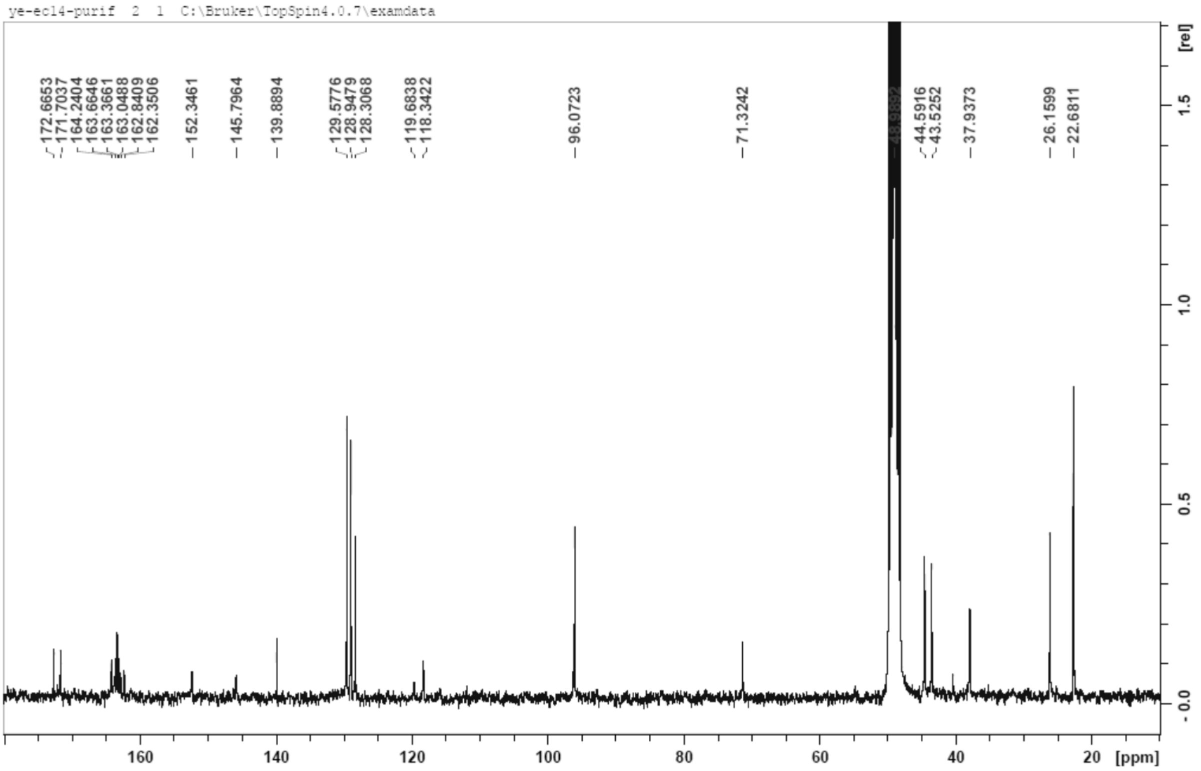
ye-ec14-purif 2 1 C:\Bruker\TopSpin4.0.7\examdata
172.6653
171.7037
164.2404
163.6646
163.3661
163.0488
162.8409
162.3506
152.3461
145.7964
139.8894
129.5776
128.9479
128.3068
119.6838
118.3422
96.0723
71.3242
44.5916
43.5252
37.9373
26.1599
22.6811
[rel]
[ppm]
